# Phylogenomic networks reveal limited phylogenetic range of lateral gene transfer by transduction

**DOI:** 10.1038/ismej.2016.116

**Published:** 2016-09-20

**Authors:** Ovidiu Popa, Giddy Landan, Tal Dagan

**Affiliations:** 1 Institute of General Microbiology, Christian-Albrechts University of Kiel, Kiel, Germany

## Abstract

Bacteriophages are recognized DNA vectors and transduction is considered as a common mechanism of lateral gene transfer (LGT) during microbial evolution. Anecdotal events of phage-mediated gene transfer were studied extensively, however, a coherent evolutionary viewpoint of LGT by transduction, its extent and characteristics, is still lacking. Here we report a large-scale evolutionary reconstruction of transduction events in 3982 genomes. We inferred 17 158 recent transduction events linking donors, phages and recipients into a phylogenomic transduction network view. We find that LGT by transduction is mostly restricted to closely related donors and recipients. Furthermore, a substantial number of the transduction events (9%) are best described as gene duplications that are mediated by mobile DNA vectors. We propose to distinguish this type of paralogy by the term autology. A comparison of donor and recipient genomes revealed that genome similarity is a superior predictor of species connectivity in the network in comparison to common habitat. This indicates that genetic similarity, rather than ecological opportunity, is a driver of successful transduction during microbial evolution. A striking difference in the connectivity pattern of donors and recipients shows that while lysogenic interactions are highly species-specific, the host range for lytic phage infections can be much wider, serving to connect dense clusters of closely related species. Our results thus demonstrate that DNA transfer via transduction occurs within the context of phage–host specificity, but that this tight constraint can be breached, on rare occasions, to produce long-range LGTs of profound evolutionary consequences.

## Introduction

DNA transfer is an important mechanism for natural variation in the prokaryotic domains (Ochman *et al.*, 2000). Recombination at the species level has a role in selective sweeps through the population (Shapiro *et al.*, 2012) while lateral gene transfer across species boundaries has important implications to microbial adaptation and evolutionary transitions (for example, Nelson-Sathi *et al.*, 2015). Viruses that infect bacteria—termed phages—are known vectors of DNA transfer between microbial cells (Zinder and Lederberg, 1952; Halary *et al.*, 2010). Temperate (or lysogenic) phages multiply via the lysogenic cycle, which is established by an integration of the phage genome into the host chromosomes, creating a prophage within the host genome. The phage typically remains dormant within the host and is replicated with the host genome until the lytic cycle is induced. In the lytic cycle new phages are produced using the host metabolism and are released during the host cell lysis (Campbell, 2003). The excision of phage DNA from the host genome and the production of phages may be accompanied by packing of host DNA into the phages, which can then transfer it to the next host in a process that has been termed transduction (Zinder and Lederberg, 1952). Specialized transduction occurs when the phage integrases cleave, in addition to the prophage, bacterial genes that are encoded at the prophage flanking regions. These are packed with the phage DNA into the phages. Generalized transduction occurs when random bacterial DNA is packed into the phages (Weinbauer, 2004). A recent analysis of enterobacterial genomes revealed an extensive domestication of genes encoded in prophages. The prophage domestication process comprises of rapid prophage inactivation followed by a gradual genetic degradation that is marked by a strong purifying selection on the acquired gene sequence followed by their vertical inheritance within the lineage (Bobay *et al.*, 2014).

The frequency of transduction in nature may vary between environments. In marine environment the frequency of transduction has been estimated to range between 1.33 × 10^−7^ and 5.33 × 10^−9^ transductants/plaque forming unit (Jiang and Paul, 1998). In a freshwater environment, a higher transduction frequency ranging between 0.3 × 10^−3^ and 8 × 10^−3^ transductants/plaque forming unit was observed, where 20% of the gene recipients retained their viability (Kenzaka *et al.*, 2010). Phage lethality, as measured by the ratio of phage infection to adsorption, and host specificity may however differ between various phage taxa. For example, cyanophages of diverse taxa are highly host-specific and their interaction is characterized by 100% lethality while heterosiphoviruses have been shown to be adsorbed by a wide range of Pseudoalteromonas strains and their lethality ranges between 10 and 40% (Deng *et al.*, 2012). The realized host range in the wild is however determined not only by the host permissibility but also by the phage–bacteria co-occurrence in the same geographic habitat (Flores *et al.*, 2013).

Bacteria and their parasitic phages are co-evolving in a constant arms race, yet their interaction may include also mutualistic aspects. The beneficial contribution of phage-mediated gene transfer to the host fitness has been documented in diverse environments (Canchaya *et al.*, 2003). For example, genomes of phages that infect marine cyanobacteria have been found to encode components of both photosystem I (Sharon *et al.*, 2009) and photosystem II (Lindell *et al.*, 2005). The elevated dose effect of these gene products within the host is assumed to increase the photosystems recycling efficiency and by that compensate for the energetic cost of phage proliferation (Lindell *et al.*, 2005). Recently sequenced metagenomic samples from hydrothermal vents revealed a high abundance of phages that encode components of the dissimilatory sulfite reductase gene (*rdsr*) (Anantharaman *et al.*, 2014). This gene is essential for sulfur oxidation and may confer an energetic advantage to chemolythoautotrophic bacteria that typically inhabit such environments. In addition to the transfer of metabolic functions between closely related hosts, phages have been found to mediate intergeneric gene transfer across species boundaries as exemplified in the transfer of toxin genes between *Staphylococcus aureus* and *Listeria monocytogenes* in raw milk (Chen and Novick, 2009).

Bacterial genomes that include a prophage may be considered as recipients in gene transfer events. Bacterial genes in prophages are the result of gene acquisition by transduction and their origin can be identified by homology and phylogenetic analysis. Here we study the extent of phage-mediated gene transfer during microbial evolution using network approach. The networks are composed of donors, phages and recipients that are connected by recent transduction events reconstructed from genomic data. Structural properties of the network supply a large-scale view of barriers for transduction and gene transferability by phages in nature.

## Materials and methods

### Data

Genomes of 2103 complete and 1879 draft prokaryotic strains were downloaded from GenBank (version 10/2012). Annotation of 14 920 prophages encoded in 8540 genomic sequences was downloaded from PHAST database (version 10/2012) (Zhou *et al.*, 2011). PHAST entries not found in GenBank were discarded. This resulted in 9468 annotated prophages encoded in 1330 complete and 1281 draft genomes. Coding sequences (CDSs) in PHAST database are classified into viral or bacterial according to their homology to known viral or bacterial genes (Zhou *et al.*, 2011). Prophages encoding only phage genes were excluded. The remaining 9201 (97%) prophages encode 281 616 CDSs, of which 89 234 (32%) are classified as bacterial genes. Prophages in the prokaryotic genomes were annotated in addition with PhiSpy (Akhter *et al.*, 2012) and VirSorter (Roux *et al.*, 2015a).

Prophages were clustered into orthologous prophage clusters (Bobay *et al.*, 2013) based on their gene content. The first step included an all-against-all BLAST of prophage protein sequences. Reciprocal best BLAST hits (Tatusov *et al.*, 1997; Wolf and Koonin, 2012) with *E*-value <1 × 10^−10^ were aligned globally using needle (Rice *et al.*, 2000). Pairs having <95% amino-acid identity were excluded. The remaining CDSs were clustered into orthologous protein families using MCL (Enright *et al.*, 2002) with default parameters. Pairwise prophage gene content similarity was calculated from frequencies of shared protein families using the Jaccard index. Prophage similarity as estimated using the hypergeometric similarity measure (Fuxman Bass *et al.*, 2013) was significantly correlated with the Jaccard measure (*r*=0.96, *P*<10^−15^; excluding zero values) and we chose to proceed with the former. In agreement with previous reports (Bobay *et al.*, 2014), we found that high prophage gene content similarity thresholds are too stringent, whereas low values lead to clusters of distantly related prophages. Here we apply a threshold of >70% shared families for the classification of orthologous prophages (Bobay *et al.*, 2014). Our pipeline clustered 6494 (43.5%) prophages into 2397 orthologous prophages those are considered as a phage entity in our evolutionary reconstruction. The remaining 8426 unclustered prophages are designated as singleton phages.

### Donor inference

The donor inference procedure operates within the framework of orthologous protein families and is assisted by a phylogenetic tree. Bacterial genes encoded within prophages were clustered into orthologous protein families. In the first step, we searched for homologs to the prophage genes in the GenBank genomes using BLAST. Using an *E*-value <1 × 10^−10^ threshold, we identified 3 908 830 homologous sequences to 75 172 of the query genes, whereas no homologs were detected for the remaining 14 197 (15.89%) queries. Protein pairs were aligned globally with needle (Rice *et al.*, 2000). Protein sequences were considered as homologs if they had at least 90% global amino-acid similarity to the query sequence. This results in a data set of 42 760 (57%) prophage bacterial genes and 252 159 homologs. The protein sequences were clustered into orthologous protein families using MCL (Enright *et al.*, 2002) to yield 20 904 protein families with at least two proteins. Protein clusters containing at least three protein sequences (12 611) were aligned using MAFFT (Katoh and Standley, 2013) and maximum likelihood trees were reconstructed using PhyML3 (Guindon *et al.*, 2010) with the LG model. Three trees were rooted using the midpoint criteria. In protein families with multiple recipient genes, we examined the monophyly of recipient genes, and when these were paraphyletic (2205 trees, 17.48%), we tested for the likelihood of an alternative tree with recipients consolidated into one clade. For this we used CONSEL (Shimodaira and Hasegawa, 2001) with the approximately unbiased test and the multi-scale bootstrap technique. Of the reconfigured trees, 829 (37.6%) were not significantly less likely than the original tree (approximately unbiased test, *P*⩾0.05) and were retained for downstream analyses. For each gene acquired by transduction, we identified the most likely gene donor as the genome bearing a homologous gene, which is the sister taxon of the acquired gene in the phylogenetic tree or the unique homolog in the case of clusters with two members. Trees where the donors or recipients formed a clade, rather than consisting of a single taxon, were excluded from further analysis. An overview of the various steps in the steps in the analysis is given in Supplementary Table S1.

### Network construction

Donor–recipient relations were coded into the directed lateral gene transfer (dLGT) unipartite network, in which nodes represent bacterial species and edges lateral gene transfers mediated by transduction. Bacteria–phage relations were coded into a bipartite directed network, where nodes represent either phages (5064 nodes) or bacterial species (3982 nodes). This enables partitioning of the network into two subsets: the recipient subset (R-dLGT) that consists of directed edges from prophages to their host bacterial species (that is, the recipients); and the donor subset (D-dLGT) that consists of directed edges from donor bacterial nodes to phages (that is, the transfer vector). As in the dLGT, edge weights correspond to the number of transferred genes.

### Network randomization

Randomization of the dLGT network was carried out using the switching methodology (Artzy-Randrup and Stone, 2005), which rewires the weighted edges while preserving the in- and out-degree of each node. The method was implemented in an in-house MatLab (version R2015b; The Mathworks Inc.) script and used to generate 1000 randomly connected networks.

### Genome similarity measures

Genome sequence similarity (*S*_gs_) between a recipient and a donor was calculated as the Jaccard coefficient based on the proportion of 20 bp segments common to the two genomes (using MUMmer (Kurtz *et al.*, 2004)). Proteome similarity (*S*_pr_) between bacterial species was similarly calculated as the Jaccard index of identical segments, but restricted to segments that have an overlap at least of 10% within a protein-coding region. GC content similarity (*S*_GC_) was calculated as follows: 100−|%GC_recipient_−%GC_donor_| (Popa *et al.*, 2011). The genome codon usage distance (*D*_CU_) was calculated as the Euclidean distance 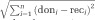 between the vectors of relative codon frequencies per amino acid within the donor and recipient genomes.

### Synonymous and non-synonymous substitution rates

The number of non-synonymous (*d*_N_) and synonymous (*d*_S_) substitutions, and their ratio *ω*, was calculated using the branch model implemented in PAML (Yang, 2007). We used ‘model 2' of PAML, allowing *ω* (*d*_N_/*d*_S_) to vary among the donor branches, recipient branches and the remaining (background) branches. For the special case of gene duplication (self-donor recipient loops), we estimated *d*_N_, *d*_S_ and *ω* using the software package PAL2NAL (Suyama *et al.*, 2006). PAL2NAL creates a codon alignment from a pair of protein and their corresponding DNA sequences and calculated the *d*_N_ and *d*_S_ values using PAML (Yang, 2007). Codon adaptation index (Sharp and Li, 1987) for each gene was calculated using the EMBOSS package (Rice *et al.,* 2000).

### Function, habitat and ICTV classification

Functional classification of each cluster was derived from the Clusters of Orthologous Groups database (Tatusov *et al.*, 1997) by a majority vote of cluster members. The habitat classification of donor and recipient nodes was extracted from the GOLD database version March 2014. We defined 11 main habitat classes using the combination of isolation place and ecology annotation. Putative International Committee on Taxonomy of Viruses (ICTV) annotation was derived from the majority vote of ICTV labels of prophage genes. Laterally transferred toxin–antitoxin operons were surveyed using PanDaTox (Amitai and Sorek, 2012) as a query. An additional survey for laterally transferred genes for antibiotics resistance was performed using the genes in CARD database (McArthur *et al.*, 2013) as queries.

### Statistics and visualization

All statistical calculations were done using the Statistics toolbox in MatLab. The network layout was calculated with Cytoscape (Shannon *et al.*, 2003) using the force-directed graph-drawing module.

## Results

### The transduction network

**Figure 1:**
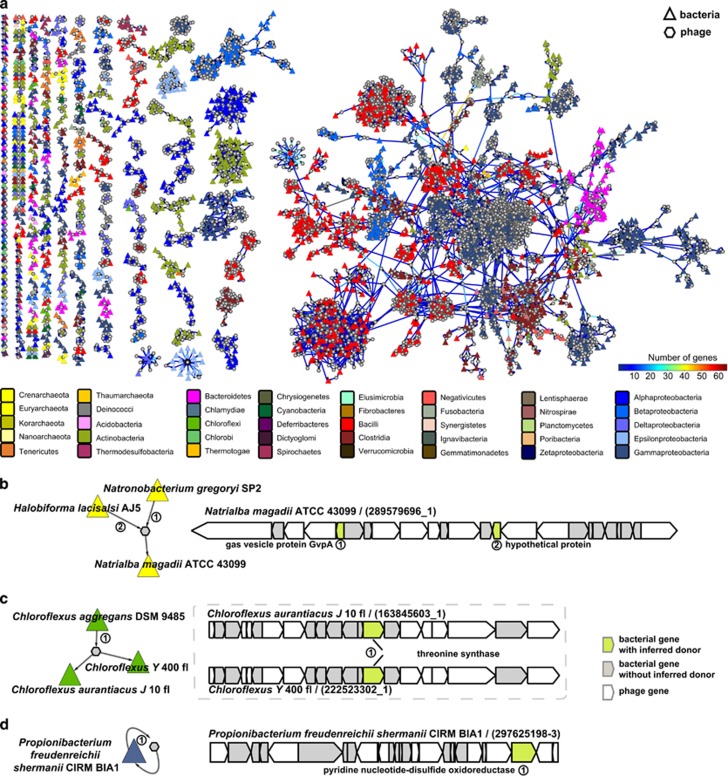
The directed transduction network. (**a**) Graphic representation of the directed, bi-partite, transduction network (dLGT) reconstructed from transduction events, where a single most likely donor could be identified. The nodes correspond to bacterial genomes (triangles) and phages (hexagons). Bacterial nodes are colored by their taxonomic group. Directed edges correspond to genes that were transferred between bacterial genomes via a phage. Bacteria to phage edges describe uptake of genes during lytic infection (D-dLGT) while phage to bacteria edges designate the acquisition of genes in prophages (lysogenic interactions, R-dLGT). Edge color corresponds to the number of transferred genes (see color bar). (**b**–**d**) Detailed examples showing enlarged parts the dLGT, including bacterial species names and the prophages genomic maps. Circled numbers identify specific genes in both views. Putative ICTV assignments are listed in Supplementary Table S3.

To study the general properties of LGT by transduction we combine individual donor–recipient inferences into a network representation. Transduction events are characterized by two distinct phases: the uptake of a gene from a donor into a phage and the acquisition of a gene as part of a prophage by the recipient. We constructed a dLGT network that includes two types of entities: bacteria and phages. A directed edge from a phage node to a bacteria node designates a gene acquisition following transduction as inferred from the prophage annotation, where the bacteria node is the recipient. A directed edge from a bacteria node to a phage node specifies the acquired gene origin as inferred from the phylogenetic analysis, where the bacteria node is the donor. We analyzed a total of 2103 finished and 1879 draft microbial genomes, including a total of 9468 annotated complete and partial prophages annotated in PHAST (Zhou *et al.*, 2011). Alternative prophage annotations calculated with PhiSpy (Akhter *et al.*, 2012) and VirSorter (Roux *et al.*, 2015a) resulted in a larger collection of putative prophages, but with lesser consistency among the three methods than PHAST (Supplementary Figure S1). Applying conservative sequence similarity thresholds in the different inference stages, our approach identified 17 158 transduction events, where donor and recipient are specified. Constructing the network from those events, where a single most likely donor was identified yielded a dLGT network (Popa *et al.*, 2011), comprising 2573 bacteria and 4650 phage nodes that are connected by 15 298 edges summarizing all 17 158 transduction events (Figure 1a; Supplementary Table S2). Edge weight in the network is calculated as the total number of genes that were transferred between the bacteria and phage nodes.

The dLGT network comprises a large component of 4982 nodes, including 1538 bacteria and 3444 phages. The remaining nodes in the network fall into 326 smaller clusters including, on average, three bacteria and four phages. For example, the *Natrialba magadii* φCH1 virus has a temperate interaction with the chemoorganotrophic euryarchaeon *N. magadii* isolated from Magadi lake in Kenya (Klein *et al.*, 2002). The virus encodes a total of 24 bacterial genes. Our inference algorithm yielded putative donors for two of those genes. One gene, annotated as a hypothetical protein, was putatively acquired from *Halobiforma lacisalsi*, an extreme halophylic archaeon. The second gene, annotated as a gas vesicle protein, was putatively acquired from *Natronobacterium gregoryi*, a haloalkaliphilic euryarchaeon (Figure 1b). In another cluster we identified orthologous prophages that are encoded within the genomes of two chloroflexi strains: *Chloroflexus aurantiacus* J-10-fl and *Chloroflexus* sp. Y400-fl. The two prophages have a 100% match of their protein content when applying a sequence similarity threshold of 95% identical amino acids, thus they are considered as orthologous prophages. One of the eleven bacterial genes encoded in this prophage is annotated as a threonine synthase and was putatively acquired by the phage from *Chloroflexus aggregans* (Figure 1c). This small cluster exemplifies how phage-mediated laterally transferred genes can enter the lineage.

### Phage-mediated gene duplication—autology

Although most of the genes acquired via prophages are xenologs, the network reveals a substantial number of genes, where the recipient genome is also the donor (for example, Figure 1d). Thus, per definition these genes are paralogous rather than xenologous genes. We suggest terming such genes autologs. According to our definition, an autologous gene is the result of gene duplication that is mediated by a mobile DNA vector, where the donor is also the recipient. Our analysis revealed 1550 (9%) autologous genes that are distributed over 543 (21%) microbial genomes. About half of the gene duplications in the network are of a single gene and up to a maximum of 48 genes in *Magnetococcus* sp. MC-1. Of the self-donor recipients, 72% are connected to a single phage (Supplementary Figure S2). A maximum of nine phages are connected by a self-edge to *Methylobacterium nodulans* ORS_2060.

Of the 1550 autologs, 697 have no nucleotide substitutions at all, whereas the remaining 54% autologs show the hallmarks of gene duplications. They contain significantly more synonymous than non-synonymous substitutions (*P*<10^−15^, using paired-Wilcoxon test). The median *d*_N_/*d*_S_ ratio (*ω*) is 0.12, which is significantly larger than the observed for the *bona fide* gene acquisitions (*ω*=0.09, *P*=3 × 10^−13^, using Wilcoxon test). Moreover, the codon adaptation index is significantly smaller for the prophage gene than the genomic copy (*P*=0.035, using paired-Wilcoxon test). These observations are consistent with the observed relaxation of purifying selection in Entrobacteriales prophages (Bobay *et al.*, 2014).

### Donor and recipient components

**Figure 2:**
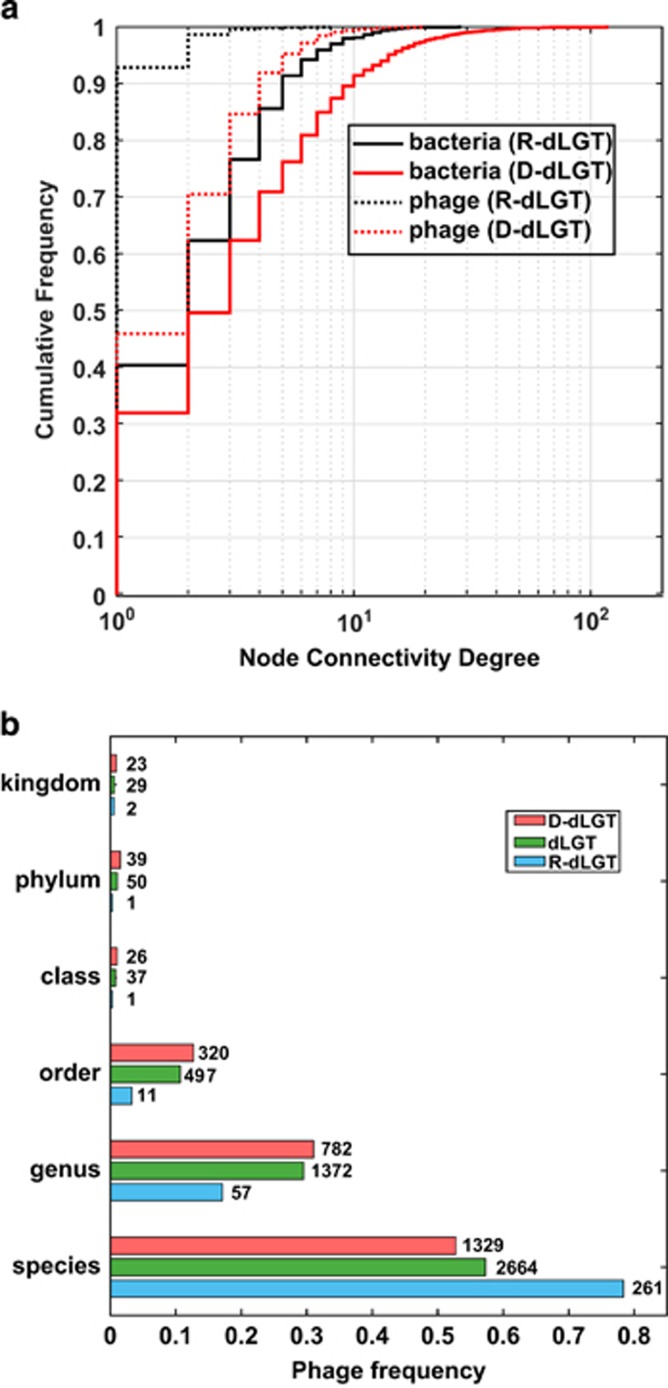
Connectivity and taxonomic distribution. (**a**) Cumulative distribution of node connectivity degree. (**b**) Taxonomic distribution of donors and recipients.

Structural properties of the dLGT network are the result of two different phage–bacteria interaction modes. Phages connected to recipients represent a lysogenic interaction that involves temperate phages and their hosts. Links between donors and phages are evidence for a lytic phage infection, where donors connected to the same phage designate the putative hosts of that phage. The network thus combines two components: edges that link donors to phages correspond to gene uptake by phages, whereas edges that connect phages to recipients correspond to gene acquisition by bacterial recipients. Large-scale structural differences between the two components reveal the differential contribution of lytic and temperate phage–bacteria interactions to transduction dynamics during microbial evolution. The donor and recipient parts of the network, termed here D-dLGT and R-dLGT, respectively, comprise a similar number of bacteria and phage nodes (Supplementary Table S2). Yet, the node connectivity degree is significantly larger in the D-dLGT in comparison with the R-dLGT for both phage and bacteria nodes (D-dLGT, *P*<10^−15^; R-dLGT., *P*<10^−15^, using Kolmogorov–Smirnov test; Figure 2a). Consequently the D-dLGT nodes are more densely interconnected in comparison with nodes in the R-dLGT network. Most (93%) of the phages in the R-dLGT are connected to a single recipient node and at most to eight recipients (Figure 2a). Only 46% (2131) of phages in the D-dLGT network are connected to a single donor node, while 25% (1146) phages are connected to two donors and the remaining 29% (1373) phages are connected to three donors or more (Figure 2a).

It is noteworthy that highly connected phages include genes that have the potential to be beneficial for the recipient. The most connected phage in the D-dLGT network encodes 29 genes of bacterial origin for which we identified 20 Enterobacteriales donors (Supplementary Figure S3; Supplementary Table S4; PhageID: 10223). The phage encodes the MazE/F toxin–antitoxin system that can mediates cell growth arrest and was shown to increase the persistence and survival of *Escherichia coli* under antibiotic stress (Zhang *et al.*, 2005). Our analysis further uncovered the transfer of 73 toxin–antitoxin genes mediated by 32 phages (Supplementary Table S5). These transduction events suggest that phages may encode for addiction mechanisms similarly to plasmids. Another highly connected phage in the D-dLGT network is connected to 19 Enterobacteriales species (Supplementary Figure S3; Supplementary Table S4; PhageID: 11150). The phage encodes for *mdtH*, a multidrug resistance gene that confers resistance to norfloxacin and enoxacin (Nishino and Yamaguchi, 2001). Transferred genes in the network include additional 46 genes coding for a broad range of antibiotic resistance (Supplementary Table S6) demonstrating a putative role of phages in the spread of antibiotics resistance. The most connected phage in the R-dLGT is connected to eight *Bacillus* recipients (for details see Supplementary Figure S3; Supplementary Table S4; PhageID:5009). The phage contains eight genes of bacterial origin. One of those, *bclA*, encodes for a spore surface glycoprotein in *Bacillus anthracis* (Sylvestre *et al.*, 2002).

The different bacteria and phage connectivity pattern of the R-dLGT and D-dLGT is evident also in their global structure. The D-dLGT contains significantly less connected components in comparison to the R-dLGT. Furthermore, nodes in the recipient network are clustered into significantly smaller components in comparison with the donor network (*P*=7 × 10^−10^, using Kolmogorov–Smirnov test) and the number of nodes in the D-dLGT largest component is 25-fold larger in comparison with that of the R-dLGT largest component (Supplementary Table S2). In consequence, edge weights in the D-dLGT are significantly lower in comparison with the R-dLGT (*P*<10^−15^, using Kolmogorov–Smirnov test) with medians of single gene per donor edge and two genes per recipient edge (Supplementary Table S2). The R-dLGT comprises 230 (4.6%) edges with an edge weight ⩾10. In the D-dLGT network, for comparison, we observe only 79 (0.77%) edges having an edge weight ⩾10 (Supplementary Figure S4).

The different structural properties of the donor and recipient network components suggest that gene transfer into hosts during the lysogenic cycle usually comprises several genes, while gene uptake from hosts into the phage genome during lytic infection typically include a single gene. Yet, the high connectivity of the D-LGT network indicates that the lytic interactions serve as linkers among clusters of highly connected recipients, thus they constitute an important contribution to the global consolidation of the transduction network.

### Host range in the transduction network

Phages that are linked to more than one donor or recipient in the network supply an insight into the phage host range. In the D-dLGT component, about half of the phages (2519, 54.17%) are connected to multiple donors. Most of these phages are connected to donors of the same species (1329, 53%) or genera (782, 31%) revealing a very narrow taxonomic host range at the donor side (Figure 2b). Only 22 phages in the D-dLGT network are connected to two donors that are members of different phyla, 20 of which are connected to Firmicutes strains (Supplementary Table S7). A single phage is connected to three donors from different phyla including *Bacteroides* sp. 3_1_33FAA and *Clostridium* sp. M62/1 that were isolated from the human gastrointestinal tract and *Cardiobacterium hominis* ATCC 15826 (Gammaproteobacteria) that was isolated from the human cardiovascular system (Human Microbiome Project Consortium, 2012) (Supplementary Table S7; PhageID: 9283).

Phages connected to more than a single recipient in the R-dLGT network (333; 7%) show even stronger species-specificity, with most phages (261, 78%) connected to recipients classified into the same species (Figure 2b). A total of 57 (17%) phages are linked to recipients from different species within the same genus (Supplementary Figure S3; Supplementary Table S4; PhageIDs: 5548 and 5273). Only 11 (3.3%) phages are found in recipients of different genera within the same taxonomic order. The rare inter-generic transduction events include a phage connected to two Clostridiales recipients: *Blautia hansenii* DSM 20583 and *Ruminococcus gnavus* ATCC 29149 (Supplementary Figure S3; Supplementary Table S4; PhageID: 5915). Both strains were isolated from the human digestive system (Human Microbiome Project Consortium, 2012), hence they probably share a common habitat.

A single phage links two recipients from different classes within the Firmicutes phylum, *Clostridium M62-1* and the *Lactobacillus ruminis* ATCC 25644, both isolated from the human gastrointestinal tract (Human Microbiome Project Consortium, 2012; Supplementary Figure S3; Supplementary Table S4; PhageID: 6299). Only two phages link to recipients from different phyla (Supplementary Figure S3; Supplementary Table S4; PhageIDs: 5805 and 6260). One of those connects the *Bifidobacterium pseudocatenulatum* DSM 20438 (phylum: Actinobacteria) and *Parvimonas micra* ATCC 33270 (phylum: Firmicutes), both isolated from the human gastrointestinal tract (Human Microbiome Project Consortium, 2012).

The narrow taxonomic range of multiple donors and recipients observed in the network components is in agreement with experimental observations of phage species-specificity (Hyman and Abedon, 2010) and is expected from the tight phage–host co-evolutionary dynamics. Our results reveal however several genomic footprints of rare cross-species infections. Many of these examples are observed in microbial genomes sequenced as part of the human microbiome project, thus it is possible that the high sampling density of that habitat facilitated that recovery of those rare interactions.

### Barriers for gene transfer by transduction

The majority of phages (2664, 64%) connect donors and recipient from different strains of the same species (Figure 2b). These phage-mediated DNA transfers are best viewed as genetic recombination rather than lateral gene transfer events. A Siphoviridae phage connected to multiple *Vibrio cholera* strains illustrated this phenomenon (Supplementary Figure S3; Supplementary Table S4; PhageID: 8390). The phage encodes the *nqr* operon that has an important function in the bioenergetics and homeostasis of *V. cholerae* (Barquera *et al.*, 2002). The frequency of observed LGTs decreases markedly when the donor–recipient taxonomic separation increases (Figure 2b). At the inter-domain level, only a single phage was observed, connecting *Methanobrevibacter smithii* DSM 2374 as the recipient with *Bacillus cereus* Rock3–28 as the donor (Supplementary Figure S3; Supplementary Table S4; PhageID: 9888). The recipient strain was isolated from human feces (Human Microbiome Project Consortium, 2012), whereas the donor was isolated from the soil (Zwick *et al.*, 2012). The prophage includes a gene encoding for tetracycline resistance that has 100% identical amino acids to the gene encoded in *B. cereus*. To our knowledge, this is the first genomic evidence for transduction of an archaebacterium by a eubacterial bacteriophage; hence, this putative inter-domain transfer represents a very exceptional event.

Barriers for transduction may be related to the genetic requirements for a successful gene acquisition and the ecological co-occurrence of the connected partners. In contrast to transduction, in transformation and conjugation the integration of acquired DNA into the recipient genome is mediated by homologous recombination and therefore depends on sequence similarity between the donor and recipient (Thomas and Nielsen, 2005). During transduction, however, the acquired DNA is integrated into the recipient genome using the phage mechanism (Thomas and Nielsen, 2005), hence no such dependency is expected. To test for genetic barriers to DNA transfer by transduction we calculated the genome similarity between donors and recipients using four measures. Genome similarity (*S*_GS_) is calculated as the Jaccard index of identical ⩽20 bp sequences between the donor and recipient genomes. CDS similarity (*S*_CDS_) is calculated similarly but is restricted to protein CDSs. Codon usage distance (*D*_CU_) is calculated as the Euclidean distance between the relative codon frequencies within the donor and recipient genomes. GC content similarity (*S*_GC_) is calculated from the genomic content of guanine and cytosine in the donor and recipient genomes. The distribution of all similarity measures was compared between the dLGT network and a set of 1000 networks, where the edges have been randomly shuffled.

**Figure 3:**
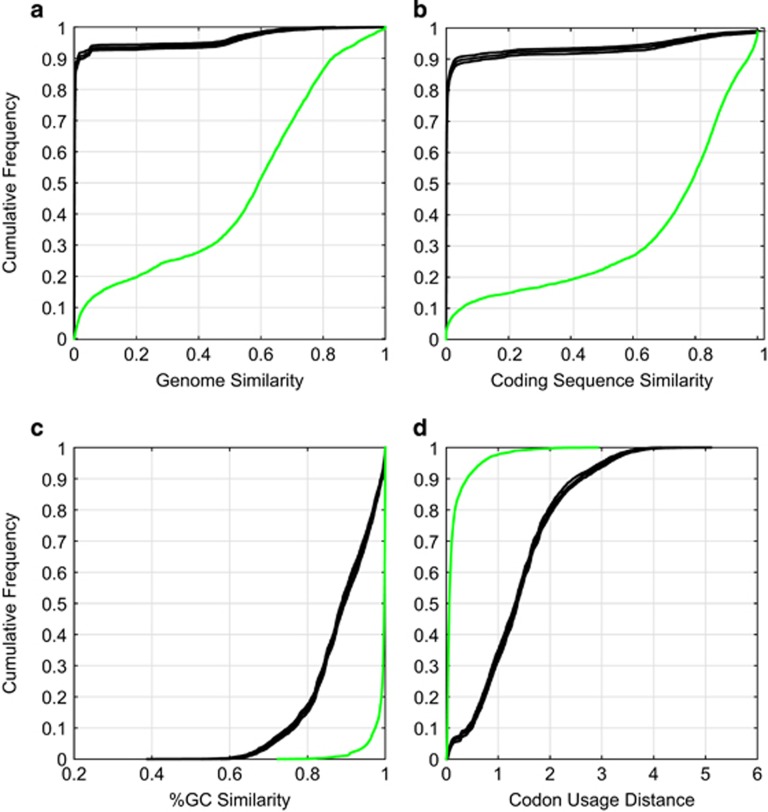
Donor–recipient genome similarity. Cumulative distribution of donor–recipient genome similarity measures in the dLGT network (green) and 1000 randomized networks (black). (**a**) Genome sequence similarity, (**b**) CDS similarity, (**c**) GC content similarity and (**d**) codon usage distance.

We find that donors and recipients connected in the dLGT network are significantly more similar to each other than expected by chance using all similarity measures (Figure 3). The four similarity measures are correlated—closely related genomes will score high on each measure, yet it is of interest to grade their importance as barriers to LGT. To this end, we consider each pairwise similarity measure as a predictor of the connectedness state of the pair of species, and conduct a receiver-operating characteristics analysis (for example, Fawcett, 2006). We find that genome similarity is the best predictor for dLGT connectedness, with an area under the receiver-operating characteristics curve (AUC) of 0.99, and an optimal discrimination of 0.97 true-positive rate (TPR) and 0.03 false-positive rate (FPR). The next best measure is codon usage distance (AUC 0.98; TPR 0.93; FPR 0.03), followed closely by CDS similarity (AUC 0.96; TPR 0.94; FPR 0.04). GC content similarity is an inferior predictor in comparison with the other measures (AUC 0.95; TPR 0.87; FPR 0.08). Restricting the analysis to a subset of prophages that were detected also by PhiSpy (Akhter *et al.*, 2012) or VirSorter (Roux *et al.*, 2015a) prophage annotation tools reveals an even sharper deviation from the expected by chance (Supplementary Figure S5). Our results demonstrate that low donor–recipient genome similarity is an important barrier that constrains the extent of LGT via transduction.

Another possible barrier for transduction is the need for ecological co-occurrence of donors, phages and recipients. This barrier may be partially breached by phage mobility that is thought to enable the transfer of genetic material between donors and recipients across a larger spatial separation compared with other LGT mechanism that are dependent of physical proximity (Majewski, 2001). Donor–recipient pairs share the same habitat in 3330 (44%) cases, of which the largest group (1383, 41%) are members of the ‘human-associated' habitat group. In the remaining 4187 (56%) donor–recipient pairs classified in different habitat groups (cross-habitat transfer events), we observed the majority (858, 20.49%) of links between the donor group ‘host' and the recipient group ‘human-associated' bacteria (Supplementary Figure S6). To evaluate whether these values are different from the expectation given that habitat sampling is heavily skewed toward certain habitats, we estimated the expected within- and cross-habitat frequencies from 1000 randomized dLGT networks. Links between donors and recipients from the same habitat are significantly overrepresented in the dLGT network, with the corollary that most cross-habitat links are occurring at a lower frequency than expected. However, some habitats do show a higher than expected cross-habitat LGT frequencies (Supplementary Figure S6). For example, we found 51 (expected 17) links between ‘soil and sediment' and the ‘plant' group and 41 (expected 19) transfers between the habitat ‘plant' and ‘soil and sediment' group. Forty-eight percent of these transfers are intra-specific and 95% are intra-generic. Indeed, habitat sharing is only a weak predictor of species connectedness, with equivalent AUC of only 0.64 (TPR 0.44; FPR 0.17). Our analysis thus reveals that the barriers for gene transfer via transduction are primarily genetic while ecological barriers have a smaller role.

### Functional classification and evolutionary constraints

The functional composition of dLGT genes is significantly different than that of the analyzed bacterial genomes (*P*<10^−15^, using *χ*^2^-test). Information processing functions are overrepresented in the network, whereas cellular processes and metabolism functions are depleted (Supplementary Figure S7). Of the genes that could be classified into putative functions (2274, 13%), 42% perform metabolism functions, whereas 35% were involved in information processing; most of those are annotated as transcription genes. Another 23% of the genes were classified into cellular processes, with a majority of cell wall and membrane biogenesis function (Supplementary Figure S7). Interestingly, information genes are transferred between less similar donors and recipients than the other functions, while metabolism and cellular processes genes are transferred between equally similar donors and recipients (*α*=0.05, using Tukey test). This observation may be attributed to the universality of information processing genes. In addition, we examined whether the functional categories distribute differently when considering the habitat of the host or the taxonomic classification, with the difference that the analysis was restricted to the three main functional classes due to limited sample size (using *χ*^2^-test and false discovery rate (FDR) of 5%). None of the habitats was found to be significantly different from the others in terms of the functional classification of the dLGT genes. Only two taxonomic groups appeared to deviate from the common functional distribution. In Actinobacteria we observed an excess of metabolic genes and paucity of cellular processes genes; in Epsilonproteobacteria we observed an excess of cellular processes genes and paucity of information genes.

The nucleotide substitution pattern of genes in the transduction network indicates that their acquisition was very recent or that they evolve under extremely strong purifying selection. Half (52%) of the donor–recipient pairs have no nucleotide substitutions at all. Comparing the nucleotide substitution rate between the donor and recipient lineages for the remaining 48%, we observe a very slight and not significant increase in the recipient lineage rates (Supplementary Table S8). The *ω* ratio is also not significantly different between the two lineages and in 95% of the genes is below 0.5 in both lineages. Together these observations suggest that the strength of purifying selection in recipient lineages remains similar to that in the donor lineages with no apparent relaxation of selective constraints or nonfunctionalization. The great majority (95%) of bacterial genes that are encoded in prophages are single-copy genes, that is, there is no pre-existing homologous gene in the recipient genome. Taken together with the evidence for gene functionality, this suggests that most transduction events result in an acquisition of a new function. Furthermore, it could indicate that the accessibility of the host to the new function is maintained as long as the lysogenic interaction with the phage is maintained.

## Discussion

Here we study the contribution of phage-mediated gene transfer to microbial genome evolution. The transduction network reconstruction revealed a substantial frequency of autologs. Autologs may be the result of recurrent infections, where both donor and recipient are members of one lineage. Transduction within the lineage may thus contribute to protein family expansion in bacteria, which was proposed to be mediated more often by LGT than by gene duplication (Hooper and Berg, 2003; Treangen and Rocha, 2011). Indeed, we have to assume that low sampling density may obscure a gene donor among closely related strains, whose genome has not yet been sequenced. Nevertheless, the high sequence similarity and lack of alternative homologs besides the recipient genomic copy indicate that autologs originate from within the pan-genome.

The topological differences between the donor and recipient network components suggest that host-specificity is much more prevalent in lysogenic interactions and that phages have a broader host range for lytic infection. Because lysogenic phages are highly dependent on the host cellular processes (for example, Tal *et al.*, 2014) it is likely that closely related strains having a similar genetic background can have a lysogenic interaction with the same phage.

**Figure 4:**
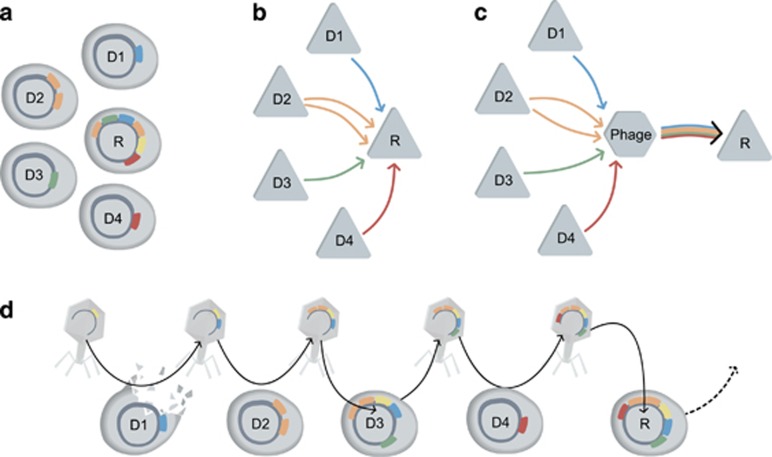
Observation, network and interpretation. (**a**) The raw observations are genes encoded in prophages within recipient genomes (R) and their homologs in microbial genomes that are identified as the donors (D1–D4). (**b**) Unipartite genome-only network coding of the donor–recipient relationships. (**c**) Bipartite coding, including the phage as the mobile element facilitating the transfer. Note the bundling of multiple edges into a single transduction event. (**d**) One possible interpretation of the network in **c** as a historical scenario, depicting consecutive gene uptake during lytic (D1, D2 and D4) or lysogenic (D4 and R; penetrating arrow) cycles, followed by transduction into the recipient. The prophage in the recipient is a current snapshot of an ongoing process.

Previous studies of LGT dynamics estimated that most LGT events involve very few genes while bulk transfers are relatively rare (Kunin *et al.*, 2005; Popa *et al.*, 2011). Transduction, however, is known to involve several genes simultaneously. This is apparent in our network approach and its distinction between donors, mobile elements and recipients. This inclusion of the mobile element allows us to group genes that arrived into the recipient in a single transduction event, as opposed to genome-only networks that would depict the same history as multiplicity of edges into the recipient (Figure 4). On the donor side, on the other hand, we still observe multiple donors for the same transduction event. Hence, transduction is characterized by bulk acquisition of mosaics of genes from multiple donors.

The dLGT network reveals the existence of strong taxonomic and genetic barriers for phage-mediated lateral gene transfer. Previous studies advanced the view that gene transfer during microbial evolution is largely determined by ecological rather than phylogenetic factors (Smillie *et al.*, 2011). Although we do find an overrepresentation of transfers within habitats, habitat sharing is only a weak predictor of species connectedness and is inferior to all sequence-derived similarity measures. The significant high codon usage similarity of donors and recipients is consistent with previous observations of non-random codon usage in phage genomes, leading to the suggestion that phage codon usage is adapted to that of the host (Sharp *et al.*, 1985; Roux *et al.*, 2015b). Previous studies have emphasized the importance of codon usage similarity for LGT, suggesting that high codon usage similarity between acquired genes and the recipient genome will increase the xenolog retention prospects (Medrano-Soto *et al.*, 2004; Tuller *et al.*, 2011). Our results reveal that genes acquired via transduction originate in genomes with a similar codon usage to that of the recipient; hence, the translational barrier for their adaptation is expected to be rather low. Several temperate phages have been reported to encode genes that are transcribed independently from the prophage excision mechanism (Cumby *et al.*, 2012). Thus, the transcriptional regulation of genes acquired via transduction is likely to be promoted by prophage-encoded promoters so that genes acquired by transduction are functional upon acquisition.

In summary, our results demonstrate that LGT via transduction occurs within the tight constraints of phage–host specificity. Consequently, transduction is probably more important in the evolutionary context of genetic recombination within the species, and selfing in the case of autologs, than in the evolutionary context of long-range gene transfers between distinct lineages. LGT is commonly viewed as a source for reticulated events that reduce the tree signal during prokaryotic evolution (Martin, 1999). Our current results show that the reticulated events introduced by transduction affect mostly clades of closely related species and very rarely do they traverse the tree and disrupt its global topology.
