# Supplementary material for: Phylogenomic networks reveal limited phylogenetic range of lateral gene transfer by transduction
Source: ISME J. 2016 Sep 20;11(2):543–54. doi: 10.1038/ismej.2016.116 (PMC5183456; doi:10.1038/ismej.2016.116)
Supplement: Supplementary Figures [file ismej2016116x1.pdf]

Phylogenomic networks reveal limited phylogenetic range of lateral gene transfer by transduction

Ovidiu Popa[1,2], Giddy Landan[1], Tal Dagan[1]

[1] Institute of General Microbiology, Christian-Albrechts University of Kiel, Am Botanischen Garten 11, 24118 Kiel, Germany. [2] Present address: Institute of Quantitative and Theoretical Biology, Heinrich-Heine-University of Düsseldorf, Universitätsstraße 1, D-40225 Düsseldorf, Germany

SUPLAMENTAL FIGURES

Figure S1. Comparison of three different phage prediction methods: PHAST (Zhou *et al.*, 2011), PhiSpy (Akhter *et al.*, 2012) and VirSorter (Roux, Enault, *et al.*, 2015). The Venn diagram presents the number of genes that were predicted to be part of a prophage in a bacterial genome.

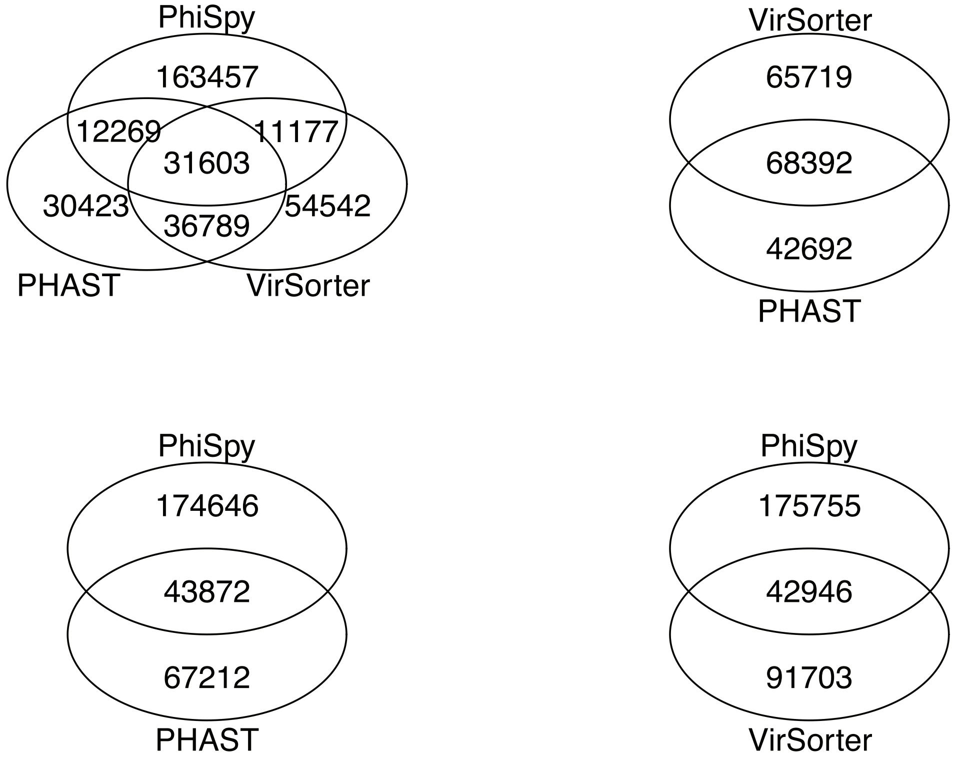

Figure S2. Cumulative distribution function of (A) the number of autologs per self-donor edge and (B) the number of phage nodes that are connected to self-donor nodes.

A

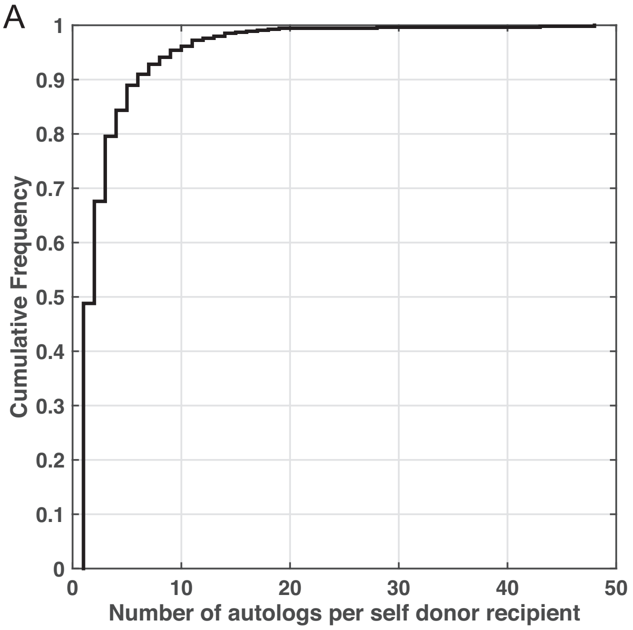

B

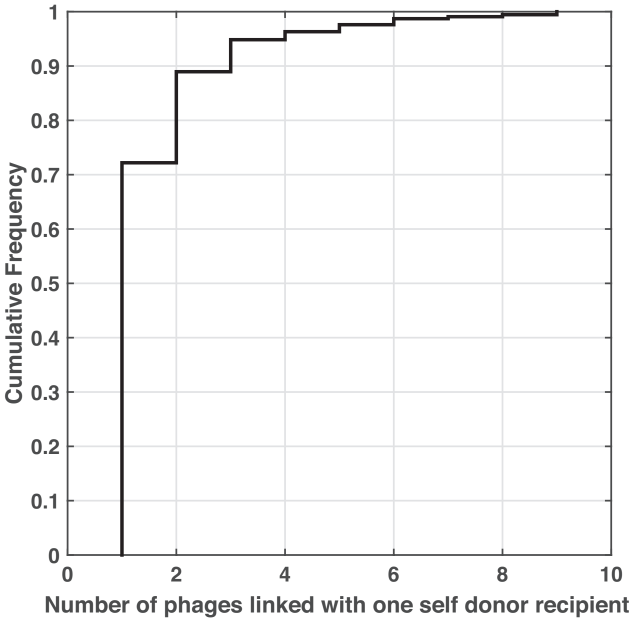

Figure S3. Transduction events. Detailed examples of phage entities from the dLGT network (A) Each phage entity (gray box) encompasses a single prophage or several orthologous prophages. Phage genes are shaded white and bacterial genes are shaded green when a donor could be inferred, or gray when a donor could not be inferred. Green numbers are dLGT node identifiers (see supplementary table 2), black numbers are prophage GI and region number as recorded in the PHAST DB. (B) Corresponding dLGT network views with donor and recipient bacterial genomes. Node color correspond to taxonomical group as described in Figure 1A. Edge color represents edge weight (color bar at bottom).

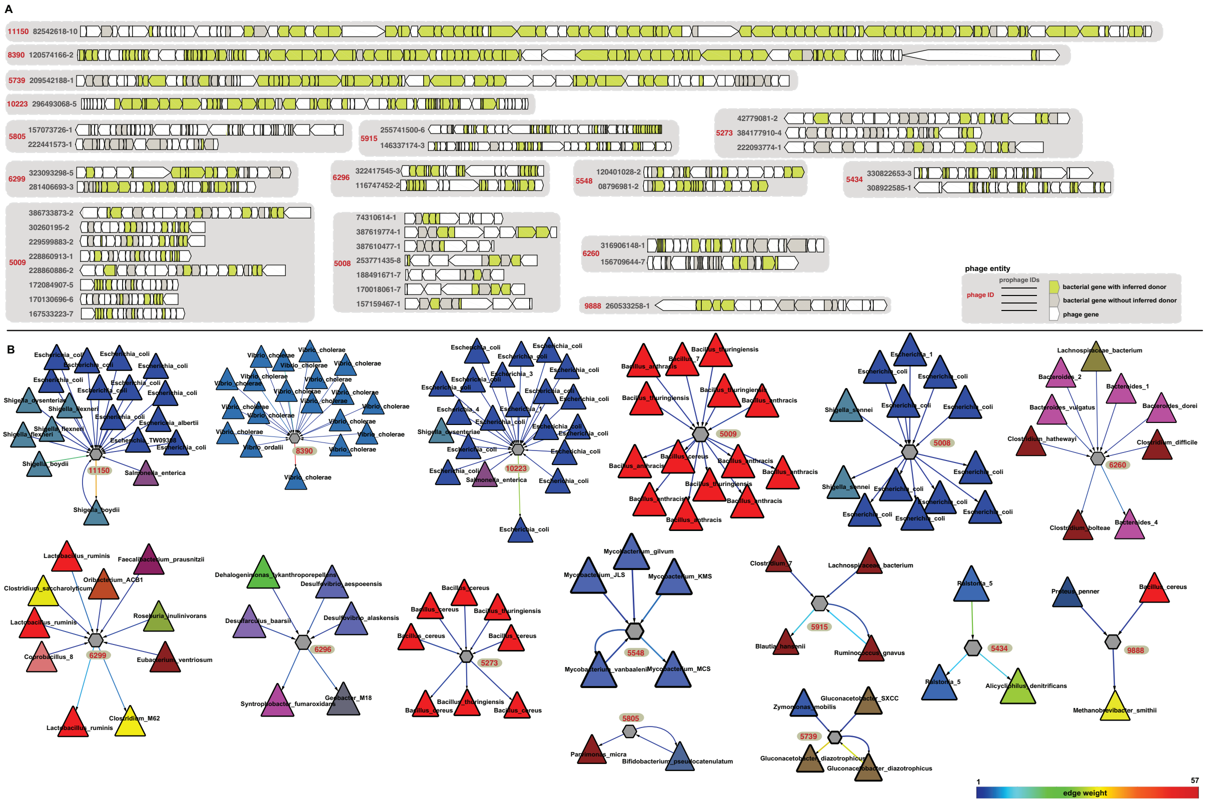

Figure S4. Edge weight distribution. Cumulative distribution function of edge weight in thenetwork.

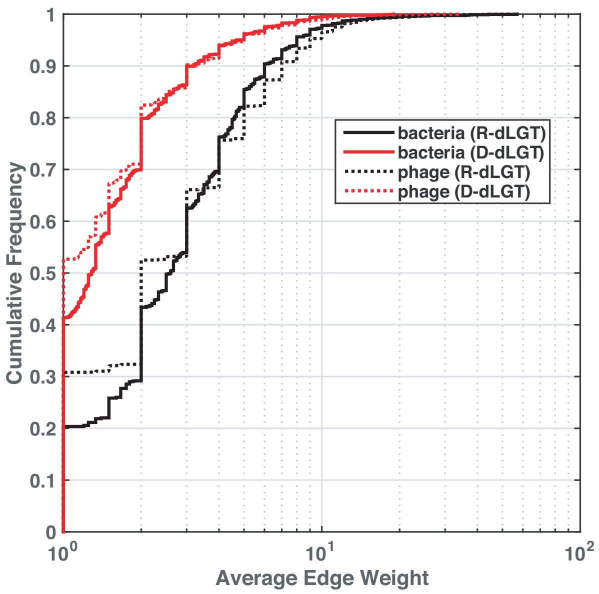

Figure S5. Donor - recipient genome similarity measurements (B) for a subset of prophages that were detected also by PhiSpy (Akhter *et al.*, 2012) or VirSorter (Roux, Enault, *et al.*, 2015) (termed here "subnetwork").

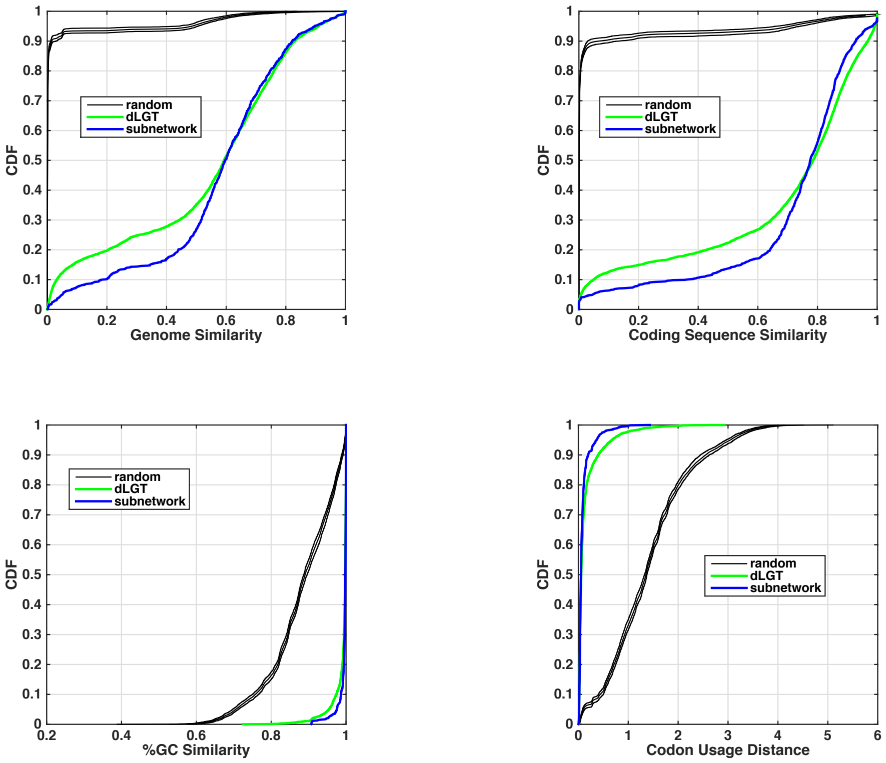

Figure S6. Heat map of connected donor and recipient species within the same or between different habitats. The numbers within each cell, correspond to the number of connected donors and recipients from a particular habitat. Cells coloured in red represent connected habitats in the dLGT network that show a higher frequency than expected, blue coloured cells correspond to lower frequency than expected and white cells are not significantly different than expected. Gray shaded box represents the distribution of the 3,982 bacterial species into a putative habitat. The values are sorted by size from top to down in a descend order.

Donor habitat \ Recipient habitat	Human	Host	Endosymbiont	Wastewater	Animal	Extreme Environments	Fresh water	Marine Environment	Not Annotated	Soil and Sediment	Plant
Human	1385	775	4	38	97	7	40	20	98	59	26
Host	858	1338	5	32	100	7	33	16	126	129	44
Endosymbiont	0	2	3	0	0	1	0	0	0	0	0
Wastewater	32	37	0	23	6	0	5	2	6	11	1
Animal	110	121	0	5	68	0	7	5	20	22	11
Extreme Environments	6	4	0	0	1	15	6	5	8	9	5
Fresh water	33	36	0	4	8	1	30	13	13	14	4
Marine Environment	14	13	0	5	3	4	9	38	10	11	0
Not Annotated	123	161	2	7	30	6	21	23	128	86	34
Soil and Sediment	64	123	0	12	26	5	18	20	55	266	51
Plant	14	46	0	3	6	5	5	0	29	41	50

Habitat	Number of species
Human	1120
Host	749
Not Annotated	644
Soil and Sediment	497
Marine Environment	256
Animal	226
Fresh water	180
Plant	130
Extreme Environments	112
Wastewater	55
Endosymbiont	13

Figure S7. Histogram of the relative proportion of acquired genes (LGTs) that were assigned to a COG category: information storage and processing (red), cellular processes and signaling (blue) and metabolism (green). Arrows indicate if the category is significantly overrepresented (→) or underrepresented (←) in the dLGT network (light colors) in comparison to the set of all bacterial genes (dark colors).

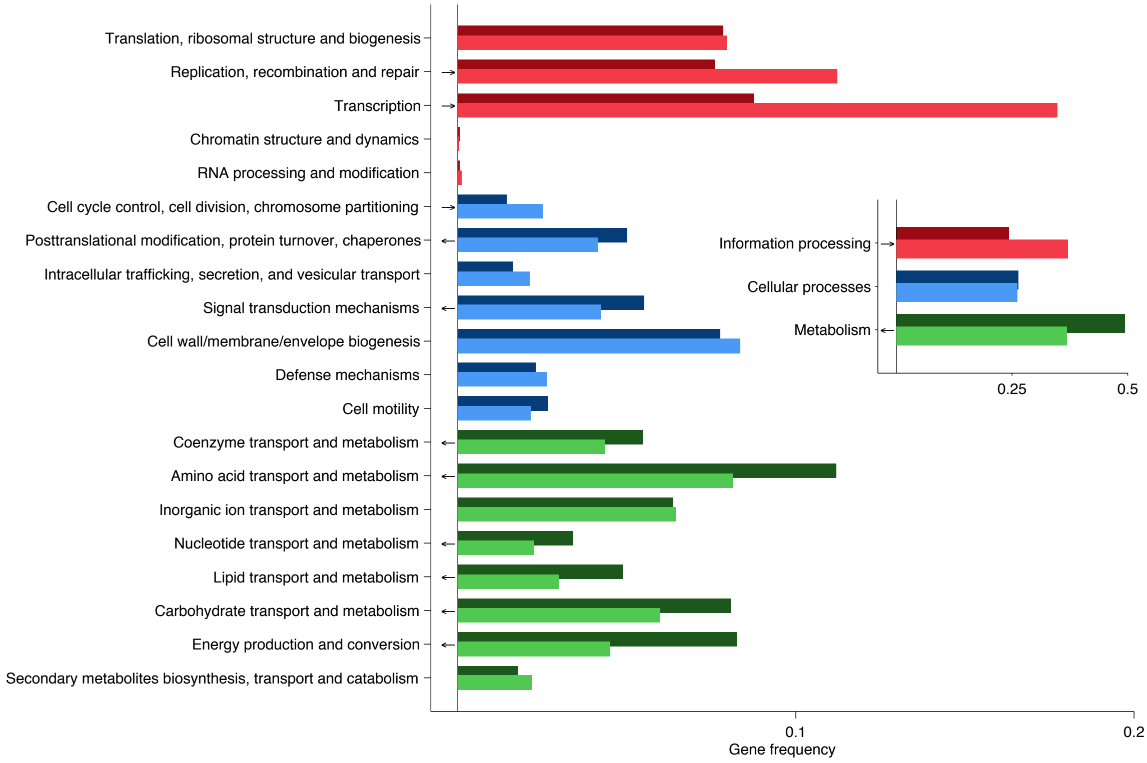

Table S1. dLGT-network analysis steps. The network reconstruction procedure includes two main task areas. The first area contain the orthologous prophage definition which defines the phage node in the dLGT network. Second area contains the dLGT network reconstruction, which describes the inference of donor-

	Step	Analysis	Input	Process and thresholds	Output
Orthologous prophage definition (phage entity)	1	Retrieve prophage annotations	PHAST data (version: 10/2012) download: 8,540 Accsessions (Chromosome/ plasmids/contigs)	Retain only genomes that include >=1 annotated prophage	4,473 accessions (Chromosome/ plasmids/contigs)
	2	Extract prophage annotations from PHAST DB	4,473 Accessions (Chromosome/ plasmids/contigs)	Retrieve all complete and incomplete prophage regions	14,920 prophage annotations
	3	Identification of orthologous genes in all prophages	14,920 prophages containing 535,630 genes	Reciprocal best BLAST hit procedure using a threshold of E-value <1x1e-10	496,710 genes with at least one hit
	4	Calculate global pairwise identity for reciprocal best BLAST pairs	497,610 genes	Pairwise alignment using neelde (EMBOSS package).Retain only pairs having >=95% amino acid identity	309,829 genes (3,389,249 pairs) in 12,087 prophages
	5	Calculate orthologus groups of prophage genes (MCL)	309,829 genes	inflation value 2.0	61,447 orthologous gene clusters
	6	Create a matrix for all 14,920 prophages and calculate the number of Shared Orthologus Gene Clusters (SOG matrix)	14,920 prophages containing 61,447 orthologus gene familes		SOG matrix of 14,920x14,920
	7	Define orthologous prophages from similarity by jaccard	SOG matrix of 14,920x14,920	>=0.7 jaccard index	6,494 prophages clustered into 2,397 phage entities
dLGT network reconstruction	1	Match PHAST genomes to NCBI GenBank genomes	3,982 full and draft	identical to accsession from PHAST	2,611 full+draft genomes
	2	Extract recipient gene sequence from PHAST prophage	2,611 genomes + 9,468 prophages	annotated as bacterial origin	89,234 genes in 9,201 prophages
	3	Find all homologous sequences for the recipient sequence (BLAST search against the 3,982 bacterial genomes)	89,234 genes	E-value <1x1e-10	75,172 querry genes + 3,908,830 hits genes
	4	Calculate global pairwise identity (EMBOSS needle)	75,172 querry genes + 3,908,830 hits genes	>=90% amino acid identity	715,440 pairs (42,760 querry genes + 252,159 hits = 271,270 unique genes)
	5	Calculate orthologus groups of the homologous genes (MCL)	271,270 genes	inflation value 2.0	20,904 orthologus gene clusters
	6	Phylogenetic gene tree calculation for each orthologous cluster (PhyML)	12,611 clusters with size >=3		12,611 trees
	7	Tree topology test for ambiguous topology (CONSEL)	2,205 trees	AU test p >=0.05	829 trees
	8	Reconstruct donor - recipient links (OTU-to-OTU)	12,611 trees + 8,293 paired genes (custer size of 2)	donors and recipients are sister taxa	2,573 bacteria nodes + 4,650 phage nodes connected by 15,298 edges comprising 17,158 genes
		Reconstruct donor - recipient links (OTU-to-HTU)	1,177 trees	donor is a taxonomic unit and recipient is a sister clade	1,018 bacteria nodes + 486 phage nodes connected by 4,258 edges comprising 2,488 genes
		Reconstruct donor - recipient links (HTU-to-OTU)	2,877 trees	donor is a sister clade and recipient is a taxonomic unit	2,092 bacteria nodes + 756 phage nodes connected by 13,498 edges comprising 4,140 genes
		Reconstruct donor - recipient links (HTU-to-HTU)	494 trees	donor and recipient are sister clades	1,156 bacteria nodes + 304 phage nodes connected by 4,337 edges comprising 1,748 genes
	9	Construct the bipartite dLGT network (OTU-to-OTU)	2,573 bacteria nodes + 4,650 phage nodes + 15,298 edges	connect bacterial donor node to phage node (D-dLGT) and the phage node to a bacterial recipient node (R-dLGT). Edge weights correspond to number of transferred genes in D-dLGT and R-dLGT.	(2,276 donor nodes + 10,216 edges (D-dLGT)) connected to 4,650 phage nodes connected to (1,916 recipient nodes + 5,082 edges (R-dLGT))
		Construct the bipartite dLGT network (OTU-to-HTU)	1,018 bacteria nodes + 486 phage nodes + 2,488 edges	connect bacterial donor node to phage node (D-dLGT) and the phage node to a bacterial recipient node (R-dLGT). Edge weights correspond to number of transferred genes in D-dLGT and 1/n in R-dLGT	516 donor nodes + 983 edges (D-dLGT)) connected to 486 phage nodes connected to (765 recipient nodes + 1,505 edges (R-dLGT))
		Construct the bipartite dLGT network (HTU-to-OTU)	2,092 bacteria nodes + 756 phage nodes + 13,498 edges	connect bacterial donor node to phage node (D-dLGT) and the phage node to a bacterial recipient node (R-dLGT). Edge weights correspond to 1/n in D-dLGT and number of transferred genes in R-dLGT	(1,981 donor nodes + 11,922 edges (D-dLGT)) connected to 756 phage nodes connected to (1,050 recipient nodes + 1,576 edges (R-dLGT))
		Construct the bipartite dLGT network (HTU-to-HTU)	1,156 bacteria nodes + 303 phage nodes + 4,337 edges	connect bacterial donor node to phage node (D-dLGT) and the phage node to a bacterial recipient node (R-dLGT). Edge weights correspond to 1/n in D-dLGT x 1/n in R-dLGT.	(994 donor nodes + 3,445 edges (D-dLGT)) connected to 303 phage nodes connected to 532 recipient nodes + 892 edges (R-dLGT))

Table S2. Network statistics.

		dLGT	R-dLGT	D-dLGT
Number of nodes	Bacteria	2,573	1,916	2,276
	Phage	4,650	4,650	4,650
	Total	7,223	6,566	6,926
Number of edges		15,298	5,082	10,216
Total edge weight		17,158	17,158	17,158
Node degree (median; maximum)	Bacteria	2;117	2;28	3;117
	Phage	1;20	1;8	1;20
Edge weight (median; minimum-maximum)		1;1-57	2; 1-57	1;1-36
Number of connected components		327	1.539	529
Largest component size	Bacteria	1,538	17	1,158
	Phage	3,444	145	3,008
	Total	4,982	162	4,166

Table S3. ICTV annotation. ICTV annotation are derived from the majority vote of ICTV labels of prophage genes

dLGT_phageID	ICTV Phage taxa
5001	Myoviridae
5001	Siphoviridae
5002	Siphoviridae
5004	Myoviridae
5006	Myoviridae
5006	Plasmaviridae
5006	Siphoviridae
5007	Myoviridae
5007	Podoviridae
5007	Siphoviridae
5008	Unknown
5008	Podoviridae
5009	Myoviridae
5009	Siphoviridae
5011	Siphoviridae
5012	Unknown
5012	Siphoviridae
5013	Myoviridae
5013	Unknown
5014	Unknown
5015	Siphoviridae
5016	Myoviridae
5016	Siphoviridae
5017	Siphoviridae
5018	Inoviridae
5018	Myoviridae
5019	Myoviridae
5020	Myoviridae
5021	Podoviridae
5022	Myoviridae
5023	Myoviridae
5023	Siphoviridae
5024	Unknown
5024	Siphoviridae
5025	Myoviridae
5025	Siphoviridae
5026	Myoviridae
5026	Unknown
5027	Siphoviridae
5028	Myoviridae

dLGT_phageID	ICTV Phage taxa
5028	Siphoviridae
5030	Myoviridae
5030	Unknown
5030	Siphoviridae
5031	Myoviridae
5031	Siphoviridae
5032	Siphoviridae
5033	Siphoviridae
5035	Siphoviridae
5036	Unknown
5036	Podoviridae
5036	Siphoviridae
5037	Myoviridae
5037	Siphoviridae
5038	Myoviridae
5038	Siphoviridae
5039	Myoviridae
5039	Podoviridae
5039	Siphoviridae
5040	Siphoviridae
5041	Myoviridae
5042	Myoviridae
5042	Siphoviridae
5045	Myoviridae
5045	Unknown
5045	Siphoviridae
5046	Siphoviridae
5047	Unknown
5048	Siphoviridae
5049	Myoviridae
5049	Unknown
5049	Siphoviridae
5050	Siphoviridae
5051	Myoviridae
5052	Siphoviridae
5054	Myoviridae
5054	Podoviridae
5054	Siphoviridae
5055	Unknown
5055	Siphoviridae
5057	Siphoviridae
5058	Unknown
5059	Myoviridae
5060	Unknown
5060	Siphoviridae

dLGT_phageID	ICTV Phage taxa
5061	Myoviridae
5061	Siphoviridae
5062	Siphoviridae
5063	Siphoviridae
5064	Siphoviridae
5065	Unknown
5065	Siphoviridae
5066	Siphoviridae
5067	Podoviridae
5068	Siphoviridae
5069	Siphoviridae
5070	Siphoviridae
5071	Myoviridae
5071	Siphoviridae
5072	Siphoviridae
5073	Myoviridae
5074	Myoviridae
5075	Myoviridae
5075	Podoviridae
5076	Siphoviridae
5077	Myoviridae
5077	Siphoviridae
5078	Unknown
5078	Siphoviridae
5079	Unknown
5079	Siphoviridae
5081	Unknown
5081	Siphoviridae
5082	Myoviridae
5082	Podoviridae
5082	Siphoviridae
5083	Unknown
5083	Siphoviridae
5084	Unknown
5085	Siphoviridae
5086	Podoviridae
5087	Myoviridae
5087	Siphoviridae
5088	Myoviridae
5089	Podoviridae
5090	Podoviridae
5091	Myoviridae
5091	Siphoviridae
5092	Siphoviridae
5093	Myoviridae

dLGT_phageID	ICTV Phage taxa
5093	Podoviridae
5096	Myoviridae
5097	Unknown
5099	Unknown
5100	Myoviridae
5101	Myoviridae
5101	Siphoviridae
5102	Podoviridae
5102	Siphoviridae
5103	Myoviridae
5104	Myoviridae
5104	Unknown
5105	Unknown
5105	Siphoviridae
5106	Myoviridae
5106	Siphoviridae
5107	Siphoviridae
5108	Siphoviridae
5109	Myoviridae
5109	Siphoviridae
5110	Myoviridae
5111	Unknown
5111	Siphoviridae
5112	Siphoviridae
5113	Siphoviridae
5114	Unknown
5114	Podoviridae
5114	Siphoviridae
5115	Myoviridae
5115	Siphoviridae
5116	Unknown
5116	Podoviridae
5117	Myoviridae
5117	Podoviridae
5118	Myoviridae
5119	Siphoviridae
5121	Siphoviridae
5122	Siphoviridae
5123	Unknown
5124	Siphoviridae
5125	Podoviridae
5125	Siphoviridae
5126	Myoviridae
5128	Podoviridae
5128	Siphoviridae

dLGT_phageID	ICTV Phage taxa
5130	Siphoviridae
5132	Podoviridae
5132	Siphoviridae
5133	Unknown
5134	Myoviridae
5134	Siphoviridae
5135	Unknown
5135	Podoviridae
5136	Unknown
5136	Podoviridae
5137	Siphoviridae
5138	Myoviridae
5139	Siphoviridae
5140	Myoviridae
5141	Siphoviridae
5143	Siphoviridae
5144	Myoviridae
5145	Myoviridae
5146	Siphoviridae
5147	Unknown
5148	Siphoviridae
5149	Siphoviridae
5151	Siphoviridae
5152	Myoviridae
5153	Myoviridae
5154	Myoviridae
5155	Podoviridae
5157	Myoviridae
5157	Siphoviridae
5158	Myoviridae
5158	Siphoviridae
5159	Siphoviridae
5160	Siphoviridae
5161	Siphoviridae
5162	Siphoviridae
5163	Siphoviridae
5164	Podoviridae
5164	Siphoviridae
5166	Myoviridae
5167	Myoviridae
5168	Siphoviridae
5171	Siphoviridae
5172	Siphoviridae
5173	Siphoviridae
5174	Unknown

dLGT_phageID	ICTV Phage taxa
5175	Myoviridae
5178	Siphoviridae
5179	Siphoviridae
5180	Siphoviridae
5181	Siphoviridae
5182	Unknown
5183	Siphoviridae
5185	Podoviridae
5186	Siphoviridae
5187	Siphoviridae
5188	Siphoviridae
5189	Podoviridae
5189	Siphoviridae
5190	Myoviridae
5190	Unknown
5190	Siphoviridae
5191	Unknown
5192	Siphoviridae
5194	Myoviridae
5194	Unknown
5196	Myoviridae
5196	Siphoviridae
5197	Siphoviridae
5198	Siphoviridae
5199	Siphoviridae
5200	Siphoviridae
5201	Myoviridae
5204	Siphoviridae
5205	Siphoviridae
5206	Myoviridae
5206	Siphoviridae
5207	Siphoviridae
5208	Myoviridae
5209	Siphoviridae
5210	Myoviridae
5211	Unknown
5211	Siphoviridae
5212	Siphoviridae
5213	Siphoviridae
5214	Myoviridae
5214	Siphoviridae
5216	Myoviridae
5216	Podoviridae
5216	Siphoviridae
5217	Siphoviridae

dLGT_phageID	ICTV Phage taxa
5218	Myoviridae
5218	Unknown
5220	Unknown
5221	Siphoviridae
5222	Unknown
5223	Siphoviridae
5224	Myoviridae
5225	Siphoviridae
5226	Unknown
5227	Unknown
5227	Siphoviridae
5228	Unknown
5230	Siphoviridae
5231	Myoviridae
5232	Myoviridae
5234	Myoviridae
5234	Siphoviridae
5235	Siphoviridae
5236	Myoviridae
5236	Siphoviridae
5237	Siphoviridae
5238	Myoviridae
5239	Siphoviridae
5240	Myoviridae
5241	Siphoviridae
5244	Unknown
5247	Plasmaviridae
5249	Siphoviridae
5251	Siphoviridae
5254	Siphoviridae
5257	Siphoviridae
5258	Siphoviridae
5263	Myoviridae
5263	Unknown
5263	Siphoviridae
5264	Siphoviridae
5265	Siphoviridae
5267	Siphoviridae
5268	Siphoviridae
5269	Myoviridae
5270	Myoviridae
5272	Siphoviridae
5273	Myoviridae
5273	Siphoviridae
5274	Siphoviridae

dLGT_phageID	ICTV Phage taxa
5275	Siphoviridae
5276	Siphoviridae
5277	Myoviridae
5278	Podoviridae
5279	Myoviridae
5279	Unknown
5280	Myoviridae
5280	Unknown
5281	Unknown
5282	Siphoviridae
5283	Siphoviridae
5284	Siphoviridae
5285	Siphoviridae
5286	Siphoviridae
5287	Myoviridae
5288	Siphoviridae
5290	Myoviridae
5291	Siphoviridae
5292	Siphoviridae
5293	Podoviridae
5293	Siphoviridae
5294	Myoviridae
5295	Myoviridae
5295	Siphoviridae
5297	Siphoviridae
5299	Myoviridae
5300	Myoviridae
5301	Myoviridae
5301	Siphoviridae
5302	Unknown
5303	Siphoviridae
5304	Unknown
5305	Siphoviridae
5306	Podoviridae
5306	Siphoviridae
5308	Myoviridae
5308	Siphoviridae
5310	Siphoviridae
5311	Unknown
5312	Siphoviridae
5313	Myoviridae
5313	Siphoviridae
5314	Siphoviridae
5315	Unknown
5316	Unknown

dLGT_phageID	ICTV Phage taxa
5317	Myoviridae
5318	Siphoviridae
5319	Siphoviridae
5322	Myoviridae
5323	Unknown
5324	Myoviridae
5325	Siphoviridae
5326	Myoviridae
5326	Siphoviridae
5327	Myoviridae
5327	Siphoviridae
5328	Unknown
5328	Siphoviridae
5329	Unknown
5330	Podoviridae
5331	Siphoviridae
5332	Myoviridae
5333	Unknown
5334	Myoviridae
5335	Siphoviridae
5336	Siphoviridae
5337	Myoviridae
5337	Siphoviridae
5338	Myoviridae
5338	Unknown
5338	Siphoviridae
5339	Myoviridae
5339	Podoviridae
5339	Siphoviridae
5340	Myoviridae
5341	Siphoviridae
5342	Myoviridae
5343	Siphoviridae
5344	Siphoviridae
5345	Siphoviridae
5346	Myoviridae
5347	Myoviridae
5348	Siphoviridae
5349	Siphoviridae
5351	Siphoviridae
5352	Siphoviridae
5354	Siphoviridae
5355	Myoviridae
5356	Siphoviridae
5357	Myoviridae

dLGT_phageID	ICTV Phage taxa
5357	Unknown
5358	Siphoviridae
5359	Unknown
5361	Myoviridae
5361	Siphoviridae
5362	Siphoviridae
5366	Myoviridae
5368	Myoviridae
5369	Myoviridae
5370	Myoviridae
5370	Siphoviridae
5374	Myoviridae
5374	Siphoviridae
5375	Myoviridae
5375	Siphoviridae
5376	Siphoviridae
5377	Siphoviridae
5378	Unknown
5378	Siphoviridae
5379	Myoviridae
5380	Siphoviridae
5381	Siphoviridae
5382	Siphoviridae
5386	Siphoviridae
5387	Siphoviridae
5388	Siphoviridae
5389	Unknown
5389	Siphoviridae
5391	Siphoviridae
5392	Unknown
5393	Siphoviridae
5394	Siphoviridae
5395	Unknown
5397	Myoviridae
5398	Unknown
5398	Siphoviridae
5399	Siphoviridae
5400	Myoviridae
5401	Myoviridae
5402	Podoviridae
5403	Myoviridae
5404	Siphoviridae
5405	Myoviridae
5407	Myoviridae
5408	Siphoviridae

dLGT_phageID	ICTV Phage taxa
5409	Myoviridae
5409	Siphoviridae
5411	Siphoviridae
5413	Siphoviridae
5414	Siphoviridae
5416	Podoviridae
5417	Myoviridae
5418	Siphoviridae
5422	Siphoviridae
5423	Siphoviridae
5424	Siphoviridae
5427	Siphoviridae
5429	Siphoviridae
5430	Myoviridae
5431	Siphoviridae
5433	Inoviridae
5434	Siphoviridae
5437	Siphoviridae
5439	Siphoviridae
5442	Siphoviridae
5443	Siphoviridae
5444	Myoviridae
5445	Podoviridae
5446	Siphoviridae
5448	Siphoviridae
5450	Unknown
5450	Siphoviridae
5451	Siphoviridae
5452	Siphoviridae
5456	Siphoviridae
5459	Siphoviridae
5464	Siphoviridae
5465	Unknown
5465	Siphoviridae
5466	Myoviridae
5467	Siphoviridae
5468	Myoviridae
5468	Siphoviridae
5469	Myoviridae
5470	Myoviridae
5470	Unknown
5473	Siphoviridae
5475	Myoviridae
5477	Siphoviridae
5478	Inoviridae

dLGT_phageID	ICTV Phage taxa
5479	Siphoviridae
5481	Siphoviridae
5482	Siphoviridae
5484	Siphoviridae
5485	Siphoviridae
5486	Myoviridae
5487	Siphoviridae
5488	Podoviridae
5489	Unknown
5490	Siphoviridae
5491	Siphoviridae
5493	Siphoviridae
5496	Myoviridae
5497	Myoviridae
5497	Siphoviridae
5498	Siphoviridae
5499	Siphoviridae
5500	Myoviridae
5502	Siphoviridae
5504	Unknown
5504	Siphoviridae
5505	Unknown
5506	Siphoviridae
5507	Myoviridae
5508	Siphoviridae
5508	Tectiviridae
5509	Unknown
5509	Siphoviridae
5510	Podoviridae
5510	Siphoviridae
5511	Myoviridae
5512	Unknown
5513	Myoviridae
5513	Siphoviridae
5515	Myoviridae
5516	Podoviridae
5517	Siphoviridae
5518	Unknown
5521	Siphoviridae
5523	Unknown
5524	Myoviridae
5526	Podoviridae
5529	Unknown
5530	Siphoviridae
5531	Myoviridae

dLGT_phageID	ICTV Phage taxa
5532	Siphoviridae
5534	Myoviridae
5536	Siphoviridae
5537	Myoviridae
5538	Siphoviridae
5540	Siphoviridae
5541	Myoviridae
5542	Siphoviridae
5543	Myoviridae
5543	Siphoviridae
5544	Siphoviridae
5546	Siphoviridae
5548	Unknown
5548	Siphoviridae
5549	Myoviridae
5550	Myoviridae
5551	Siphoviridae
5553	Podoviridae
5559	Siphoviridae
5560	Myoviridae
5561	Siphoviridae
5562	Siphoviridae
5564	Unknown
5565	Siphoviridae
5566	Unknown
5566	Podoviridae
5568	Podoviridae
5570	Myoviridae
5571	Siphoviridae
5572	Unknown
5573	Siphoviridae
5574	Myoviridae
5574	Siphoviridae
5576	Myoviridae
5577	Podoviridae
5578	Myoviridae
5579	Siphoviridae
5580	Myoviridae
5580	Podoviridae
5581	Siphoviridae
5582	Siphoviridae
5583	Myoviridae
5584	Podoviridae
5584	Siphoviridae
5585	Siphoviridae

dLGT_phageID	ICTV Phage taxa
5587	Myoviridae
5588	Siphoviridae
5589	Siphoviridae
5590	Myoviridae
5592	Podoviridae
5593	Myoviridae
5593	Siphoviridae
5594	Myoviridae
5595	Siphoviridae
5596	Unknown
5597	Myoviridae
5598	Siphoviridae
5599	Siphoviridae
5601	Siphoviridae
5602	Siphoviridae
5603	Siphoviridae
5605	Siphoviridae
5606	Siphoviridae
5607	Siphoviridae
5608	Myoviridae
5610	Siphoviridae
5611	Unknown
5612	Siphoviridae
5614	Siphoviridae
5615	Siphoviridae
5618	Siphoviridae
5620	Siphoviridae
5621	Siphoviridae
5622	Siphoviridae
5623	Unknown
5623	Siphoviridae
5624	Unknown
5624	Siphoviridae
5625	Siphoviridae
5627	Unknown
5628	Unknown
5629	Siphoviridae
5630	Myoviridae
5630	Siphoviridae
5631	Inoviridae
5631	Podoviridae
5636	Siphoviridae
5640	Siphoviridae
5641	Myoviridae
5642	Siphoviridae

dLGT_phageID	ICTV Phage taxa
5643	Myoviridae
5643	Siphoviridae
5644	Unknown
5645	Siphoviridae
5646	Siphoviridae
5647	Siphoviridae
5648	Siphoviridae
5649	Myoviridae
5650	Podoviridae
5651	Myoviridae
5652	Siphoviridae
5653	Myoviridae
5654	Myoviridae
5655	Myoviridae
5656	Siphoviridae
5657	Siphoviridae
5659	Siphoviridae
5660	Siphoviridae
5662	Siphoviridae
5663	Siphoviridae
5664	Myoviridae
5664	Podoviridae
5665	Myoviridae
5665	Siphoviridae
5666	Myoviridae
5668	Podoviridae
5669	Inoviridae
5670	Podoviridae
5671	Siphoviridae
5675	Siphoviridae
5676	Myoviridae
5677	Siphoviridae
5678	Siphoviridae
5680	Unknown
5681	Podoviridae
5681	Siphoviridae
5684	Podoviridae
5686	Myoviridae
5688	Siphoviridae
5689	Myoviridae
5690	Siphoviridae
5691	Siphoviridae
5692	Siphoviridae
5693	Siphoviridae
5694	Myoviridae

dLGT_phageID	ICTV Phage taxa
5695	Myoviridae
5696	Myoviridae
5696	Siphoviridae
5697	Siphoviridae
5698	Podoviridae
5702	Siphoviridae
5706	Myoviridae
5707	Siphoviridae
5712	Podoviridae
5712	Siphoviridae
5713	Unknown
5714	Myoviridae
5715	Siphoviridae
5716	Podoviridae
5718	Myoviridae
5719	Podoviridae
5720	Myoviridae
5721	Podoviridae
5722	Siphoviridae
5723	Myoviridae
5724	Inoviridae
5724	Myoviridae
5725	Myoviridae
5726	Unknown
5726	Siphoviridae
5727	Siphoviridae
5733	Myoviridae
5734	Siphoviridae
5735	Unknown
5736	Siphoviridae
5738	Myoviridae
5739	Unknown
5739	Siphoviridae
5741	Myoviridae
5742	Unknown
5742	Siphoviridae
5743	Siphoviridae
5744	Myoviridae
5745	Siphoviridae
5746	Podoviridae
5749	Podoviridae
5751	Myoviridae
5752	Myoviridae
5753	Siphoviridae
5754	Siphoviridae

dLGT_phageID	ICTV Phage taxa
5755	Siphoviridae
5756	Siphoviridae
5757	Siphoviridae
5758	Podoviridae
5759	Podoviridae
5760	Myoviridae
5761	Siphoviridae
5762	Siphoviridae
5763	Siphoviridae
5764	Siphoviridae
5765	Myoviridae
5765	Siphoviridae
5766	Myoviridae
5766	Podoviridae
5767	Myoviridae
5768	Podoviridae
5768	Siphoviridae
5769	Siphoviridae
5770	Myoviridae
5771	Siphoviridae
5772	Siphoviridae
5773	Myoviridae
5775	Myoviridae
5775	Siphoviridae
5777	Myoviridae
5778	Siphoviridae
5779	Myoviridae
5780	Siphoviridae
5781	Siphoviridae
5783	Myoviridae
5784	Siphoviridae
5785	Siphoviridae
5786	Unknown
5787	Siphoviridae
5788	Myoviridae
5788	Podoviridae
5789	Podoviridae
5789	Siphoviridae
5790	Siphoviridae
5791	Siphoviridae
5792	Myoviridae
5792	Siphoviridae
5793	Podoviridae
5793	Siphoviridae
5794	Myoviridae

dLGT_phageID	ICTV Phage taxa
5794	Podoviridae
5795	Myoviridae
5798	Myoviridae
5799	Siphoviridae
5801	Podoviridae
5802	Myoviridae
5803	Siphoviridae
5805	Siphoviridae
5806	Siphoviridae
5807	Corticoviridae
5808	Myoviridae
5808	Siphoviridae
5809	Siphoviridae
5810	Siphoviridae
5811	Siphoviridae
5812	Myoviridae
5813	Siphoviridae
5814	Myoviridae
5817	Myoviridae
5818	Siphoviridae
5819	Plasmaviridae
5820	Siphoviridae
5821	Siphoviridae
5824	Siphoviridae
5825	Myoviridae
5827	Siphoviridae
5828	Siphoviridae
5829	Podoviridae
5830	Siphoviridae
5831	Siphoviridae
5832	Siphoviridae
5834	Myoviridae
5834	Siphoviridae
5835	Siphoviridae
5836	Siphoviridae
5837	Siphoviridae
5838	Unknown
5839	Siphoviridae
5840	Siphoviridae
5841	Siphoviridae
5842	Myoviridae
5843	Unknown
5844	Myoviridae
5845	Siphoviridae
5846	Myoviridae

dLGT_phageID	ICTV Phage taxa
5846	Plasmaviridae
5847	Unknown
5847	Siphoviridae
5848	Unknown
5848	Siphoviridae
5849	Unknown
5850	Siphoviridae
5851	Unknown
5852	Unknown
5853	Myoviridae
5854	Siphoviridae
5855	Siphoviridae
5857	Siphoviridae
5858	Siphoviridae
5861	Siphoviridae
5862	Unknown
5863	Siphoviridae
5864	Unknown
5864	Siphoviridae
5865	Siphoviridae
5866	Unknown
5867	Podoviridae
5868	Myoviridae
5872	Podoviridae
5874	Podoviridae
5874	Siphoviridae
5875	Podoviridae
5876	Siphoviridae
5877	Unknown
5878	Myoviridae
5882	Siphoviridae
5883	Unknown
5883	Podoviridae
5884	Podoviridae
5884	Siphoviridae
5885	Siphoviridae
5886	Siphoviridae
5887	Myoviridae
5888	Siphoviridae
5889	Unknown
5890	Siphoviridae
5893	Siphoviridae
5895	Myoviridae
5896	Siphoviridae
5898	Myoviridae

dLGT_phageID	ICTV Phage taxa
5900	Myoviridae
5902	Siphoviridae
5903	Siphoviridae
5904	Siphoviridae
5905	Unknown
5906	Unknown
5907	Siphoviridae
5908	Siphoviridae
5909	Podoviridae
5911	Siphoviridae
5912	Myoviridae
5913	Siphoviridae
5915	Inoviridae
5915	Siphoviridae
5916	Unknown
5917	Siphoviridae
5918	Unknown
5919	Siphoviridae
5921	Unknown
5922	Siphoviridae
5923	Siphoviridae
5925	Siphoviridae
5928	Siphoviridae
5930	Siphoviridae
5931	Siphoviridae
5932	Myoviridae
5932	Unknown
5933	Myoviridae
5934	Myoviridae
5935	Podoviridae
5935	Siphoviridae
5936	Siphoviridae
5937	Myoviridae
5938	Myoviridae
5938	Siphoviridae
5939	Siphoviridae
5940	Myoviridae
5941	Myoviridae
5942	Unknown
5943	Siphoviridae
5944	Podoviridae
5946	Siphoviridae
5947	Siphoviridae
5948	Siphoviridae
5949	Siphoviridae

dLGT_phageID	ICTV Phage taxa
5950	Myoviridae
5951	Siphoviridae
5952	Siphoviridae
5953	Siphoviridae
5954	Unknown
5955	Myoviridae
5956	Siphoviridae
5957	Siphoviridae
5958	Siphoviridae
5959	Podoviridae
5960	Podoviridae
5962	Inoviridae
5964	Myoviridae
5965	Myoviridae
5966	Myoviridae
5967	Siphoviridae
5968	Siphoviridae
5969	Unknown
5970	Unknown
5971	Siphoviridae
5972	Siphoviridae
5973	Siphoviridae
5974	Unknown
5975	Siphoviridae
5978	Siphoviridae
5979	Siphoviridae
5980	Siphoviridae
5981	Siphoviridae
5982	Siphoviridae
5984	Siphoviridae
5985	Myoviridae
5986	Unknown
5987	Unknown
5988	Siphoviridae
5989	Myoviridae
5990	Siphoviridae
5991	Myoviridae
5991	Siphoviridae
5992	Myoviridae
5992	Siphoviridae
5993	Unknown
5994	Myoviridae
5995	Unknown
5996	Myoviridae
5997	Siphoviridae

dLGT_phageID	ICTV Phage taxa
6049	Podoviridae
6049	Siphoviridae
6050	Unknown
6051	Unknown
6052	Siphoviridae
6055	Myoviridae
6056	Myoviridae
6056	Siphoviridae
6057	Podoviridae
6058	Podoviridae
6059	Siphoviridae
6060	Unknown
6061	Myoviridae
6062	Siphoviridae
6063	Siphoviridae
6064	Siphoviridae
6065	Unknown
6066	Siphoviridae
6067	Siphoviridae
6068	Unknown
6070	Myoviridae
6071	Myoviridae
6074	Siphoviridae
6078	Unknown
6079	Siphoviridae
6081	Myoviridae
6081	Unknown
6083	Siphoviridae
6084	Myoviridae
6084	Siphoviridae
6085	Siphoviridae
6086	Siphoviridae
6087	Myoviridae
6087	Unknown
6091	Myoviridae
6091	Siphoviridae
6092	Siphoviridae
6093	Siphoviridae
6094	Siphoviridae
6095	Siphoviridae
6097	Unknown
6097	Siphoviridae
6098	Siphoviridae
6099	Myoviridae
6099	Siphoviridae

dLGT_phageID	ICTV Phage taxa
5998	Siphoviridae
6000	Podoviridae
6001	Siphoviridae
6002	Myoviridae
6002	Siphoviridae
6003	Podoviridae
6004	Myoviridae
6006	Siphoviridae
6008	Unknown
6009	Siphoviridae
6010	Siphoviridae
6011	Siphoviridae
6012	Siphoviridae
6015	Siphoviridae
6016	Myoviridae
6017	Myoviridae
6018	Siphoviridae
6022	Myoviridae
6022	Siphoviridae
6023	Siphoviridae
6024	Siphoviridae
6026	Unknown
6027	Myoviridae
6028	Myoviridae
6029	Siphoviridae
6030	Siphoviridae
6031	Myoviridae
6033	Siphoviridae
6034	Unknown
6035	Myoviridae
6035	Unknown
6036	Unknown
6036	Siphoviridae
6037	Siphoviridae
6038	Siphoviridae
6039	Siphoviridae
6040	Siphoviridae
6041	Myoviridae
6042	Podoviridae
6043	Myoviridae
6043	Siphoviridae
6044	Myoviridae
6046	Myoviridae
6047	Unknown
6048	Myoviridae

dLGT_phageID	ICTV Phage taxa
6100	Myoviridae
6105	Siphoviridae
6106	Myoviridae
6109	Myoviridae
6109	Siphoviridae
6110	Siphoviridae
6111	Siphoviridae
6112	Podoviridae
6113	Myoviridae
6114	Siphoviridae
6115	Siphoviridae
6116	Myoviridae
6118	Myoviridae
6119	Inoviridae
6119	Siphoviridae
6120	Siphoviridae
6121	Unknown
6122	Myoviridae
6123	Siphoviridae
6124	Myoviridae
6125	Siphoviridae
6126	Myoviridae
6127	Siphoviridae
6128	Myoviridae
6129	Myoviridae
6130	Myoviridae
6131	Myoviridae
6132	Myoviridae
6133	Podoviridae
6134	Tectiviridae
6135	Siphoviridae
6136	Siphoviridae
6137	Siphoviridae
6138	Siphoviridae
6144	Siphoviridae
6146	Unknown
6147	Siphoviridae
6148	Myoviridae
6150	Siphoviridae
6154	Myoviridae
6155	Podoviridae
6155	Siphoviridae
6157	Siphoviridae
6159	Podoviridae
6168	Myoviridae

dLGT_phageID	ICTV Phage taxa
6168	Siphoviridae
6169	Siphoviridae
6170	Siphoviridae
6171	Unknown
6172	Podoviridae
6173	Unknown
6174	Siphoviridae
6175	Siphoviridae
6177	Myoviridae
6178	Myoviridae
6179	Siphoviridae
6181	Unknown
6182	Myoviridae
6183	Siphoviridae
6184	Myoviridae
6187	Siphoviridae
6188	Myoviridae
6189	Podoviridae
6191	Unknown
6195	Siphoviridae
6196	Myoviridae
6196	Siphoviridae
6197	Siphoviridae
6200	Siphoviridae
6201	Siphoviridae
6203	Myoviridae
6203	Siphoviridae
6204	Unknown
6206	Myoviridae
6208	Myoviridae
6209	Myoviridae
6210	Myoviridae
6210	Siphoviridae
6211	Siphoviridae
6212	Siphoviridae
6213	Siphoviridae
6214	Siphoviridae
6215	Unknown
6216	Myoviridae
6217	Siphoviridae
6218	Siphoviridae
6220	Unknown
6221	Unknown
6222	Siphoviridae
6223	Siphoviridae

dLGT_phageID	ICTV Phage taxa
6224	Myoviridae
6225	Siphoviridae
6226	Myoviridae
6228	Siphoviridae
6229	Siphoviridae
6233	Siphoviridae
6236	Myoviridae
6242	Siphoviridae
6244	Siphoviridae
6247	Siphoviridae
6248	Siphoviridae
6249	Siphoviridae
6252	Siphoviridae
6253	Siphoviridae
6254	Myoviridae
6256	Unknown
6257	Siphoviridae
6259	Unknown
6260	Siphoviridae
6261	Unknown
6261	Siphoviridae
6262	Siphoviridae
6263	Siphoviridae
6265	Unknown
6267	Podoviridae
6267	Siphoviridae
6268	Siphoviridae
6269	Siphoviridae
6270	Siphoviridae
6274	Myoviridae
6276	Siphoviridae
6277	Siphoviridae
6278	Myoviridae
6279	Siphoviridae
6289	Myoviridae
6290	Siphoviridae
6292	Siphoviridae
6293	Myoviridae
6294	Myoviridae
6294	Siphoviridae
6295	Myoviridae
6295	Siphoviridae
6296	Myoviridae
6298	Siphoviridae
6299	Unknown

dLGT_phageID	ICTV Phage taxa
6308	Myoviridae
6309	Siphoviridae
6315	Podoviridae
6317	Siphoviridae
6318	Myoviridae
6319	Myoviridae
6321	Siphoviridae
6322	Siphoviridae
6326	Inoviridae
6327	Unknown
6327	Siphoviridae
6328	Siphoviridae
6330	Siphoviridae
6333	Siphoviridae
6334	Myoviridae
6334	Siphoviridae
6337	Unknown
6338	Unknown
6338	Siphoviridae
6340	Siphoviridae
6342	Podoviridae
6348	Unknown
6348	Siphoviridae
6357	Siphoviridae
6359	Siphoviridae
6361	Myoviridae
6362	Siphoviridae
6371	Siphoviridae
6378	Myoviridae
6380	Myoviridae
6381	Siphoviridae
6382	Podoviridae
6384	Siphoviridae
6386	Siphoviridae
6387	Myoviridae
6388	Podoviridae
6389	Podoviridae
6390	Myoviridae
6390	Siphoviridae
6399	Siphoviridae
6400	Myoviridae
6400	Siphoviridae
6401	Podoviridae
6401	Siphoviridae
6402	Siphoviridae

dLGT_phageID	ICTV Phage taxa
6403	Unknown
6403	Siphoviridae
6404	Siphoviridae
6406	Siphoviridae
6407	Siphoviridae
6408	Siphoviridae
6410	Siphoviridae
6411	Siphoviridae
6412	Siphoviridae
6413	Siphoviridae
6415	Siphoviridae
6417	Siphoviridae
6418	Siphoviridae
6422	Myoviridae
6423	Myoviridae
6424	Myoviridae
6425	Siphoviridae
6426	Myoviridae
6427	Myoviridae
6430	Myoviridae
6431	Siphoviridae
6432	Podoviridae
6432	Siphoviridae
6440	Myoviridae
6440	Podoviridae
6441	Myoviridae
6441	Siphoviridae
6442	Siphoviridae
6447	Myoviridae
6448	Myoviridae
6450	Myoviridae
6451	Siphoviridae
6460	Myoviridae
6463	Siphoviridae
6464	Podoviridae
6467	Siphoviridae
6471	Myoviridae
6472	Siphoviridae
6473	Myoviridae
6473	Siphoviridae
6474	Siphoviridae
6475	Siphoviridae
6476	Unknown
6477	Siphoviridae
6478	Unknown

dLGT_phageID	ICTV Phage taxa
6478	Siphoviridae
6480	Siphoviridae
6481	Myoviridae
6481	Siphoviridae
6482	Unknown
6483	Siphoviridae
6484	Siphoviridae
6485	Siphoviridae
6488	Siphoviridae
6489	Podoviridae
6489	Siphoviridae
6491	Siphoviridae
6492	Siphoviridae
6493	Siphoviridae
6494	Unknown
6494	Siphoviridae
6496	Siphoviridae
6497	Siphoviridae
6499	Myoviridae
6499	Siphoviridae
6504	Siphoviridae
6505	Myoviridae
6507	Siphoviridae
6509	Myoviridae
6510	Siphoviridae
6512	Podoviridae
6513	Siphoviridae
6514	Unknown
6517	Siphoviridae
6518	Siphoviridae
6520	Myoviridae
6524	Siphoviridae
6526	Unknown
6529	Siphoviridae
6532	Myoviridae
6539	Siphoviridae
6541	Myoviridae
6541	Podoviridae
6544	Siphoviridae
6545	Siphoviridae
6546	Myoviridae
6551	Siphoviridae
6552	Myoviridae
6553	Siphoviridae
6554	Siphoviridae

dLGT_phageID	ICTV Phage taxa
6555	Siphoviridae
6566	Unknown
6566	Siphoviridae
6569	Unknown
6575	Siphoviridae
6576	Myoviridae
6577	Myoviridae
6578	Unknown
6579	Podoviridae
6579	Siphoviridae
6582	Unknown
6585	Siphoviridae
6586	Siphoviridae
6588	Siphoviridae
6591	Siphoviridae
6593	Myoviridae
6595	Siphoviridae
6596	Podoviridae
6597	Siphoviridae
6599	Myoviridae
6603	Siphoviridae
6604	Myoviridae
6605	Siphoviridae
6606	Siphoviridae
6610	Unknown
6611	Unknown
6612	Podoviridae
6613	Unknown
6614	Siphoviridae
6616	Unknown
6617	Podoviridae
6618	Siphoviridae
6619	Siphoviridae
6620	Siphoviridae
6621	Podoviridae
6623	Unknown
6631	Myoviridae
6632	Myoviridae
6633	Siphoviridae
6637	Myoviridae
6637	Siphoviridae
6639	Inoviridae
6639	Siphoviridae
6642	Siphoviridae
6643	Unknown

dLGT_phageID	ICTV Phage taxa
6645	Siphoviridae
6646	Unknown
6647	Siphoviridae
6648	Siphoviridae
6649	Siphoviridae
6651	Unknown
6651	Siphoviridae
6652	Siphoviridae
6653	Siphoviridae
6654	Siphoviridae
6655	Siphoviridae
6656	Siphoviridae
6657	Siphoviridae
6658	Myoviridae
6659	Myoviridae
6660	Siphoviridae
6661	Siphoviridae
6662	Siphoviridae
6663	Siphoviridae
6664	Siphoviridae
6665	Myoviridae
6666	Siphoviridae
6667	Myoviridae
6668	Siphoviridae
6671	Siphoviridae
6673	Myoviridae
6674	Podoviridae
6675	Myoviridae
6676	Myoviridae
6677	Myoviridae
6678	Siphoviridae
6681	Unknown
6682	Podoviridae
6683	Siphoviridae
6684	Podoviridae
6686	Siphoviridae
6687	Siphoviridae
6688	Myoviridae
6689	Myoviridae
6693	Siphoviridae
6696	Myoviridae
6697	Siphoviridae
6698	Myoviridae
6699	Myoviridae
6700	Myoviridae

dLGT_phageID	ICTV Phage taxa
6701	Siphoviridae
6702	Siphoviridae
6703	Myoviridae
6704	Siphoviridae
6705	Siphoviridae
6707	Siphoviridae
6708	Siphoviridae
6709	Myoviridae
6710	Podoviridae
6711	Myoviridae
6712	Siphoviridae
6713	Siphoviridae
6714	Myoviridae
6716	Podoviridae
6717	Siphoviridae
6718	Siphoviridae
6720	Unknown
6725	Siphoviridae
6726	Myoviridae
6727	Siphoviridae
6730	Siphoviridae
6733	Siphoviridae
6734	Myoviridae
6735	Myoviridae
6737	Unknown
6740	Podoviridae
6741	Unknown
6742	Myoviridae
6743	Myoviridae
6750	Myoviridae
6751	Podoviridae
6758	Podoviridae
6760	Podoviridae
6761	Myoviridae
6762	Siphoviridae
6763	Siphoviridae
6764	Myoviridae
6765	Myoviridae
6766	Unknown
6769	Myoviridae
6770	Unknown
6771	Podoviridae
6772	Unknown
6773	Siphoviridae
6774	Myoviridae

dLGT_phageID	ICTV Phage taxa
6776	Myoviridae
6777	Myoviridae
6779	Siphoviridae
6780	Siphoviridae
6781	Siphoviridae
6783	Siphoviridae
6786	Siphoviridae
6787	Siphoviridae
6788	Siphoviridae
6789	Unknown
6791	Siphoviridae
6793	Podoviridae
6797	Siphoviridae
6798	Unknown
6799	Unknown
6800	Siphoviridae
6801	Siphoviridae
6802	Siphoviridae
6803	Unknown
6804	Unknown
6805	Siphoviridae
6806	Siphoviridae
6807	Podoviridae
6808	Unknown
6809	Siphoviridae
6810	Siphoviridae
6811	Unknown
6812	Unknown
6813	Myoviridae
6817	Myoviridae
6818	Myoviridae
6819	Myoviridae
6820	Myoviridae
6822	Unknown
6823	Siphoviridae
6824	Unknown
6825	Siphoviridae
6826	Siphoviridae
6828	Inoviridae
6829	Myoviridae
6831	Siphoviridae
6832	Siphoviridae
6833	Myoviridae
6835	Siphoviridae
6836	Myoviridae

dLGT_phageID	ICTV Phage taxa
6837	Unknown
6838	Podoviridae
6839	Myoviridae
6840	Siphoviridae
6841	Myoviridae
6842	Siphoviridae
6843	Siphoviridae
6844	Siphoviridae
6845	Siphoviridae
6846	Unknown
6847	Siphoviridae
6848	Siphoviridae
6850	Siphoviridae
6851	Siphoviridae
6852	Siphoviridae
6853	Myoviridae
6854	Myoviridae
6855	Podoviridae
6856	Siphoviridae
6857	Siphoviridae
6858	Myoviridae
6859	Myoviridae
6860	Siphoviridae
6861	Siphoviridae
6862	Siphoviridae
6863	Podoviridae
6864	Podoviridae
6865	Siphoviridae
6866	Siphoviridae
6867	Siphoviridae
6868	Siphoviridae
6869	Siphoviridae
6871	Siphoviridae
6872	Siphoviridae
6873	Myoviridae
6874	Siphoviridae
6875	Siphoviridae
6876	Myoviridae
6877	Siphoviridae
6878	Siphoviridae
6879	Unknown
6880	Unknown
6882	Unknown
6883	Siphoviridae
6884	Siphoviridae

dLGT_phageID	ICTV Phage taxa
6885	Siphoviridae
6886	Siphoviridae
6887	Unknown
6888	Unknown
6889	Unknown
6890	Unknown
6891	Siphoviridae
6892	Myoviridae
6893	Myoviridae
6895	Siphoviridae
6896	Podoviridae
6897	Podoviridae
6898	Myoviridae
6900	Myoviridae
6901	Podoviridae
6902	Myoviridae
6903	Podoviridae
6905	Siphoviridae
6907	Podoviridae
6908	Myoviridae
6909	Myoviridae
6910	Inoviridae
6911	Myoviridae
6912	Myoviridae
6913	Myoviridae
6914	Siphoviridae
6915	Siphoviridae
6916	Siphoviridae
6917	Siphoviridae
6918	Siphoviridae
6919	Siphoviridae
6921	Myoviridae
6923	Myoviridae
6924	Podoviridae
6927	Siphoviridae
6930	Siphoviridae
6931	Siphoviridae
6932	Myoviridae
6934	Siphoviridae
6935	Myoviridae
6936	Siphoviridae
6937	Siphoviridae
6938	Siphoviridae
6939	Siphoviridae
6940	Myoviridae

dLGT_phageID	ICTV Phage taxa
6941	Siphoviridae
6942	Siphoviridae
6943	Siphoviridae
6945	Siphoviridae
6946	Siphoviridae
6949	Siphoviridae
6950	Siphoviridae
6951	Siphoviridae
6952	Myoviridae
6953	Myoviridae
6954	Unknown
6955	Siphoviridae
6956	Siphoviridae
6957	Siphoviridae
6958	Podoviridae
6961	Myoviridae
6962	Myoviridae
6963	Unknown
6963	Siphoviridae
6964	Siphoviridae
6965	Myoviridae
6967	Siphoviridae
6968	Unknown
6969	Siphoviridae
6970	Siphoviridae
6971	Unknown
6972	Siphoviridae
6974	Inoviridae
6975	Siphoviridae
6977	Myoviridae
6978	Siphoviridae
6979	Myoviridae
6980	Podoviridae
6982	Podoviridae
6983	Myoviridae
6984	Myoviridae
6985	Podoviridae
6986	Podoviridae
6988	Podoviridae
6989	Podoviridae
6990	Podoviridae
6991	Podoviridae
6992	Myoviridae
6993	Myoviridae
6994	Myoviridae

dLGT_phageID	ICTV Phage taxa
6995	Myoviridae
6996	Siphoviridae
6997	Siphoviridae
6998	Myoviridae
6999	Siphoviridae
7000	Unknown
7001	Siphoviridae
7002	Myoviridae
7003	Siphoviridae
7006	Siphoviridae
7007	Unknown
7008	Inoviridae
7009	Siphoviridae
7011	Siphoviridae
7012	Myoviridae
7013	Siphoviridae
7015	Unknown
7016	Siphoviridae
7017	Siphoviridae
7020	Siphoviridae
7021	Siphoviridae
7023	Unknown
7024	Siphoviridae
7025	Podoviridae
7026	Siphoviridae
7027	Unknown
7028	Unknown
7029	Myoviridae
7031	Siphoviridae
7032	Siphoviridae
7033	Siphoviridae
7034	Myoviridae
7035	Siphoviridae
7036	Myoviridae
7037	Myoviridae
7038	Siphoviridae
7039	Unknown
7040	Myoviridae
7041	Podoviridae
7042	Myoviridae
7043	Myoviridae
7044	Podoviridae
7045	Podoviridae
7046	Podoviridae
7048	Myoviridae

dLGT_phageID	ICTV Phage taxa
7049	Myoviridae
7050	Siphoviridae
7052	Myoviridae
7054	Siphoviridae
7055	Siphoviridae
7056	Myoviridae
7057	Myoviridae
7058	Myoviridae
7059	Myoviridae
7060	Myoviridae
7061	Myoviridae
7062	Podoviridae
7063	Myoviridae
7064	Siphoviridae
7065	Siphoviridae
7066	Myoviridae
7068	Myoviridae
7069	Siphoviridae
7070	Myoviridae
7071	Siphoviridae
7072	Myoviridae
7073	Siphoviridae
7074	Unknown
7075	Siphoviridae
7076	Siphoviridae
7077	Myoviridae
7078	Siphoviridae
7079	Podoviridae
7081	Siphoviridae
7082	Siphoviridae
7084	Myoviridae
7085	Siphoviridae
7088	Siphoviridae
7089	Siphoviridae
7090	Siphoviridae
7091	Unknown
7091	Siphoviridae
7092	Podoviridae
7094	Podoviridae
7094	Siphoviridae
7097	Siphoviridae
7099	Myoviridae
7103	Siphoviridae
7105	Myoviridae
7108	Siphoviridae

dLGT_phageID	ICTV Phage taxa
7109	Myoviridae
7109	Siphoviridae
7110	Myoviridae
7110	Podoviridae
7111	Unknown
7113	Siphoviridae
7115	Siphoviridae
7116	Siphoviridae
7117	Unknown
7118	Podoviridae
7120	Unknown
7121	Unknown
7122	Siphoviridae
7123	Siphoviridae
7124	Siphoviridae
7125	Unknown
7126	Siphoviridae
7127	Podoviridae
7129	Siphoviridae
7130	Siphoviridae
7131	Myoviridae
7132	Myoviridae
7133	Myoviridae
7134	Myoviridae
7135	Siphoviridae
7136	Siphoviridae
7137	Myoviridae
7139	Siphoviridae
7140	Siphoviridae
7141	Myoviridae
7142	Myoviridae
7143	Siphoviridae
7144	Siphoviridae
7145	Podoviridae
7146	Myoviridae
7147	Myoviridae
7148	Myoviridae
7149	Siphoviridae
7150	Podoviridae
7151	Siphoviridae
7152	Myoviridae
7153	Siphoviridae
7154	Siphoviridae
7155	Myoviridae
7156	Unknown

dLGT_phageID	ICTV Phage taxa
7158	Siphoviridae
7159	Myoviridae
7160	Inoviridae
7161	Siphoviridae
7163	Unknown
7164	Unknown
7165	Myoviridae
7166	Siphoviridae
7167	Podoviridae
7168	Podoviridae
7170	Unknown
7171	Siphoviridae
7172	Siphoviridae
7173	Siphoviridae
7174	Siphoviridae
7175	Siphoviridae
7178	Siphoviridae
7179	Siphoviridae
7180	Siphoviridae
7182	Siphoviridae
7184	Myoviridae
7184	Podoviridae
7185	Unknown
7186	Myoviridae
7187	Unknown
7188	Podoviridae
7189	Siphoviridae
7190	Siphoviridae
7191	Siphoviridae
7192	Unknown
7195	Siphoviridae
7198	Podoviridae
7199	Siphoviridae
7206	Unknown
7208	Myoviridae
7208	Siphoviridae
7209	Siphoviridae
7211	Myoviridae
7212	Siphoviridae
7214	Siphoviridae
7215	Siphoviridae
7216	Siphoviridae
7217	Unknown
7219	Siphoviridae
7223	Myoviridae

dLGT_phageID	ICTV Phage taxa
7224	Siphoviridae
7225	Siphoviridae
7226	Myoviridae
7226	Siphoviridae
7229	Siphoviridae
7230	Myoviridae
7230	Siphoviridae
7231	Unknown
7234	Unknown
7234	Siphoviridae
7235	Myoviridae
7236	Siphoviridae
7237	Unknown
7238	Siphoviridae
7241	Siphoviridae
7242	Siphoviridae
7243	Unknown
7243	Siphoviridae
7246	Unknown
7246	Siphoviridae
7247	Siphoviridae
7248	Siphoviridae
7249	Siphoviridae
7251	Siphoviridae
7252	Siphoviridae
7253	Siphoviridae
7254	Siphoviridae
7255	Siphoviridae
7256	Unknown
7256	Siphoviridae
7257	Siphoviridae
7258	Siphoviridae
7259	Podoviridae
7259	Siphoviridae
7260	Myoviridae
7261	Siphoviridae
7262	Myoviridae
7263	Myoviridae
7264	Unknown
7265	Siphoviridae
7266	Siphoviridae
7267	Siphoviridae
7268	Unknown
7270	Siphoviridae
7271	Siphoviridae

dLGT_phageID	ICTV Phage taxa
7272	Podoviridae
7273	Myoviridae
7274	Myoviridae
7275	Unknown
7276	Myoviridae
7277	Unknown
7277	Podoviridae
7279	Myoviridae
7281	Myoviridae
7282	Siphoviridae
7283	Siphoviridae
7284	Siphoviridae
7285	Podoviridae
7286	Siphoviridae
7290	Inoviridae
7291	Myoviridae
7292	Siphoviridae
7293	Siphoviridae
7295	Myoviridae
7299	Myoviridae
7300	Myoviridae
7301	Myoviridae
7301	Podoviridae
7302	Myoviridae
7303	Myoviridae
7303	Unknown
7303	Podoviridae
7304	Siphoviridae
7305	Myoviridae
7305	Siphoviridae
7306	Siphoviridae
7309	Siphoviridae
7311	Siphoviridae
7312	Myoviridae
7312	Unknown
7316	Siphoviridae
7317	Siphoviridae
7319	Unknown
7321	Myoviridae
7322	Myoviridae
7323	Myoviridae
7324	Myoviridae
7325	Myoviridae
7326	Siphoviridae
7327	Myoviridae

dLGT_phageID	ICTV Phage taxa
7328	Myoviridae
7329	Podoviridae
7330	Myoviridae
7331	Unknown
7332	Podoviridae
7333	Myoviridae
7334	Myoviridae
7335	Siphoviridae
7336	Myoviridae
7337	Myoviridae
7337	Unknown
7343	Myoviridae
7345	Siphoviridae
7347	Podoviridae
7349	Podoviridae
7350	Siphoviridae
7351	Myoviridae
7352	Siphoviridae
7353	Myoviridae
7354	Myoviridae
7355	Siphoviridae
7356	Siphoviridae
7357	Inoviridae
7358	Siphoviridae
7359	Siphoviridae
7363	Myoviridae
7364	Myoviridae
7365	Podoviridae
7366	Myoviridae
7367	Siphoviridae
7368	Siphoviridae
7369	Siphoviridae
7370	Siphoviridae
7371	Siphoviridae
7372	Podoviridae
7372	Siphoviridae
7376	Unknown
7377	Siphoviridae
7379	Siphoviridae
7380	Siphoviridae
7381	Siphoviridae
7383	Myoviridae
7384	Unknown
7385	Unknown
7392	Myoviridae

dLGT_phageID	ICTV Phage taxa
7392	Siphoviridae
7393	Unknown
7393	Siphoviridae
7394	Siphoviridae
7395	Siphoviridae
7396	Siphoviridae
7397	Podoviridae
8001	Siphoviridae
8002	Unknown
8003	Unknown
8004	Siphoviridae
8005	Siphoviridae
8006	Siphoviridae
8007	Myoviridae
8008	Siphoviridae
8009	Myoviridae
8010	Siphoviridae
8011	Myoviridae
8012	Podoviridae
8013	Siphoviridae
8014	Siphoviridae
8015	Myoviridae
8016	Siphoviridae
8017	Siphoviridae
8018	Siphoviridae
8019	Unknown
8020	Unknown
8021	Myoviridae
8022	Siphoviridae
8023	Siphoviridae
8024	Myoviridae
8025	Unknown
8026	Unknown
8027	Siphoviridae
8028	Myoviridae
8029	Unknown
8030	Myoviridae
8031	Siphoviridae
8032	Podoviridae
8033	Myoviridae
8034	Unknown
8035	Siphoviridae
8036	Siphoviridae
8037	Siphoviridae
8038	Myoviridae

dLGT_phageID	ICTV Phage taxa
8039	Siphoviridae
8040	Myoviridae
8041	Myoviridae
8042	Podoviridae
8043	Myoviridae
8044	Podoviridae
8045	Myoviridae
8046	Unknown
8047	Podoviridae
8048	Siphoviridae
8049	Myoviridae
8050	Siphoviridae
8051	Siphoviridae
8052	Myoviridae
8053	Podoviridae
8054	Podoviridae
8055	Podoviridae
8056	Myoviridae
8057	Podoviridae
8058	Siphoviridae
8059	Siphoviridae
8060	Unknown
8061	Siphoviridae
8062	Myoviridae
8063	Myoviridae
8064	Siphoviridae
8065	Siphoviridae
8066	Siphoviridae
8067	Unknown
8068	Myoviridae
8069	Unknown
8070	Siphoviridae
8071	Myoviridae
8072	Myoviridae
8073	Podoviridae
8074	Siphoviridae
8075	Siphoviridae
8076	Siphoviridae
8077	Siphoviridae
8078	Siphoviridae
8079	Siphoviridae
8080	Siphoviridae
8081	Myoviridae
8082	Myoviridae
8083	Siphoviridae

dLGT_phageID	ICTV Phage taxa
8084	Myoviridae
8085	Siphoviridae
8086	Myoviridae
8087	Myoviridae
8088	Podoviridae
8089	Siphoviridae
8090	Siphoviridae
8091	Siphoviridae
8092	Myoviridae
8093	Unknown
8094	Siphoviridae
8095	Unknown
8096	Siphoviridae
8097	Unknown
8098	Siphoviridae
8099	Siphoviridae
8100	Myoviridae
8101	Siphoviridae
8102	Unknown
8103	Siphoviridae
8104	Siphoviridae
8105	Siphoviridae
8106	Myoviridae
8107	Siphoviridae
8108	Siphoviridae
8109	Unknown
8110	Siphoviridae
8111	Siphoviridae
8112	Unknown
8113	Myoviridae
8114	Unknown
8115	Siphoviridae
8116	Siphoviridae
8117	Corticoviridae
8118	Podoviridae
8119	Myoviridae
8120	Siphoviridae
8121	Myoviridae
8122	Siphoviridae
8123	Myoviridae
8124	Myoviridae
8125	Siphoviridae
8126	Unknown
8127	Unknown
8128	Siphoviridae

dLGT_phageID	ICTV Phage taxa
8129	Siphoviridae
8130	Siphoviridae
8131	Siphoviridae
8132	Myoviridae
8133	Myoviridae
8134	Myoviridae
8135	Myoviridae
8136	Siphoviridae
8137	Siphoviridae
8138	Myoviridae
8139	Unknown
8140	Unknown
8141	Unknown
8142	Myoviridae
8143	Inoviridae
8144	Siphoviridae
8145	Siphoviridae
8146	Podoviridae
8147	Siphoviridae
8148	Siphoviridae
8149	Podoviridae
8150	Myoviridae
8151	Siphoviridae
8152	Siphoviridae
8153	Siphoviridae
8154	Siphoviridae
8155	Myoviridae
8156	Myoviridae
8157	Myoviridae
8158	Myoviridae
8159	Podoviridae
8160	Podoviridae
8161	Podoviridae
8162	Myoviridae
8163	Myoviridae
8164	Siphoviridae
8165	Podoviridae
8166	Myoviridae
8167	Siphoviridae
8168	Myoviridae
8169	Siphoviridae
8170	Siphoviridae
8171	Siphoviridae
8172	Myoviridae
8173	Myoviridae

dLGT_phageID	ICTV Phage taxa
8174	Siphoviridae
8175	Siphoviridae
8176	Siphoviridae
8177	Tectiviridae
8178	Siphoviridae
8179	Myoviridae
8180	Siphoviridae
8181	Siphoviridae
8182	Siphoviridae
8183	Siphoviridae
8184	Siphoviridae
8185	Myoviridae
8186	Siphoviridae
8187	Myoviridae
8188	Siphoviridae
8189	Siphoviridae
8190	Siphoviridae
8191	Unknown
8192	Unknown
8193	Siphoviridae
8194	Siphoviridae
8195	Myoviridae
8196	Siphoviridae
8197	Inoviridae
8198	Myoviridae
8199	Myoviridae
8200	Siphoviridae
8201	Unknown
8202	Myoviridae
8203	Siphoviridae
8204	Unknown
8205	Siphoviridae
8206	Siphoviridae
8207	Myoviridae
8208	Myoviridae
8209	Siphoviridae
8210	Siphoviridae
8211	Myoviridae
8212	Siphoviridae
8213	Siphoviridae
8214	Siphoviridae
8215	Podoviridae
8216	Myoviridae
8217	Siphoviridae
8218	Myoviridae

dLGT_phageID	ICTV Phage taxa
8219	Myoviridae
8220	Siphoviridae
8221	Unknown
8222	Myoviridae
8223	Siphoviridae
8224	Siphoviridae
8225	Siphoviridae
8226	Podoviridae
8227	Myoviridae
8228	Myoviridae
8229	Unknown
8230	Podoviridae
8231	Podoviridae
8232	Myoviridae
8233	Podoviridae
8234	Siphoviridae
8235	Myoviridae
8236	Myoviridae
8237	Siphoviridae
8238	Siphoviridae
8239	Siphoviridae
8240	Podoviridae
8241	Siphoviridae
8242	Myoviridae
8243	Podoviridae
8244	Siphoviridae
8245	Myoviridae
8246	Siphoviridae
8247	Podoviridae
8248	Myoviridae
8249	Siphoviridae
8250	Podoviridae
8251	Unknown
8252	Unknown
8253	Siphoviridae
8254	Myoviridae
8255	Myoviridae
8256	Myoviridae
8257	Podoviridae
8258	Unknown
8259	Unknown
8260	Myoviridae
8261	Siphoviridae
8262	Siphoviridae
8263	Myoviridae

dLGT_phageID	ICTV Phage taxa
8264	Myoviridae
8265	Myoviridae
8266	Myoviridae
8267	Myoviridae
8268	Unknown
8269	Unknown
8270	Siphoviridae
8271	Siphoviridae
8272	Myoviridae
8273	Podoviridae
8274	Siphoviridae
8275	Siphoviridae
8276	Siphoviridae
8277	Inoviridae
8278	Myoviridae
8279	Myoviridae
8280	Myoviridae
8281	Siphoviridae
8282	Myoviridae
8283	Siphoviridae
8284	Myoviridae
8285	Siphoviridae
8286	Myoviridae
8287	Myoviridae
8288	Siphoviridae
8289	Myoviridae
8290	Myoviridae
8291	Myoviridae
8292	Myoviridae
8293	Siphoviridae
8294	Myoviridae
8295	Myoviridae
8296	Myoviridae
8297	Siphoviridae
8298	Siphoviridae
8299	Myoviridae
8300	Siphoviridae
8301	Siphoviridae
8302	Siphoviridae
8303	Siphoviridae
8304	Siphoviridae
8305	Myoviridae
8306	Siphoviridae
8307	Podoviridae
8308	Myoviridae

dLGT_phageID	ICTV Phage taxa
8309	Myoviridae
8310	Unknown
8311	Siphoviridae
8312	Siphoviridae
8313	Unknown
8314	Unknown
8315	Siphoviridae
8316	Myoviridae
8317	Myoviridae
8318	Podoviridae
8319	Podoviridae
8320	Siphoviridae
8321	Myoviridae
8322	Myoviridae
8323	Siphoviridae
8324	Myoviridae
8325	Myoviridae
8326	Myoviridae
8327	Myoviridae
8328	Siphoviridae
8329	Myoviridae
8330	Myoviridae
8331	Siphoviridae
8332	Podoviridae
8333	Siphoviridae
8334	Siphoviridae
8335	Myoviridae
8336	Siphoviridae
8337	Podoviridae
8338	Podoviridae
8339	Siphoviridae
8340	Siphoviridae
8341	Siphoviridae
8342	Siphoviridae
8343	Siphoviridae
8344	Myoviridae
8345	Myoviridae
8346	Podoviridae
8347	Myoviridae
8348	Siphoviridae
8349	Siphoviridae
8350	Siphoviridae
8351	Siphoviridae
8352	Siphoviridae
8353	Plasmaviridae

dLGT_phageID	ICTV Phage taxa
8354	Siphoviridae
8355	Siphoviridae
8356	Myoviridae
8357	Siphoviridae
8358	Siphoviridae
8359	Siphoviridae
8360	Siphoviridae
8361	Myoviridae
8362	Siphoviridae
8363	Siphoviridae
8364	Siphoviridae
8365	Siphoviridae
8366	Unknown
8367	Myoviridae
8368	Siphoviridae
8369	Myoviridae
8370	Unknown
8371	Inoviridae
8372	Podoviridae
8373	Myoviridae
8374	Unknown
8375	Siphoviridae
8376	Siphoviridae
8377	Siphoviridae
8378	Siphoviridae
8379	Podoviridae
8380	Unknown
8381	Myoviridae
8382	Tectiviridae
8383	Siphoviridae
8384	Myoviridae
8385	Siphoviridae
8386	Myoviridae
8387	Myoviridae
8388	Siphoviridae
8389	Siphoviridae
8390	Siphoviridae
8391	Myoviridae
8392	Siphoviridae
8393	Siphoviridae
8394	Inoviridae
8395	Unknown
8396	Myoviridae
8397	Myoviridae
8398	Myoviridae

dLGT_phageID	ICTV Phage taxa
8399	Myoviridae
8400	Siphoviridae
8401	Siphoviridae
8402	Podoviridae
8403	Siphoviridae
8404	Podoviridae
8405	Myoviridae
8406	Siphoviridae
8407	Podoviridae
8408	Siphoviridae
8409	Siphoviridae
8410	Siphoviridae
8411	Siphoviridae
8412	Siphoviridae
8413	Inoviridae
8414	Myoviridae
8415	Unknown
8416	Myoviridae
8417	Siphoviridae
8418	Siphoviridae
8419	Siphoviridae
8420	Siphoviridae
8421	Siphoviridae
8422	Unknown
8423	Siphoviridae
8424	Siphoviridae
8425	Unknown
8426	Siphoviridae
8427	Unknown
8428	Siphoviridae
8429	Siphoviridae
8430	Siphoviridae
8431	Siphoviridae
8432	Myoviridae
8433	Myoviridae
8434	Siphoviridae
8435	Siphoviridae
8436	Siphoviridae
8437	Siphoviridae
8438	Myoviridae
8439	Siphoviridae
8440	Siphoviridae
8441	Myoviridae
8442	Unknown
8443	Siphoviridae

dLGT_phageID	ICTV Phage taxa
8444	Podoviridae
8445	Unknown
8446	Siphoviridae
8447	Unknown
8448	Siphoviridae
8449	Siphoviridae
8450	Myoviridae
8451	Siphoviridae
8452	Siphoviridae
8453	Siphoviridae
8454	Podoviridae
8455	Siphoviridae
8456	Unknown
8457	Unknown
8458	Siphoviridae
8459	Siphoviridae
8460	Myoviridae
8461	Podoviridae
8462	Myoviridae
8463	Myoviridae
8464	Siphoviridae
8465	Myoviridae
8466	Myoviridae
8467	Myoviridae
8468	Myoviridae
8469	Siphoviridae
8470	Myoviridae
8471	Siphoviridae
8472	Myoviridae
8473	Myoviridae
8474	Myoviridae
8475	Myoviridae
8476	Myoviridae
8477	Myoviridae
8478	Myoviridae
8479	Unknown
8480	Myoviridae
8481	Myoviridae
8482	Siphoviridae
8483	Myoviridae
8484	Unknown
8485	Siphoviridae
8486	Myoviridae
8487	Myoviridae
8488	Siphoviridae

dLGT_phageID	ICTV Phage taxa
8489	Siphoviridae
8490	Siphoviridae
8491	Myoviridae
8492	Myoviridae
8493	Siphoviridae
8494	Myoviridae
8495	Unknown
8496	Siphoviridae
8497	Siphoviridae
8498	Myoviridae
8499	Myoviridae
8500	Inoviridae
8501	Siphoviridae
8502	Siphoviridae
8503	Myoviridae
8504	Myoviridae
8505	Podoviridae
8506	Myoviridae
8507	Unknown
8508	Siphoviridae
8509	Siphoviridae
8510	Myoviridae
8511	Myoviridae
8512	Siphoviridae
8513	Unknown
8514	Podoviridae
8515	Myoviridae
8516	Siphoviridae
8517	Myoviridae
8518	Siphoviridae
8519	Siphoviridae
8520	Siphoviridae
8521	Myoviridae
8522	Siphoviridae
8523	Siphoviridae
8524	Siphoviridae
8525	Siphoviridae
8526	Siphoviridae
8527	Myoviridae
8528	Myoviridae
8529	Siphoviridae
8530	Siphoviridae
8531	Siphoviridae
8532	Siphoviridae
8533	Siphoviridae

dLGT_phageID	ICTV Phage taxa
8534	Siphoviridae
8535	Myoviridae
8536	Siphoviridae
8537	Unknown
8538	Myoviridae
8539	Siphoviridae
8540	Unknown
8541	Podoviridae
8542	Podoviridae
8543	Siphoviridae
8544	Unknown
8545	Siphoviridae
8546	Unknown
8547	Podoviridae
8548	Siphoviridae
8549	Siphoviridae
8550	Unknown
8551	Myoviridae
8552	Inoviridae
8553	Myoviridae
8554	Siphoviridae
8555	Siphoviridae
8556	Siphoviridae
8557	Myoviridae
8558	Myoviridae
8559	Siphoviridae
8560	Siphoviridae
8561	Siphoviridae
8562	Siphoviridae
8563	Myoviridae
8564	Siphoviridae
8565	Siphoviridae
8566	Myoviridae
8567	Myoviridae
8568	Myoviridae
8569	Myoviridae
8570	Myoviridae
8571	Myoviridae
8572	Siphoviridae
8573	Myoviridae
8574	Myoviridae
8575	Myoviridae
8576	Myoviridae
8577	Myoviridae
8578	Siphoviridae

dLGT_phageID	ICTV Phage taxa
8579	Podoviridae
8580	Unknown
8581	Siphoviridae
8582	Siphoviridae
8583	Myoviridae
8584	Siphoviridae
8585	Siphoviridae
8586	Myoviridae
8587	Siphoviridae
8588	Siphoviridae
8589	Myoviridae
8590	Siphoviridae
8591	Myoviridae
8592	Myoviridae
8593	Myoviridae
8594	Siphoviridae
8595	Siphoviridae
8596	Myoviridae
8597	Siphoviridae
8598	Siphoviridae
8599	Unknown
8600	Podoviridae
8601	Myoviridae
8602	Myoviridae
8603	Podoviridae
8604	Siphoviridae
8605	Podoviridae
8606	Podoviridae
8607	Siphoviridae
8608	Siphoviridae
8609	Siphoviridae
8610	Unknown
8611	Siphoviridae
8612	Siphoviridae
8613	Siphoviridae
8614	Unknown
8615	Siphoviridae
8616	Siphoviridae
8617	Siphoviridae
8618	Myoviridae
8619	Myoviridae
8620	Unknown
8621	Myoviridae
8622	Myoviridae
8623	Myoviridae

dLGT_phageID	ICTV Phage taxa
8624	Myoviridae
8625	Myoviridae
8626	Siphoviridae
8627	Siphoviridae
8628	Podoviridae
8629	Siphoviridae
8630	Siphoviridae
8631	Unknown
8632	Siphoviridae
8633	Siphoviridae
8634	Podoviridae
8635	Siphoviridae
8636	Siphoviridae
8637	Siphoviridae
8638	Siphoviridae
8639	Myoviridae
8640	Siphoviridae
8641	Inoviridae
8642	Siphoviridae
8643	Myoviridae
8644	Unknown
8645	Siphoviridae
8646	Siphoviridae
8647	Podoviridae
8648	Inoviridae
8649	Siphoviridae
8650	Siphoviridae
8651	Siphoviridae
8652	Siphoviridae
8653	Unknown
8654	Myoviridae
8655	Myoviridae
8656	Siphoviridae
8657	Siphoviridae
8658	Siphoviridae
8659	Siphoviridae
8660	Siphoviridae
8661	Siphoviridae
8662	Unknown
8663	Unknown
8664	Myoviridae
8665	Siphoviridae
8666	Siphoviridae
8667	Siphoviridae
8668	Myoviridae

dLGT_phageID	ICTV Phage taxa
8669	Myoviridae
8670	Myoviridae
8671	Siphoviridae
8672	Myoviridae
8673	Siphoviridae
8674	Siphoviridae
8675	Siphoviridae
8676	Siphoviridae
8677	Inoviridae
8678	Myoviridae
8679	Myoviridae
8680	Siphoviridae
8681	Siphoviridae
8682	Siphoviridae
8683	Myoviridae
8684	Siphoviridae
8685	Siphoviridae
8686	Myoviridae
8687	Myoviridae
8688	Unknown
8689	Unknown
8690	Podoviridae
8691	Unknown
8692	Siphoviridae
8693	Siphoviridae
8694	Podoviridae
8695	Myoviridae
8696	Siphoviridae
8697	Unknown
8698	Unknown
8699	Unknown
8700	Siphoviridae
8701	Siphoviridae
8702	Siphoviridae
8703	Myoviridae
8704	Unknown
8705	Siphoviridae
8706	Myoviridae
8707	Siphoviridae
8708	Myoviridae
8709	Siphoviridae
8710	Myoviridae
8711	Myoviridae
8712	Myoviridae
8713	Myoviridae

dLGT_phageID	ICTV Phage taxa
8714	Siphoviridae
8715	Unknown
8716	Siphoviridae
8717	Myoviridae
8718	Myoviridae
8719	Myoviridae
8720	Siphoviridae
8721	Myoviridae
8722	Siphoviridae
8723	Podoviridae
8724	Siphoviridae
8725	Siphoviridae
8726	Unknown
8727	Unknown
8728	Siphoviridae
8729	Unknown
8730	Podoviridae
8731	Siphoviridae
8732	Siphoviridae
8733	Podoviridae
8734	Unknown
8735	Siphoviridae
8736	Unknown
8737	Siphoviridae
8738	Siphoviridae
8739	Siphoviridae
8740	Siphoviridae
8741	Myoviridae
8742	Myoviridae
8743	Myoviridae
8744	Myoviridae
8745	Myoviridae
8746	Siphoviridae
8747	Myoviridae
8748	Siphoviridae
8749	Siphoviridae
8750	Podoviridae
8751	Myoviridae
8752	Siphoviridae
8753	Siphoviridae
8754	Myoviridae
8755	Siphoviridae
8756	Siphoviridae
8757	Siphoviridae
8758	Siphoviridae

dLGT_phageID	ICTV Phage taxa
8759	Podoviridae
8760	Siphoviridae
8761	Siphoviridae
8762	Siphoviridae
8763	Unknown
8764	Myoviridae
8765	Siphoviridae
8766	Siphoviridae
8767	Siphoviridae
8768	Unknown
8769	Siphoviridae
8770	Myoviridae
8771	Myoviridae
8772	Myoviridae
8773	Unknown
8774	Siphoviridae
8775	Siphoviridae
8776	Myoviridae
8777	Myoviridae
8778	Siphoviridae
8779	Myoviridae
8780	Myoviridae
8781	Podoviridae
8782	Podoviridae
8783	Unknown
8784	Siphoviridae
8785	Siphoviridae
8786	Siphoviridae
8787	Siphoviridae
8788	Myoviridae
8789	Unknown
8790	Myoviridae
8791	Myoviridae
8792	Siphoviridae
8793	Unknown
8794	Podoviridae
8795	Siphoviridae
8796	Myoviridae
8797	Siphoviridae
8798	Unknown
8799	Siphoviridae
8800	Siphoviridae
8801	Siphoviridae
8802	Podoviridae
8803	Podoviridae

dLGT_phageID	ICTV Phage taxa
8804	Podoviridae
8805	Siphoviridae
8806	Myoviridae
8807	Myoviridae
8808	Myoviridae
8809	Siphoviridae
8810	Myoviridae
8811	Siphoviridae
8812	Siphoviridae
8813	Podoviridae
8814	Podoviridae
8815	Myoviridae
8816	Siphoviridae
8817	Myoviridae
8818	Podoviridae
8819	Podoviridae
8820	Myoviridae
8821	Siphoviridae
8822	Myoviridae
8823	Podoviridae
8824	Unknown
8825	Inoviridae
8826	Siphoviridae
8827	Myoviridae
8828	Myoviridae
8829	Siphoviridae
8830	Myoviridae
8831	Myoviridae
8832	Siphoviridae
8833	Myoviridae
8834	Myoviridae
8835	Myoviridae
8836	Myoviridae
8837	Myoviridae
8838	Myoviridae
8839	Siphoviridae
8840	Unknown
8841	Tectiviridae
8842	Siphoviridae
8843	Myoviridae
8844	Myoviridae
8845	Siphoviridae
8846	Myoviridae
8847	Myoviridae
8848	Myoviridae

dLGT_phageID	ICTV Phage taxa
8849	Myoviridae
8850	Siphoviridae
8851	Myoviridae
8852	Siphoviridae
8853	Unknown
8854	Siphoviridae
8855	Myoviridae
8856	Myoviridae
8857	Myoviridae
8858	Siphoviridae
8859	Myoviridae
8860	Myoviridae
8861	Myoviridae
8862	Siphoviridae
8863	Myoviridae
8864	Myoviridae
8865	Myoviridae
8866	Siphoviridae
8867	Myoviridae
8868	Myoviridae
8869	Podoviridae
8870	Myoviridae
8871	Myoviridae
8872	Myoviridae
8873	Myoviridae
8874	Siphoviridae
8875	Myoviridae
8876	Unknown
8877	Myoviridae
8878	Siphoviridae
8879	Myoviridae
8880	Myoviridae
8881	Myoviridae
8882	Myoviridae
8883	Siphoviridae
8884	Siphoviridae
8885	Unknown
8886	Myoviridae
8887	Unknown
8888	Siphoviridae
8889	Myoviridae
8890	Myoviridae
8891	Podoviridae
8892	Siphoviridae
8893	Siphoviridae

dLGT_phageID	ICTV Phage taxa
8894	Myoviridae
8895	Myoviridae
8896	Myoviridae
8897	Siphoviridae
8898	Myoviridae
8899	Podoviridae
8900	Siphoviridae
8901	Unknown
8902	Myoviridae
8903	Siphoviridae
8904	Siphoviridae
8905	Siphoviridae
8906	Siphoviridae
8907	Unknown
8908	Siphoviridae
8909	Myoviridae
8910	Unknown
8911	Unknown
8912	Myoviridae
8913	Myoviridae
8914	Siphoviridae
8915	Myoviridae
8916	Podoviridae
8917	Siphoviridae
8918	Podoviridae
8919	Siphoviridae
8920	Podoviridae
8921	Myoviridae
8922	Siphoviridae
8923	Myoviridae
8924	Myoviridae
8925	Myoviridae
8926	Myoviridae
8927	Myoviridae
8928	Siphoviridae
8929	Siphoviridae
8930	Siphoviridae
8931	Myoviridae
8932	Myoviridae
8933	Siphoviridae
8934	Myoviridae
8935	Myoviridae
8936	Myoviridae
8937	Siphoviridae
8938	Siphoviridae

dLGT_phageID	ICTV Phage taxa
8939	Myoviridae
8940	Siphoviridae
8941	Siphoviridae
8942	Inoviridae
8943	Plasmaviridae
8944	Siphoviridae
8945	Siphoviridae
8946	Siphoviridae
8947	Myoviridae
8948	Siphoviridae
8949	Siphoviridae
8950	Unknown
8951	Siphoviridae
8952	Siphoviridae
8953	Siphoviridae
8954	Podoviridae
8955	Siphoviridae
8956	Siphoviridae
8957	Unknown
8958	Siphoviridae
8959	Siphoviridae
8960	Siphoviridae
8961	Myoviridae
8962	Siphoviridae
8963	Myoviridae
8964	Inoviridae
8965	Myoviridae
8966	Myoviridae
8967	Siphoviridae
8968	Siphoviridae
8969	Myoviridae
8970	Siphoviridae
8971	Unknown
8972	Podoviridae
8973	Siphoviridae
8974	Siphoviridae
8975	Podoviridae
8976	Myoviridae
8977	Siphoviridae
8978	Myoviridae
8979	Myoviridae
8980	Siphoviridae
8981	Siphoviridae
8982	Myoviridae
8983	Siphoviridae

dLGT_phageID	ICTV Phage taxa
8984	Podoviridae
8985	Myoviridae
8986	Siphoviridae
8987	Podoviridae
8988	Myoviridae
8989	Siphoviridae
8990	Myoviridae
8991	Unknown
8992	Myoviridae
8993	Unknown
8994	Myoviridae
8995	Myoviridae
8996	Siphoviridae
8997	Siphoviridae
8998	Siphoviridae
8999	Myoviridae
9000	Siphoviridae
9001	Siphoviridae
9002	Podoviridae
9003	Myoviridae
9004	Siphoviridae
9005	Tectiviridae
9006	Myoviridae
9007	Unknown
9008	Siphoviridae
9009	Siphoviridae
9010	Myoviridae
9011	Siphoviridae
9012	Myoviridae
9013	Unknown
9014	Siphoviridae
9015	Siphoviridae
9016	Unknown
9017	Siphoviridae
9018	Siphoviridae
9019	Siphoviridae
9020	Myoviridae
9021	Siphoviridae
9022	Siphoviridae
9023	Siphoviridae
9024	Siphoviridae
9025	Siphoviridae
9026	Siphoviridae
9027	Siphoviridae
9028	Siphoviridae

dLGT_phageID	ICTV Phage taxa
9029	Siphoviridae
9030	Unknown
9031	Siphoviridae
9032	Siphoviridae
9033	Siphoviridae
9034	Siphoviridae
9035	Myoviridae
9036	Myoviridae
9037	Siphoviridae
9038	Siphoviridae
9039	Siphoviridae
9040	Unknown
9041	Unknown
9042	Siphoviridae
9043	Siphoviridae
9044	Siphoviridae
9045	Siphoviridae
9046	Podoviridae
9047	Myoviridae
9048	Siphoviridae
9049	Myoviridae
9050	Siphoviridae
9051	Myoviridae
9052	Siphoviridae
9053	Siphoviridae
9054	Myoviridae
9055	Unknown
9056	Siphoviridae
9057	Podoviridae
9058	Myoviridae
9059	Siphoviridae
9060	Myoviridae
9061	Siphoviridae
9062	Myoviridae
9063	Siphoviridae
9064	Myoviridae
9065	Siphoviridae
9066	Unknown
9067	Myoviridae
9068	Siphoviridae
9069	Unknown
9070	Myoviridae
9071	Unknown
9072	Siphoviridae
9073	Siphoviridae

dLGT_phageID	ICTV Phage taxa
9074	Siphoviridae
9075	Siphoviridae
9076	Myoviridae
9077	Myoviridae
9078	Podoviridae
9079	Myoviridae
9080	Unknown
9081	Myoviridae
9082	Siphoviridae
9083	Myoviridae
9084	Myoviridae
9085	Siphoviridae
9086	Siphoviridae
9087	Myoviridae
9088	Myoviridae
9089	Unknown
9090	Myoviridae
9091	Podoviridae
9092	Siphoviridae
9093	Podoviridae
9094	Siphoviridae
9095	Myoviridae
9096	Siphoviridae
9097	Siphoviridae
9098	Siphoviridae
9099	Unknown
9100	Siphoviridae
9101	Siphoviridae
9102	Myoviridae
9103	Siphoviridae
9104	Myoviridae
9105	Myoviridae
9106	Plasmaviridae
9107	Siphoviridae
9108	Myoviridae
9109	Siphoviridae
9110	Siphoviridae
9111	Siphoviridae
9112	Myoviridae
9113	Siphoviridae
9114	Siphoviridae
9115	Siphoviridae
9116	Siphoviridae
9117	Myoviridae
9118	Myoviridae

dLGT_phageID	ICTV Phage taxa
9119	Siphoviridae
9120	Siphoviridae
9121	Unknown
9122	Siphoviridae
9123	Unknown
9124	Myoviridae
9125	Podoviridae
9126	Podoviridae
9127	Siphoviridae
9128	Myoviridae
9129	Siphoviridae
9130	Siphoviridae
9131	Myoviridae
9132	Siphoviridae
9133	Podoviridae
9134	Siphoviridae
9135	Siphoviridae
9136	Unknown
9137	Siphoviridae
9138	Siphoviridae
9139	Podoviridae
9140	Siphoviridae
9141	Myoviridae
9142	Myoviridae
9143	Siphoviridae
9144	Podoviridae
9145	Siphoviridae
9146	Myoviridae
9147	Myoviridae
9148	Myoviridae
9149	Siphoviridae
9150	Inoviridae
9151	Podoviridae
9152	Myoviridae
9153	Siphoviridae
9154	Myoviridae
9155	Myoviridae
9156	Siphoviridae
9157	Siphoviridae
9158	Siphoviridae
9159	Siphoviridae
9160	Podoviridae
9161	Siphoviridae
9162	Myoviridae
9163	Myoviridae

dLGT_phageID	ICTV Phage taxa
9164	Siphoviridae
9165	Myoviridae
9166	Myoviridae
9167	Myoviridae
9168	Myoviridae
9169	Inoviridae
9170	Myoviridae
9171	Unknown
9172	Siphoviridae
9173	Siphoviridae
9174	Myoviridae
9175	Myoviridae
9176	Unknown
9177	Siphoviridae
9178	Siphoviridae
9179	Myoviridae
9180	Myoviridae
9181	Siphoviridae
9182	Myoviridae
9183	Podoviridae
9184	Podoviridae
9185	Siphoviridae
9186	Podoviridae
9187	Myoviridae
9188	Siphoviridae
9189	Siphoviridae
9190	Myoviridae
9191	Siphoviridae
9192	Podoviridae
9193	Myoviridae
9194	Myoviridae
9195	Myoviridae
9196	Myoviridae
9197	Siphoviridae
9198	Siphoviridae
9199	Myoviridae
9200	Siphoviridae
9201	Myoviridae
9202	Myoviridae
9203	Siphoviridae
9204	Siphoviridae
9205	Siphoviridae
9206	Myoviridae
9207	Myoviridae
9208	Siphoviridae

dLGT_phageID	ICTV Phage taxa
9209	Myoviridae
9210	Myoviridae
9211	Unknown
9212	Tectiviridae
9213	Podoviridae
9214	Siphoviridae
9215	Unknown
9216	Myoviridae
9217	Myoviridae
9218	Siphoviridae
9219	Myoviridae
9220	Myoviridae
9221	Myoviridae
9222	Siphoviridae
9223	Podoviridae
9224	Siphoviridae
9225	Siphoviridae
9226	Siphoviridae
9227	Siphoviridae
9228	Podoviridae
9229	Siphoviridae
9230	Siphoviridae
9231	Unknown
9232	Myoviridae
9233	Unknown
9234	Siphoviridae
9235	Siphoviridae
9236	Unknown
9237	Siphoviridae
9238	Siphoviridae
9239	Podoviridae
9240	Siphoviridae
9241	Unknown
9242	Myoviridae
9243	Myoviridae
9244	Myoviridae
9245	Unknown
9246	Myoviridae
9247	Myoviridae
9248	Siphoviridae
9249	Myoviridae
9250	Myoviridae
9251	Siphoviridae
9252	Myoviridae
9253	Unknown

dLGT_phageID	ICTV Phage taxa
9254	Siphoviridae
9255	Siphoviridae
9256	Myoviridae
9257	Siphoviridae
9258	Podoviridae
9259	Siphoviridae
9260	Siphoviridae
9261	Siphoviridae
9262	Myoviridae
9263	Myoviridae
9264	Siphoviridae
9265	Siphoviridae
9266	Siphoviridae
9267	Unknown
9268	Myoviridae
9269	Siphoviridae
9270	Siphoviridae
9271	Unknown
9272	Unknown
9273	Siphoviridae
9274	Unknown
9275	Siphoviridae
9276	Siphoviridae
9277	Siphoviridae
9278	Myoviridae
9279	Myoviridae
9280	Siphoviridae
9281	Siphoviridae
9282	Siphoviridae
9283	Siphoviridae
9284	Myoviridae
9285	Podoviridae
9286	Myoviridae
9287	Siphoviridae
9288	Siphoviridae
9289	Unknown
9290	Myoviridae
9291	Siphoviridae
9292	Myoviridae
9293	Siphoviridae
9294	Siphoviridae
9295	Unknown
9296	Myoviridae
9297	Podoviridae
9298	Unknown

dLGT_phageID	ICTV Phage taxa
9299	Myoviridae
9300	Siphoviridae
9301	Siphoviridae
9302	Siphoviridae
9303	Myoviridae
9304	Unknown
9305	Siphoviridae
9306	Myoviridae
9307	Myoviridae
9308	Podoviridae
9309	Siphoviridae
9310	Myoviridae
9311	Siphoviridae
9312	Siphoviridae
9313	Myoviridae
9314	Myoviridae
9315	Myoviridae
9316	Unknown
9317	Myoviridae
9318	Siphoviridae
9319	Siphoviridae
9320	Siphoviridae
9321	Siphoviridae
9322	Siphoviridae
9323	Siphoviridae
9324	Siphoviridae
9325	Siphoviridae
9326	Siphoviridae
9327	Myoviridae
9328	Siphoviridae
9329	Myoviridae
9330	Siphoviridae
9331	Siphoviridae
9332	Siphoviridae
9333	Siphoviridae
9334	Unknown
9335	Myoviridae
9336	Siphoviridae
9337	Siphoviridae
9338	Siphoviridae
9339	Siphoviridae
9340	Siphoviridae
9341	Siphoviridae
9342	Siphoviridae
9343	Siphoviridae

dLGT_phageID	ICTV Phage taxa
9344	Unknown
9345	Unknown
9346	Siphoviridae
9347	Unknown
9348	Siphoviridae
9349	Myoviridae
9350	Myoviridae
9351	Myoviridae
9352	Myoviridae
9353	Siphoviridae
9354	Myoviridae
9355	Podoviridae
9356	Podoviridae
9357	Siphoviridae
9358	Unknown
9359	Podoviridae
9360	Siphoviridae
9361	Siphoviridae
9362	Myoviridae
9363	Myoviridae
9364	Siphoviridae
9365	Unknown
9366	Myoviridae
9367	Siphoviridae
9368	Siphoviridae
9369	Myoviridae
9370	Siphoviridae
9371	Siphoviridae
9372	Siphoviridae
9373	Siphoviridae
9374	Siphoviridae
9375	Siphoviridae
9376	Siphoviridae
9377	Myoviridae
9378	Siphoviridae
9379	Unknown
9380	Siphoviridae
9381	Podoviridae
9382	Myoviridae
9383	Siphoviridae
9384	Siphoviridae
9385	Siphoviridae
9386	Siphoviridae
9387	Siphoviridae
9388	Siphoviridae

dLGT_phageID	ICTV Phage taxa
9389	Myoviridae
9390	Unknown
9391	Siphoviridae
9392	Myoviridae
9393	Myoviridae
9394	Siphoviridae
9395	Myoviridae
9396	Tectiviridae
9397	Siphoviridae
9398	Unknown
9399	Siphoviridae
9400	Siphoviridae
9401	Siphoviridae
9402	Inoviridae
9403	Unknown
9404	Myoviridae
9405	Myoviridae
9406	Unknown
9407	Siphoviridae
9408	Inoviridae
9409	Siphoviridae
9410	Siphoviridae
9411	Siphoviridae
9412	Siphoviridae
9413	Siphoviridae
9414	Siphoviridae
9415	Siphoviridae
9416	Siphoviridae
9417	Myoviridae
9418	Myoviridae
9419	Siphoviridae
9420	Siphoviridae
9421	Myoviridae
9422	Siphoviridae
9423	Siphoviridae
9424	Siphoviridae
9425	Myoviridae
9426	Plasmaviridae
9427	Plasmaviridae
9428	Siphoviridae
9429	Myoviridae
9430	Myoviridae
9431	Siphoviridae
9432	Siphoviridae
9433	Siphoviridae

dLGT_phageID	ICTV Phage taxa
9434	Myoviridae
9435	Myoviridae
9436	Siphoviridae
9437	Siphoviridae
9438	Myoviridae
9439	Podoviridae
9440	Inoviridae
9441	Unknown
9442	Unknown
9443	Unknown
9444	Myoviridae
9445	Siphoviridae
9446	Siphoviridae
9447	Unknown
9448	Siphoviridae
9449	Siphoviridae
9450	Siphoviridae
9451	Unknown
9452	Siphoviridae
9453	Siphoviridae
9454	Unknown
9455	Myoviridae
9456	Siphoviridae
9457	Siphoviridae
9458	Unknown
9459	Unknown
9460	Siphoviridae
9461	Unknown
9462	Siphoviridae
9463	Unknown
9464	Siphoviridae
9465	Siphoviridae
9466	Myoviridae
9467	Siphoviridae
9468	Siphoviridae
9469	Siphoviridae
9470	Myoviridae
9471	Unknown
9472	Siphoviridae
9473	Unknown
9474	Siphoviridae
9475	Unknown
9476	Myoviridae
9477	Siphoviridae
9478	Siphoviridae

dLGT_phageID	ICTV Phage taxa
9479	Myoviridae
9480	Unknown
9481	Siphoviridae
9482	Siphoviridae
9483	Unknown
9484	Unknown
9485	Siphoviridae
9486	Unknown
9487	Tectiviridae
9488	Unknown
9489	Siphoviridae
9490	Unknown
9491	Unknown
9492	Siphoviridae
9493	Siphoviridae
9494	Unknown
9495	Unknown
9496	Myoviridae
9497	Myoviridae
9498	Unknown
9499	Unknown
9500	Unknown
9501	Unknown
9502	Siphoviridae
9503	Siphoviridae
9504	Unknown
9505	Siphoviridae
9506	Unknown
9507	Siphoviridae
9508	Myoviridae
9509	Siphoviridae
9510	Unknown
9511	Myoviridae
9512	Unknown
9513	Siphoviridae
9514	Unknown
9515	Siphoviridae
9516	Siphoviridae
9517	Unknown
9518	Siphoviridae
9519	Siphoviridae
9520	Unknown
9521	Siphoviridae
9522	Unknown
9523	Siphoviridae

dLGT_phageID	ICTV Phage taxa
9524	Siphoviridae
9525	Siphoviridae
9526	Unknown
9527	Unknown
9528	Unknown
9529	Unknown
9530	Siphoviridae
9531	Siphoviridae
9532	Unknown
9533	Siphoviridae
9534	Unknown
9535	Unknown
9536	Siphoviridae
9537	Myoviridae
9538	Unknown
9539	Unknown
9540	Unknown
9541	Unknown
9542	Siphoviridae
9543	Myoviridae
9544	Siphoviridae
9545	Unknown
9546	Unknown
9547	Unknown
9548	Siphoviridae
9549	Unknown
9550	Myoviridae
9551	Unknown
9552	Unknown
9553	Myoviridae
9554	Myoviridae
9555	Siphoviridae
9556	Unknown
9557	Myoviridae
9558	Siphoviridae
9559	Siphoviridae
9560	Siphoviridae
9561	Siphoviridae
9562	Siphoviridae
9563	Siphoviridae
9564	Siphoviridae
9565	Siphoviridae
9566	Unknown
9567	Unknown
9568	Unknown

dLGT_phageID	ICTV Phage taxa
9569	Siphoviridae
9570	Siphoviridae
9571	Unknown
9572	Unknown
9573	Unknown
9574	Unknown
9575	Siphoviridae
9576	Unknown
9577	Unknown
9578	Myoviridae
9579	Siphoviridae
9580	Siphoviridae
9581	Unknown
9582	Siphoviridae
9583	Siphoviridae
9584	Unknown
9585	Myoviridae
9586	Siphoviridae
9587	Myoviridae
9588	Siphoviridae
9589	Unknown
9590	Unknown
9591	Siphoviridae
9592	Siphoviridae
9593	Myoviridae
9594	Siphoviridae
9595	Siphoviridae
9596	Siphoviridae
9597	Unknown
9598	Siphoviridae
9599	Siphoviridae
9600	Siphoviridae
9601	Myoviridae
9602	Inoviridae
9603	Unknown
9604	Myoviridae
9605	Myoviridae
9606	Podoviridae
9607	Siphoviridae
9608	Podoviridae
9609	Myoviridae
9610	Siphoviridae
9611	Podoviridae
9612	Siphoviridae
9613	Podoviridae

dLGT_phageID	ICTV Phage taxa
9614	Podoviridae
9615	Unknown
9616	Siphoviridae
9617	Podoviridae
9618	Podoviridae
9619	Myoviridae
9620	Myoviridae
9621	Myoviridae
9622	Siphoviridae
9623	Siphoviridae
9624	Siphoviridae
9625	Siphoviridae
9626	Siphoviridae
9627	Myoviridae
9628	Siphoviridae
9629	Siphoviridae
9630	Unknown
9631	Siphoviridae
9632	Siphoviridae
9633	Myoviridae
9634	Myoviridae
9635	Siphoviridae
9636	Siphoviridae
9637	Siphoviridae
9638	Siphoviridae
9639	Myoviridae
9640	Myoviridae
9641	Unknown
9642	Siphoviridae
9643	Siphoviridae
9644	Siphoviridae
9645	Unknown
9646	Siphoviridae
9647	Siphoviridae
9648	Siphoviridae
9649	Siphoviridae
9650	Unknown
9651	Siphoviridae
9652	Siphoviridae
9653	Unknown
9654	Myoviridae
9655	Siphoviridae
9656	Siphoviridae
9657	Myoviridae
9658	Siphoviridae

dLGT_phageID	ICTV Phage taxa
9659	Siphoviridae
9660	Siphoviridae
9661	Siphoviridae
9662	Siphoviridae
9663	Siphoviridae
9664	Siphoviridae
9665	Unknown
9666	Unknown
9667	Siphoviridae
9668	Siphoviridae
9669	Myoviridae
9670	Unknown
9671	Podoviridae
9672	Myoviridae
9673	Siphoviridae
9674	Myoviridae
9675	Siphoviridae
9676	Siphoviridae
9677	Siphoviridae
9678	Myoviridae
9679	Myoviridae
9680	Siphoviridae
9681	Siphoviridae
9682	Siphoviridae
9683	Podoviridae
9684	Podoviridae
9685	Siphoviridae
9686	Myoviridae
9687	Siphoviridae
9688	Siphoviridae
9689	Siphoviridae
9690	Siphoviridae
9691	Siphoviridae
9692	Unknown
9693	Siphoviridae
9694	Unknown
9695	Unknown
9696	Siphoviridae
9697	Siphoviridae
9698	Siphoviridae
9699	Myoviridae
9700	Siphoviridae
9701	Siphoviridae
9702	Myoviridae
9703	Myoviridae

dLGT_phageID	ICTV Phage taxa
9704	Siphoviridae
9705	Unknown
9706	Siphoviridae
9707	Siphoviridae
9708	Siphoviridae
9709	Myoviridae
9710	Myoviridae
9711	Unknown
9712	Myoviridae
9713	Siphoviridae
9714	Myoviridae
9715	Siphoviridae
9716	Siphoviridae
9717	Siphoviridae
9718	Myoviridae
9719	Unknown
9720	Siphoviridae
9721	Myoviridae
9722	Myoviridae
9723	Myoviridae
9724	Podoviridae
9725	Podoviridae
9726	Siphoviridae
9727	Siphoviridae
9728	Siphoviridae
9729	Myoviridae
9730	Podoviridae
9731	Siphoviridae
9732	Unknown
9733	Unknown
9734	Myoviridae
9735	Siphoviridae
9736	Siphoviridae
9737	Podoviridae
9738	Siphoviridae
9739	Siphoviridae
9740	Myoviridae
9741	Myoviridae
9742	Myoviridae
9743	Myoviridae
9744	Myoviridae
9745	Siphoviridae
9746	Podoviridae
9747	Unknown
9748	Myoviridae

dLGT_phageID	ICTV Phage taxa
9749	Siphoviridae
9750	Siphoviridae
9751	Myoviridae
9752	Unknown
9753	Myoviridae
9754	Siphoviridae
9755	Siphoviridae
9756	Siphoviridae
9757	Siphoviridae
9758	Siphoviridae
9759	Myoviridae
9760	Myoviridae
9761	Siphoviridae
9762	Siphoviridae
9763	Myoviridae
9764	Siphoviridae
9765	Myoviridae
9766	Podoviridae
9767	Siphoviridae
9768	Siphoviridae
9769	Podoviridae
9770	Siphoviridae
9771	Siphoviridae
9772	Myoviridae
9773	Unknown
9774	Unknown
9775	Siphoviridae
9776	Myoviridae
9777	Siphoviridae
9778	Siphoviridae
9779	Unknown
9780	Myoviridae
9781	Siphoviridae
9782	Unknown
9783	Unknown
9784	Siphoviridae
9785	Siphoviridae
9786	Siphoviridae
9787	Siphoviridae
9788	Siphoviridae
9789	Siphoviridae
9790	Siphoviridae
9791	Myoviridae
9792	Siphoviridae
9793	Siphoviridae

dLGT_phageID	ICTV Phage taxa
9794	Myoviridae
9795	Myoviridae
9796	Myoviridae
9797	Myoviridae
9798	Myoviridae
9799	Siphoviridae
9800	Siphoviridae
9801	Siphoviridae
9802	Unknown
9803	Unknown
9804	Myoviridae
9805	Myoviridae
9806	Siphoviridae
9807	Myoviridae
9808	Siphoviridae
9809	Myoviridae
9810	Siphoviridae
9811	Siphoviridae
9812	Siphoviridae
9813	Siphoviridae
9814	Siphoviridae
9815	Unknown
9816	Myoviridae
9817	Siphoviridae
9818	Siphoviridae
9819	Siphoviridae
9820	Siphoviridae
9821	Myoviridae
9822	Siphoviridae
9823	Siphoviridae
9824	Siphoviridae
9825	Siphoviridae
9826	Siphoviridae
9827	Unknown
9828	Siphoviridae
9829	Siphoviridae
9830	Myoviridae
9831	Siphoviridae
9832	Siphoviridae
9833	Siphoviridae
9834	Unknown
9835	Siphoviridae
9836	Unknown
9837	Siphoviridae
9838	Siphoviridae

dLGT_phageID	ICTV Phage taxa
9839	Siphoviridae
9840	Unknown
9841	Siphoviridae
9842	Siphoviridae
9843	Siphoviridae
9844	Siphoviridae
9845	Myoviridae
9846	Myoviridae
9847	Unknown
9848	Siphoviridae
9849	Myoviridae
9850	Myoviridae
9851	Myoviridae
9852	Unknown
9853	Siphoviridae
9854	Siphoviridae
9855	Unknown
9856	Siphoviridae
9857	Podoviridae
9858	Unknown
9859	Siphoviridae
9860	Siphoviridae
9861	Podoviridae
9862	Myoviridae
9863	Podoviridae
9864	Unknown
9865	Podoviridae
9866	Siphoviridae
9867	Myoviridae
9868	Myoviridae
9869	Unknown
9870	Myoviridae
9871	Myoviridae
9872	Myoviridae
9873	Myoviridae
9874	Siphoviridae
9875	Myoviridae
9876	Myoviridae
9877	Myoviridae
9878	Inoviridae
9879	Podoviridae
9880	Siphoviridae
9881	Siphoviridae
9882	Siphoviridae
9883	Siphoviridae

dLGT_phageID	ICTV Phage taxa
9884	Myoviridae
9885	Siphoviridae
9886	Siphoviridae
9887	Unknown
9888	Siphoviridae
9889	Myoviridae
9890	Myoviridae
9891	Myoviridae
9892	Unknown
9893	Myoviridae
9894	Myoviridae
9895	Siphoviridae
9896	Siphoviridae
9897	Myoviridae
9898	Myoviridae
9899	Myoviridae
9900	Myoviridae
9901	Podoviridae
9902	Siphoviridae
9903	Myoviridae
9904	Myoviridae
9905	Siphoviridae
9906	Siphoviridae
9907	Podoviridae
9908	Siphoviridae
9909	Siphoviridae
9910	Podoviridae
9911	Siphoviridae
9912	Siphoviridae
9913	Siphoviridae
9914	Myoviridae
9915	Inoviridae
9916	Podoviridae
9917	Myoviridae
9918	Podoviridae
9919	Myoviridae
9920	Myoviridae
9921	Myoviridae
9922	Siphoviridae
9923	Siphoviridae
9924	Podoviridae
9925	Siphoviridae
9926	Myoviridae
9927	Unknown
9928	Myoviridae

dLGT_phageID	ICTV Phage taxa
9929	Siphoviridae
9930	Myoviridae
9931	Unknown
9932	Myoviridae
9933	Podoviridae
9934	Unknown
9935	Myoviridae
9936	Unknown
9937	Podoviridae
9938	Siphoviridae
9939	Unknown
9940	Myoviridae
9941	Unknown
9942	Plasmaviridae
9943	Siphoviridae
9944	Siphoviridae
9945	Siphoviridae
9946	Unknown
9947	Siphoviridae
9948	Unknown
9949	Siphoviridae
9950	Siphoviridae
9951	Siphoviridae
9952	Siphoviridae
9953	Myoviridae
9954	Myoviridae
9955	Myoviridae
9956	Unknown
9957	Myoviridae
9958	Myoviridae
9959	Myoviridae
9960	Siphoviridae
9961	Siphoviridae
9962	Siphoviridae
9963	Myoviridae
9964	Myoviridae
9965	Myoviridae
9966	Siphoviridae
9967	Siphoviridae
9968	Siphoviridae
9969	Siphoviridae
9970	Siphoviridae
9971	Siphoviridae
9972	Siphoviridae
9973	Siphoviridae

dLGT_phageID	ICTV Phage taxa
9974	Unknown
9975	Siphoviridae
9976	Siphoviridae
9977	Myoviridae
9978	Podoviridae
9979	Siphoviridae
9980	Siphoviridae
9981	Myoviridae
9982	Siphoviridae
9983	Siphoviridae
9984	Siphoviridae
9985	Siphoviridae
9986	Siphoviridae
9987	Siphoviridae
9988	Myoviridae
9989	Siphoviridae
9990	Siphoviridae
9991	Myoviridae
9992	Podoviridae
9993	Siphoviridae
9994	Podoviridae
9995	Siphoviridae
9996	Siphoviridae
9997	Unknown
9998	Siphoviridae
9999	Siphoviridae
10000	Podoviridae
10001	Siphoviridae
10002	Siphoviridae
10003	Siphoviridae
10004	Plasmaviridae
10005	Siphoviridae
10006	Siphoviridae
10007	Siphoviridae
10008	Myoviridae
10009	Siphoviridae
10010	Siphoviridae
10011	Siphoviridae
10012	Unknown
10013	Podoviridae
10014	Siphoviridae
10015	Siphoviridae
10016	Podoviridae
10017	Siphoviridae
10018	Siphoviridae

dLGT_phageID	ICTV Phage taxa
10019	Myoviridae
10020	Siphoviridae
10021	Myoviridae
10022	Siphoviridae
10023	Siphoviridae
10024	Siphoviridae
10025	Unknown
10026	Siphoviridae
10027	Siphoviridae
10028	Siphoviridae
10029	Siphoviridae
10030	Myoviridae
10031	Siphoviridae
10032	Myoviridae
10033	Siphoviridae
10034	Myoviridae
10035	Siphoviridae
10036	Myoviridae
10037	Unknown
10038	Myoviridae
10039	Myoviridae
10040	Siphoviridae
10041	Myoviridae
10042	Myoviridae
10043	Podoviridae
10044	Siphoviridae
10045	Siphoviridae
10046	Podoviridae
10047	Siphoviridae
10048	Siphoviridae
10049	Siphoviridae
10050	Siphoviridae
10051	Myoviridae
10052	Siphoviridae
10053	Myoviridae
10054	Siphoviridae
10055	Myoviridae
10056	Myoviridae
10057	Podoviridae
10058	Siphoviridae
10059	Siphoviridae
10060	Myoviridae
10061	Siphoviridae
10062	Myoviridae
10063	Siphoviridae

dLGT_phageID	ICTV Phage taxa
10064	Podoviridae
10065	Myoviridae
10066	Unknown
10067	Myoviridae
10068	Siphoviridae
10069	Siphoviridae
10070	Inoviridae
10071	Siphoviridae
10072	Plasmaviridae
10073	Myoviridae
10074	Siphoviridae
10075	Siphoviridae
10076	Unknown
10077	Myoviridae
10078	Podoviridae
10079	Siphoviridae
10080	Siphoviridae
10081	Podoviridae
10082	Myoviridae
10083	Unknown
10084	Siphoviridae
10085	Podoviridae
10086	Myoviridae
10087	Myoviridae
10088	Myoviridae
10089	Siphoviridae
10090	Siphoviridae
10091	Podoviridae
10092	Myoviridae
10093	Myoviridae
10094	Siphoviridae
10095	Unknown
10096	Inoviridae
10097	Siphoviridae
10098	Siphoviridae
10099	Tectiviridae
10100	Siphoviridae
10101	Siphoviridae
10102	Siphoviridae
10103	Myoviridae
10104	Siphoviridae
10105	Siphoviridae
10106	Siphoviridae
10107	Myoviridae
10108	Siphoviridae

dLGT_phageID	ICTV Phage taxa
10109	Siphoviridae
10110	Myoviridae
10111	Siphoviridae
10112	Myoviridae
10113	Myoviridae
10114	Myoviridae
10115	Myoviridae
10116	Myoviridae
10117	Siphoviridae
10118	Siphoviridae
10119	Siphoviridae
10120	Unknown
10121	Siphoviridae
10122	Siphoviridae
10123	Siphoviridae
10124	Myoviridae
10125	Siphoviridae
10126	Myoviridae
10127	Unknown
10128	Unknown
10129	Siphoviridae
10130	Myoviridae
10131	Siphoviridae
10132	Siphoviridae
10133	Myoviridae
10134	Siphoviridae
10135	Myoviridae
10136	Siphoviridae
10137	Siphoviridae
10138	Siphoviridae
10139	Siphoviridae
10140	Podoviridae
10141	Siphoviridae
10142	Unknown
10143	Myoviridae
10144	Siphoviridae
10145	Unknown
10146	Siphoviridae
10147	Siphoviridae
10148	Unknown
10149	Siphoviridae
10150	Siphoviridae
10151	Siphoviridae
10152	Siphoviridae
10153	Siphoviridae

dLGT_phageID	ICTV Phage taxa
10154	Myoviridae
10155	Siphoviridae
10156	Siphoviridae
10157	Myoviridae
10158	Siphoviridae
10159	Podoviridae
10160	Podoviridae
10161	Siphoviridae
10162	Myoviridae
10163	Myoviridae
10164	Myoviridae
10165	Siphoviridae
10166	Myoviridae
10167	Siphoviridae
10168	Myoviridae
10169	Siphoviridae
10170	Unknown
10171	Unknown
10172	Siphoviridae
10173	Siphoviridae
10174	Myoviridae
10175	Unknown
10176	Myoviridae
10177	Myoviridae
10178	Siphoviridae
10179	Unknown
10180	Siphoviridae
10181	Siphoviridae
10182	Myoviridae
10183	Siphoviridae
10184	Siphoviridae
10185	Siphoviridae
10186	Siphoviridae
10187	Siphoviridae
10188	Siphoviridae
10189	Siphoviridae
10190	Podoviridae
10191	Myoviridae
10192	Siphoviridae
10193	Siphoviridae
10194	Siphoviridae
10195	Myoviridae
10196	Unknown
10197	Podoviridae
10198	Myoviridae

dLGT_phageID	ICTV Phage taxa
10199	Siphoviridae
10200	Unknown
10201	Myoviridae
10202	Siphoviridae
10203	Myoviridae
10204	Siphoviridae
10205	Siphoviridae
10206	Unknown
10207	Siphoviridae
10208	Myoviridae
10209	Siphoviridae
10210	Podoviridae
10211	Siphoviridae
10212	Myoviridae
10213	Unknown
10214	Siphoviridae
10215	Myoviridae
10216	Siphoviridae
10217	Myoviridae
10218	Inoviridae
10219	Siphoviridae
10220	Siphoviridae
10221	Podoviridae
10222	Podoviridae
10223	Myoviridae
10224	Myoviridae
10225	Podoviridae
10226	Myoviridae
10227	Myoviridae
10228	Myoviridae
10229	Siphoviridae
10230	Podoviridae
10231	Podoviridae
10232	Myoviridae
10233	Siphoviridae
10234	Siphoviridae
10235	Myoviridae
10236	Myoviridae
10237	Siphoviridae
10238	Siphoviridae
10239	Siphoviridae
10240	Siphoviridae
10241	Siphoviridae
10242	Myoviridae
10243	Siphoviridae

dLGT_phageID	ICTV Phage taxa
10244	Podoviridae
10245	Myoviridae
10246	Myoviridae
10247	Siphoviridae
10248	Podoviridae
10249	Siphoviridae
10250	Siphoviridae
10251	Siphoviridae
10252	Siphoviridae
10253	Siphoviridae
10254	Siphoviridae
10255	Myoviridae
10256	Siphoviridae
10257	Siphoviridae
10258	Myoviridae
10259	Myoviridae
10260	Myoviridae
10261	Myoviridae
10262	Unknown
10263	Siphoviridae
10264	Siphoviridae
10265	Myoviridae
10266	Siphoviridae
10267	Myoviridae
10268	Podoviridae
10269	Siphoviridae
10270	Siphoviridae
10271	Myoviridae
10272	Siphoviridae
10273	Siphoviridae
10274	Myoviridae
10275	Myoviridae
10276	Siphoviridae
10277	Myoviridae
10278	Siphoviridae
10279	Siphoviridae
10280	Myoviridae
10281	Siphoviridae
10282	Siphoviridae
10283	Myoviridae
10284	Siphoviridae
10285	Siphoviridae
10286	Myoviridae
10287	Siphoviridae
10288	Myoviridae

dLGT_phageID	ICTV Phage taxa
10289	Siphoviridae
10290	Unknown
10291	Siphoviridae
10292	Siphoviridae
10293	Siphoviridae
10294	Siphoviridae
10295	Podoviridae
10296	Siphoviridae
10297	Unknown
10298	Siphoviridae
10299	Siphoviridae
10300	Podoviridae
10301	Siphoviridae
10302	Unknown
10303	Siphoviridae
10304	Siphoviridae
10305	Siphoviridae
10306	Unknown
10307	Myoviridae
10308	Siphoviridae
10309	Siphoviridae
10310	Siphoviridae
10311	Siphoviridae
10312	Myoviridae
10313	Podoviridae
10314	Siphoviridae
10315	Myoviridae
10316	Siphoviridae
10317	Siphoviridae
10318	Siphoviridae
10319	Myoviridae
10320	Siphoviridae
10321	Myoviridae
10322	Siphoviridae
10323	Siphoviridae
10324	Unknown
10325	Siphoviridae
10326	Siphoviridae
10327	Siphoviridae
10328	Siphoviridae
10329	Myoviridae
10330	Siphoviridae
10331	Myoviridae
10332	Siphoviridae
10333	Siphoviridae

dLGT_phageID	ICTV Phage taxa
10334	Siphoviridae
10335	Myoviridae
10336	Siphoviridae
10337	Siphoviridae
10338	Siphoviridae
10339	Siphoviridae
10340	Siphoviridae
10341	Myoviridae
10342	Siphoviridae
10343	Siphoviridae
10344	Siphoviridae
10345	Siphoviridae
10346	Myoviridae
10347	Myoviridae
10348	Siphoviridae
10349	Siphoviridae
10350	Siphoviridae
10351	Siphoviridae
10352	Myoviridae
10353	Myoviridae
10354	Myoviridae
10355	Podoviridae
10356	Myoviridae
10357	Myoviridae
10358	Siphoviridae
10359	Myoviridae
10360	Myoviridae
10361	Siphoviridae
10362	Siphoviridae
10363	Siphoviridae
10364	Podoviridae
10365	Siphoviridae
10366	Siphoviridae
10367	Siphoviridae
10368	Siphoviridae
10369	Siphoviridae
10370	Myoviridae
10371	Siphoviridae
10372	Myoviridae
10373	Siphoviridae
10374	Unknown
10375	Siphoviridae
10376	Siphoviridae
10377	Myoviridae
10378	Siphoviridae

dLGT_phageID	ICTV Phage taxa
10379	Siphoviridae
10380	Myoviridae
10381	Siphoviridae
10382	Siphoviridae
10383	Siphoviridae
10384	Siphoviridae
10385	Siphoviridae
10386	Unknown
10387	Siphoviridae
10388	Myoviridae
10389	Siphoviridae
10390	Unknown
10391	Myoviridae
10392	Siphoviridae
10393	Podoviridae
10394	Unknown
10395	Siphoviridae
10396	Siphoviridae
10397	Siphoviridae
10398	Siphoviridae
10399	Siphoviridae
10400	Myoviridae
10401	Unknown
10402	Siphoviridae
10403	Myoviridae
10404	Unknown
10405	Podoviridae
10406	Podoviridae
10407	Siphoviridae
10408	Siphoviridae
10409	Podoviridae
10410	Siphoviridae
10411	Myoviridae
10412	Myoviridae
10413	Myoviridae
10414	Siphoviridae
10415	Siphoviridae
10416	Myoviridae
10417	Siphoviridae
10418	Siphoviridae
10419	Siphoviridae
10420	Siphoviridae
10421	Siphoviridae
10422	Siphoviridae
10423	Siphoviridae

dLGT_phageID	ICTV Phage taxa
10424	Siphoviridae
10425	Siphoviridae
10426	Siphoviridae
10427	Siphoviridae
10428	Myoviridae
10429	Siphoviridae
10430	Myoviridae
10431	Siphoviridae
10432	Siphoviridae
10433	Siphoviridae
10434	Siphoviridae
10435	Siphoviridae
10436	Siphoviridae
10437	Siphoviridae
10438	Unknown
10439	Siphoviridae
10440	Unknown
10441	Siphoviridae
10442	Unknown
10443	Unknown
10444	Siphoviridae
10445	Unknown
10446	Siphoviridae
10447	Siphoviridae
10448	Myoviridae
10449	Myoviridae
10450	Unknown
10451	Siphoviridae
10452	Unknown
10453	Siphoviridae
10454	Siphoviridae
10455	Myoviridae
10456	Siphoviridae
10457	Myoviridae
10458	Siphoviridae
10459	Siphoviridae
10460	Siphoviridae
10461	Myoviridae
10462	Siphoviridae
10463	Siphoviridae
10464	Myoviridae
10465	Siphoviridae
10466	Siphoviridae
10467	Siphoviridae
10468	Siphoviridae

dLGT_phageID	ICTV Phage taxa
10469	Siphoviridae
10470	Siphoviridae
10471	Siphoviridae
10472	Siphoviridae
10473	Myoviridae
10474	Myoviridae
10475	Siphoviridae
10476	Siphoviridae
10477	Siphoviridae
10478	Siphoviridae
10479	Siphoviridae
10480	Siphoviridae
10481	Siphoviridae
10482	Myoviridae
10483	Siphoviridae
10484	Siphoviridae
10485	Siphoviridae
10486	Siphoviridae
10487	Siphoviridae
10488	Siphoviridae
10489	Unknown
10490	Siphoviridae
10491	Unknown
10492	Siphoviridae
10493	Siphoviridae
10494	Siphoviridae
10495	Siphoviridae
10496	Siphoviridae
10497	Siphoviridae
10498	Myoviridae
10499	Siphoviridae
10500	Siphoviridae
10501	Siphoviridae
10502	Siphoviridae
10503	Siphoviridae
10504	Siphoviridae
10505	Siphoviridae
10506	Siphoviridae
10507	Unknown
10508	Siphoviridae
10509	Myoviridae
10510	Siphoviridae
10511	Myoviridae
10512	Siphoviridae
10513	Siphoviridae

dLGT_phageID	ICTV Phage taxa
10514	Myoviridae
10515	Siphoviridae
10516	Siphoviridae
10517	Unknown
10518	Myoviridae
10519	Siphoviridae
10520	Siphoviridae
10521	Siphoviridae
10522	Siphoviridae
10523	Siphoviridae
10524	Siphoviridae
10525	Siphoviridae
10526	Myoviridae
10527	Siphoviridae
10528	Siphoviridae
10529	Myoviridae
10530	Siphoviridae
10531	Siphoviridae
10532	Unknown
10533	Siphoviridae
10534	Siphoviridae
10535	Siphoviridae
10536	Unknown
10537	Siphoviridae
10538	Myoviridae
10539	Myoviridae
10540	Siphoviridae
10541	Siphoviridae
10542	Myoviridae
10543	Siphoviridae
10544	Siphoviridae
10545	Siphoviridae
10546	Myoviridae
10547	Podoviridae
10548	Myoviridae
10549	Myoviridae
10550	Siphoviridae
10551	Myoviridae
10552	Myoviridae
10553	Siphoviridae
10554	Myoviridae
10555	Myoviridae
10556	Myoviridae
10557	Siphoviridae
10558	Myoviridae

dLGT_phageID	ICTV Phage taxa
10559	Myoviridae
10560	Siphoviridae
10561	Unknown
10562	Myoviridae
10563	Siphoviridae
10564	Unknown
10565	Siphoviridae
10566	Unknown
10567	Myoviridae
10568	Siphoviridae
10569	Unknown
10570	Siphoviridae
10571	Unknown
10572	Podoviridae
10573	Siphoviridae
10574	Unknown
10575	Siphoviridae
10576	Podoviridae
10577	Siphoviridae
10578	Myoviridae
10579	Siphoviridae
10580	Unknown
10581	Siphoviridae
10582	Unknown
10583	Myoviridae
10584	Unknown
10585	Siphoviridae
10586	Unknown
10587	Siphoviridae
10588	Siphoviridae
10589	Siphoviridae
10590	Siphoviridae
10591	Unknown
10592	Siphoviridae
10593	Unknown
10594	Podoviridae
10595	Siphoviridae
10596	Siphoviridae
10597	Myoviridae
10598	Siphoviridae
10599	Tectiviridae
10600	Siphoviridae
10601	Siphoviridae
10602	Siphoviridae
10603	Siphoviridae

dLGT_phageID	ICTV Phage taxa
10604	Unknown
10605	Unknown
10606	Siphoviridae
10607	Unknown
10608	Siphoviridae
10609	Siphoviridae
10610	Siphoviridae
10611	Siphoviridae
10612	Siphoviridae
10613	Siphoviridae
10614	Siphoviridae
10615	Unknown
10616	Siphoviridae
10617	Siphoviridae
10618	Siphoviridae
10619	Siphoviridae
10620	Siphoviridae
10621	Myoviridae
10622	Siphoviridae
10623	Unknown
10624	Siphoviridae
10625	Siphoviridae
10626	Siphoviridae
10627	Unknown
10628	Siphoviridae
10629	Siphoviridae
10630	Myoviridae
10631	Siphoviridae
10632	Podoviridae
10633	Siphoviridae
10634	Siphoviridae
10635	Unknown
10636	Myoviridae
10637	Unknown
10638	Siphoviridae
10639	Siphoviridae
10640	Unknown
10641	Myoviridae
10642	Siphoviridae
10643	Myoviridae
10644	Unknown
10645	Myoviridae
10646	Siphoviridae
10647	Siphoviridae
10648	Siphoviridae

dLGT_phageID	ICTV Phage taxa
10649	Siphoviridae
10650	Myoviridae
10651	Myoviridae
10652	Myoviridae
10653	Siphoviridae
10654	Siphoviridae
10655	Myoviridae
10656	Myoviridae
10657	Siphoviridae
10658	Siphoviridae
10659	Siphoviridae
10660	Siphoviridae
10661	Unknown
10662	Siphoviridae
10663	Siphoviridae
10664	Siphoviridae
10665	Siphoviridae
10666	Siphoviridae
10667	Myoviridae
10668	Siphoviridae
10669	Myoviridae
10670	Myoviridae
10671	Siphoviridae
10672	Siphoviridae
10673	Siphoviridae
10674	Siphoviridae
10675	Siphoviridae
10676	Myoviridae
10677	Podoviridae
10678	Siphoviridae
10679	Siphoviridae
10680	Siphoviridae
10681	Siphoviridae
10682	Siphoviridae
10683	Siphoviridae
10684	Siphoviridae
10685	Siphoviridae
10686	Siphoviridae
10687	Unknown
10688	Siphoviridae
10689	Siphoviridae
10690	Siphoviridae
10691	Unknown
10692	Siphoviridae
10693	Podoviridae

dLGT_phageID	ICTV Phage taxa
10694	Siphoviridae
10695	Myoviridae
10696	Podoviridae
10697	Siphoviridae
10698	Siphoviridae
10699	Unknown
10700	Siphoviridae
10701	Myoviridae
10702	Myoviridae
10703	Myoviridae
10704	Myoviridae
10705	Podoviridae
10706	Siphoviridae
10707	Siphoviridae
10708	Podoviridae
10709	Unknown
10710	Podoviridae
10711	Podoviridae
10712	Siphoviridae
10713	Siphoviridae
10714	Siphoviridae
10715	Podoviridae
10716	Unknown
10717	Siphoviridae
10718	Siphoviridae
10719	Myoviridae
10720	Podoviridae
10721	Podoviridae
10722	Siphoviridae
10723	Siphoviridae
10724	Siphoviridae
10725	Myoviridae
10726	Siphoviridae
10727	Siphoviridae
10728	Unknown
10729	Myoviridae
10730	Unknown
10731	Siphoviridae
10732	Siphoviridae
10733	Myoviridae
10734	Myoviridae
10735	Myoviridae
10736	Siphoviridae
10737	Myoviridae
10738	Myoviridae

dLGT_phageID	ICTV Phage taxa
10739	Siphoviridae
10740	Siphoviridae
10741	Unknown
10742	Siphoviridae
10743	Myoviridae
10744	Siphoviridae
10745	Myoviridae
10746	Unknown
10747	Myoviridae
10748	Siphoviridae
10749	Podoviridae
10750	Siphoviridae
10751	Siphoviridae
10752	Siphoviridae
10753	Siphoviridae
10754	Siphoviridae
10755	Myoviridae
10756	Siphoviridae
10757	Myoviridae
10758	Siphoviridae
10759	Myoviridae
10760	Myoviridae
10761	Siphoviridae
10762	Unknown
10763	Myoviridae
10764	Siphoviridae
10765	Siphoviridae
10766	Myoviridae
10767	Siphoviridae
10768	Podoviridae
10769	Siphoviridae
10770	Podoviridae
10771	Unknown
10772	Siphoviridae
10773	Siphoviridae
10774	Unknown
10775	Myoviridae
10776	Myoviridae
10777	Myoviridae
10778	Siphoviridae
10779	Siphoviridae
10780	Siphoviridae
10781	Podoviridae
10782	Inoviridae
10783	Siphoviridae

dLGT_phageID	ICTV Phage taxa
10784	Unknown
10785	Siphoviridae
10786	Siphoviridae
10787	Myoviridae
10788	Siphoviridae
10789	Siphoviridae
10790	Siphoviridae
10791	Myoviridae
10792	Myoviridae
10793	Siphoviridae
10794	Myoviridae
10795	Siphoviridae
10796	Siphoviridae
10797	Siphoviridae
10798	Myoviridae
10799	Siphoviridae
10800	Tectiviridae
10801	Myoviridae
10802	Myoviridae
10803	Siphoviridae
10804	Podoviridae
10805	Myoviridae
10806	Siphoviridae
10807	Myoviridae
10808	Siphoviridae
10809	Myoviridae
10810	Siphoviridae
10811	Siphoviridae
10812	Unknown
10813	Siphoviridae
10814	Siphoviridae
10815	Siphoviridae
10816	Unknown
10817	Siphoviridae
10818	Siphoviridae
10819	Myoviridae
10820	Siphoviridae
10821	Siphoviridae
10822	Siphoviridae
10823	Myoviridae
10824	Myoviridae
10825	Myoviridae
10826	Siphoviridae
10827	Siphoviridae
10828	Siphoviridae

dLGT_phageID	ICTV Phage taxa
10829	Unknown
10830	Myoviridae
10831	Siphoviridae
10832	Siphoviridae
10833	Myoviridae
10834	Siphoviridae
10835	Siphoviridae
10836	Siphoviridae
10837	Myoviridae
10838	Myoviridae
10839	Unknown
10840	Inoviridae
10841	Siphoviridae
10842	Inoviridae
10843	Siphoviridae
10844	Siphoviridae
10845	Podoviridae
10846	Siphoviridae
10847	Siphoviridae
10848	Unknown
10849	Myoviridae
10850	Siphoviridae
10851	Siphoviridae
10852	Siphoviridae
10853	Myoviridae
10854	Myoviridae
10855	Myoviridae
10856	Siphoviridae
10857	Inoviridae
10858	Siphoviridae
10859	Siphoviridae
10860	Siphoviridae
10861	Siphoviridae
10862	Unknown
10863	Siphoviridae
10864	Myoviridae
10865	Siphoviridae
10866	Siphoviridae
10867	Myoviridae
10868	Siphoviridae
10869	Siphoviridae
10870	Podoviridae
10871	Podoviridae
10872	Siphoviridae
10873	Podoviridae

dLGT_phageID	ICTV Phage taxa
10874	Myoviridae
10875	Unknown
10876	Myoviridae
10877	Siphoviridae
10878	Siphoviridae
10879	Siphoviridae
10880	Siphoviridae
10881	Siphoviridae
10882	Myoviridae
10883	Myoviridae
10884	Podoviridae
10885	Myoviridae
10886	Siphoviridae
10887	Siphoviridae
10888	Siphoviridae
10889	Unknown
10890	Podoviridae
10891	Myoviridae
10892	Myoviridae
10893	Siphoviridae
10894	Siphoviridae
10895	Siphoviridae
10896	Myoviridae
10897	Unknown
10898	Siphoviridae
10899	Siphoviridae
10900	Siphoviridae
10901	Siphoviridae
10902	Myoviridae
10903	Siphoviridae
10904	Podoviridae
10905	Siphoviridae
10906	Siphoviridae
10907	Unknown
10908	Myoviridae
10909	Podoviridae
10910	Siphoviridae
10911	Siphoviridae
10912	Myoviridae
10913	Siphoviridae
10914	Myoviridae
10915	Siphoviridae
10916	Myoviridae
10917	Myoviridae
10918	Siphoviridae

dLGT_phageID	ICTV Phage taxa
10919	Siphoviridae
10920	Siphoviridae
10921	Siphoviridae
10922	Siphoviridae
10923	Myoviridae
10924	Unknown
10925	Myoviridae
10926	Unknown
10927	Myoviridae
10928	Siphoviridae
10929	Siphoviridae
10930	Unknown
10931	Myoviridae
10932	Siphoviridae
10933	Siphoviridae
10934	Siphoviridae
10935	Siphoviridae
10936	Unknown
10937	Siphoviridae
10938	Myoviridae
10939	Myoviridae
10940	Myoviridae
10941	Unknown
10942	Myoviridae
10943	Siphoviridae
10944	Unknown
10945	Myoviridae
10946	Myoviridae
10947	Siphoviridae
10948	Siphoviridae
10949	Myoviridae
10950	Myoviridae
10951	Siphoviridae
10952	Siphoviridae
10953	Siphoviridae
10954	Plasmaviridae
10955	Siphoviridae
10956	Myoviridae
10957	Myoviridae
10958	Siphoviridae
10959	Siphoviridae
10960	Siphoviridae
10961	Siphoviridae
10962	Myoviridae
10963	Myoviridae

dLGT_phageID	ICTV Phage taxa
10964	Siphoviridae
10965	Siphoviridae
10966	Siphoviridae
10967	Myoviridae
10968	Siphoviridae
10969	Siphoviridae
10970	Myoviridae
10971	Siphoviridae
10972	Unknown
10973	Myoviridae
10974	Myoviridae
10975	Myoviridae
10976	Unknown
10977	Siphoviridae
10978	Myoviridae
10979	Siphoviridae
10980	Myoviridae
10981	Unknown
10982	Unknown
10983	Podoviridae
10984	Siphoviridae
10985	Myoviridae
10986	Siphoviridae
10987	Siphoviridae
10988	Siphoviridae
10989	Siphoviridae
10990	Podoviridae
10991	Myoviridae
10992	Siphoviridae
10993	Siphoviridae
10994	Podoviridae
10995	Myoviridae
10996	Siphoviridae
10997	Myoviridae
10998	Unknown
10999	Siphoviridae
11000	Myoviridae
11001	Siphoviridae
11002	Myoviridae
11003	Inoviridae
11004	Myoviridae
11005	Siphoviridae
11006	Myoviridae
11007	Siphoviridae
11008	Siphoviridae

dLGT_phageID	ICTV Phage taxa
11009	Siphoviridae
11010	Myoviridae
11011	Siphoviridae
11012	Siphoviridae
11013	Unknown
11014	Myoviridae
11015	Siphoviridae
11016	Podoviridae
11017	Siphoviridae
11018	Siphoviridae
11019	Siphoviridae
11020	Myoviridae
11021	Myoviridae
11022	Myoviridae
11023	Podoviridae
11024	Siphoviridae
11025	Podoviridae
11026	Inoviridae
11027	Siphoviridae
11028	Unknown
11029	Siphoviridae
11030	Siphoviridae
11031	Myoviridae
11032	Siphoviridae
11033	Siphoviridae
11034	Siphoviridae
11035	Unknown
11036	Siphoviridae
11037	Siphoviridae
11038	Siphoviridae
11039	Siphoviridae
11040	Unknown
11041	Siphoviridae
11042	Myoviridae
11043	Siphoviridae
11044	Myoviridae
11045	Unknown
11046	Siphoviridae
11047	Siphoviridae
11048	Myoviridae
11049	Unknown
11050	Siphoviridae
11051	Siphoviridae
11052	Siphoviridae
11053	Siphoviridae

dLGT_phageID	ICTV Phage taxa
11054	Myoviridae
11055	Myoviridae
11056	Podoviridae
11057	Myoviridae
11058	Unknown
11059	Myoviridae
11060	Myoviridae
11061	Siphoviridae
11062	Myoviridae
11063	Siphoviridae
11064	Siphoviridae
11065	Siphoviridae
11066	Myoviridae
11067	Siphoviridae
11068	Myoviridae
11069	Siphoviridae
11070	Siphoviridae
11071	Podoviridae
11072	Myoviridae
11073	Unknown
11074	Myoviridae
11075	Myoviridae
11076	Siphoviridae
11077	Siphoviridae
11078	Siphoviridae
11079	Siphoviridae
11080	Siphoviridae
11081	Myoviridae
11082	Myoviridae
11083	Myoviridae
11084	Siphoviridae
11085	Podoviridae
11086	Siphoviridae
11087	Siphoviridae
11088	Siphoviridae
11089	Podoviridae
11090	Podoviridae
11091	Siphoviridae
11092	Unknown
11093	Siphoviridae
11094	Siphoviridae
11095	Siphoviridae
11096	Siphoviridae
11097	Myoviridae
11098	Myoviridae

dLGT_phageID	ICTV Phage taxa
11099	Myoviridae
11100	Podoviridae
11101	Siphoviridae
11102	Siphoviridae
11103	Myoviridae
11104	Siphoviridae
11105	Siphoviridae
11106	Siphoviridae
11107	Siphoviridae
11108	Podoviridae
11109	Myoviridae
11110	Myoviridae
11111	Siphoviridae
11112	Siphoviridae
11113	Podoviridae
11114	Siphoviridae
11115	Myoviridae
11116	Siphoviridae
11117	Siphoviridae
11118	Siphoviridae
11119	Siphoviridae
11120	Unknown
11121	Siphoviridae
11122	Unknown
11123	Unknown
11124	Myoviridae
11125	Podoviridae
11126	Siphoviridae
11127	Myoviridae
11128	Siphoviridae
11129	Myoviridae
11130	Unknown
11131	Siphoviridae
11132	Siphoviridae
11133	Siphoviridae
11134	Siphoviridae
11135	Siphoviridae
11136	Siphoviridae
11137	Siphoviridae
11138	Myoviridae
11139	Podoviridae
11140	Siphoviridae
11141	Podoviridae
11142	Myoviridae
11143	Podoviridae

dLGT_phageID	ICTV Phage taxa
11144	Inoviridae
11145	Unknown
11146	Myoviridae
11147	Podoviridae
11148	Siphoviridae
11149	Myoviridae
11150	Siphoviridae
11151	Siphoviridae
11152	Siphoviridae
11153	Myoviridae
11154	Siphoviridae
11155	Siphoviridae
11156	Siphoviridae
11157	Siphoviridae
11158	Unknown
11159	Siphoviridae
11160	Myoviridae
11161	Unknown
11162	Siphoviridae
11163	Siphoviridae
11164	Myoviridae
11165	Siphoviridae
11166	Podoviridae
11167	Myoviridae
11168	Myoviridae
11169	Siphoviridae
11170	Podoviridae
11171	Siphoviridae
11172	Siphoviridae
11173	Unknown
11174	Unknown
11175	Unknown
11176	Siphoviridae
11177	Unknown
11178	Siphoviridae
11179	Siphoviridae
11180	Myoviridae
11181	Unknown
11182	Siphoviridae
11183	Siphoviridae
11184	Myoviridae
11185	Unknown
11186	Siphoviridae
11187	Siphoviridae
11188	Siphoviridae

dLGT_phageID	ICTV Phage taxa
11189	Podoviridae
11190	Siphoviridae
11191	Unknown
11192	Siphoviridae
11193	Siphoviridae
11194	Myoviridae
11195	Podoviridae
11196	Unknown
11197	Unknown
11198	Siphoviridae
11199	Unknown
11200	Siphoviridae
11201	Unknown
11202	Myoviridae
11203	Siphoviridae
11204	Myoviridae
11205	Siphoviridae
11206	Siphoviridae
11207	Unknown
11208	Siphoviridae
11209	Unknown
11210	Unknown
11211	Siphoviridae
11212	Myoviridae
11213	Unknown
11214	Siphoviridae
11215	Siphoviridae
11216	Myoviridae
11217	Podoviridae
11218	Unknown
11219	Unknown
11220	Myoviridae
11221	Siphoviridae
11222	Siphoviridae
11223	Unknown
11224	Siphoviridae
11225	Podoviridae
11226	Siphoviridae
11227	Myoviridae
11228	Siphoviridae
11229	Siphoviridae
11230	Myoviridae
11231	Unknown
11232	Myoviridae
11233	Myoviridae

dLGT_phageID	ICTV Phage taxa
11234	Siphoviridae
11235	Podoviridae
11236	Myoviridae
11237	Myoviridae
11238	Myoviridae
11239	Siphoviridae
11240	Myoviridae
11241	Siphoviridae
11242	Unknown
11243	Unknown
11244	Unknown
11245	Unknown
11246	Myoviridae
11247	Siphoviridae
11248	Myoviridae
11249	Myoviridae
11250	Siphoviridae
11251	Siphoviridae
11252	Siphoviridae
11253	Podoviridae
11254	Siphoviridae
11255	Siphoviridae
11256	Siphoviridae
11257	Siphoviridae
11258	Myoviridae
11259	Siphoviridae
11260	Unknown
11261	Podoviridae
11262	Siphoviridae
11263	Siphoviridae
11264	Unknown
11265	Siphoviridae
11266	Siphoviridae
11267	Unknown
11268	Unknown
11269	Myoviridae
11270	Siphoviridae
11271	Unknown
11272	Siphoviridae
11273	Siphoviridae
11274	Siphoviridae
11275	Myoviridae
11276	Siphoviridae
11277	Myoviridae
11278	Unknown

dLGT_phageID	ICTV Phage taxa
11279	Unknown
11280	Podoviridae
11281	Podoviridae
11282	Unknown
11283	Podoviridae
11284	Siphoviridae
11285	Podoviridae
11286	Myoviridae
11287	Myoviridae
11288	Unknown
11289	Siphoviridae
11290	Siphoviridae
11291	Myoviridae
11292	Siphoviridae
11293	Siphoviridae
11294	Myoviridae
11295	Siphoviridae
11296	Siphoviridae
11297	Podoviridae
11298	Myoviridae
11299	Podoviridae
11300	Podoviridae
11301	Myoviridae
11302	Myoviridae
11303	Myoviridae
11304	Siphoviridae
11305	Siphoviridae
11306	Podoviridae
11307	Siphoviridae
11308	Siphoviridae
11309	Siphoviridae
11310	Siphoviridae
11311	Siphoviridae
11312	Podoviridae
11313	Unknown
11314	Myoviridae
11315	Myoviridae
11316	Siphoviridae
11317	Myoviridae
11318	Siphoviridae
11319	Siphoviridae
11320	Siphoviridae
11321	Siphoviridae
11322	Siphoviridae
11323	Siphoviridae

dLGT_phageID	ICTV Phage taxa
11324	Siphoviridae
11325	Myoviridae
11326	Myoviridae
11327	Unknown
11328	Myoviridae
11329	Siphoviridae
11330	Myoviridae
11331	Myoviridae
11332	Podoviridae
11333	Myoviridae
11334	Myoviridae
11335	Myoviridae
11336	Myoviridae
11337	Podoviridae
11338	Myoviridae
11339	Myoviridae
11340	Myoviridae
11341	Siphoviridae
11342	Myoviridae
11343	Siphoviridae
11344	Myoviridae
11345	Myoviridae
11346	Siphoviridae
11347	Myoviridae
11348	Myoviridae
11349	Myoviridae
11350	Siphoviridae
11351	Unknown
11352	Siphoviridae
11353	Myoviridae
11354	Inoviridae
11355	Siphoviridae
11356	Siphoviridae
11357	Siphoviridae
11358	Myoviridae
11359	Siphoviridae
11360	Siphoviridae
11361	Podoviridae
11362	Siphoviridae
11363	Myoviridae
11364	Myoviridae

Table S4. Detailed dLGT information for Fig. S3.

phage ID	prophage ID	donor	recipient	NCBI donor pid	NCBI recipient pid	gene product
11150	82542618_10	Shigella_boydii_Sb227_uid58215	Shigella_boydii_Sb227_uid58215	82546408	82544431	IS1 encoded protein
11150	82542618_10	Shigella_dysenteriae_1012_uid54365	Shigella_boydii_Sb227_uid58215	194433653	82544433	PTS system, glucose-specific IIBC component
11150	82542618_10	Shigella_boydii_CDC_3083_94_uid58415	Shigella_boydii_Sb227_uid58215	187733967	82544423	NADH dehydrogenase
11150	82542618_10	Shigella_boydii_CDC_3083_94_uid58415	Shigella_boydii_Sb227_uid58215	187732542	82544439	3-oxoacyl-(acyl carrier protein) synthase II
11150	82542618_10	Escherichia_coli_O26_H11_11368_uid41021	Shigella_boydii_Sb227_uid58215	260854549	82544468	ribosomal-protein-S5-alanine N-acetyltransferase
11150	82542618_10	Shigella_boydii_CDC_3083_94_uid58415	Shigella_boydii_Sb227_uid58215	187731599	82544416	outer membrane-specific lipoprotein transporter su
11150	82542618_10	Escherichia_albertii_TW07627_uid55089	Shigella_boydii_Sb227_uid58215	170768138	82544460	flagellar basal-body rod protein FlgC
11150	82542618_10	Escherichia_albertii_TW07627_uid55089	Shigella_boydii_Sb227_uid58215	170768270	82544446	hypothetical protein
11150	82542618_10	Escherichia_TW09308_uid80729	Shigella_boydii_Sb227_uid58215	366158789	82544429	purine nucleoside phosphoramidase
11150	82542618_10	Escherichia_coli_53638_uid54321	Shigella_boydii_Sb227_uid58215	188494203	82544485	glucans biosynthesis protein G
11150	82542618_10	Shigella_boydii_CDC_3083_94_uid58415	Shigella_boydii_Sb227_uid58215	187730351	82544425	beta-hexosaminidase
11150	82542618_10	Shigella_boydii_CDC_3083_94_uid58415	Shigella_boydii_Sb227_uid58215	187732240	82544414	outer membrane-specific lipoprotein transporter su
11150	82542618_10	Shigella_flexneri_2a_2457T_uid57991	Shigella_boydii_Sb227_uid58215	30062625	82544443	3-oxoacyl-ACP synthase
11150	82542618_10	Escherichia_coli_MS_196_1_uid50655	Shigella_boydii_Sb227_uid58215	301029721	161984918	transporter, major facilitator family protein
11150	82542618_10	Shigella_flexneri_5_8401_uid58583	Shigella_boydii_Sb227_uid58215	110805101	82544448	23S rRNA pseudouridylate synthase C
11150	82542618_10	Shigella_flexneri_2a_2457T_uid57991	Shigella_boydii_Sb227_uid58215	30062626	82544442	acyl carrier protein S-malonyltransferase
11150	82542618_10	Shigella_flexneri_2a_301_uid62907	Shigella_boydii_Sb227_uid58215	56479821	82544434	DNAse
11150	82542618_10	Salmonella_enterica_serovar_Typhi_404ty_uid55015	Shigella_boydii_Sb227_uid58215	213023792	82544445	50S ribosomal protein L32
11150	82542618_10	Escherichia_coli_CFT073_uid57915	Shigella_boydii_Sb227_uid58215	26247256	82544420	hypothetical protein
11150	82542618_10	Escherichia_coli_LF82_uid161965	Shigella_boydii_Sb227_uid58215	222155827	82544455	flagellar L-ring protein
11150	82542618_10	Shigella_boydii_CDC_3083_94_uid58415	Shigella_boydii_Sb227_uid58215	187730925	82544472	dihydroorotase
11150	82542618_10	Escherichia_coli_UMNK88_uid161991	Shigella_boydii_Sb227_uid58215	386613439	82544471	hypothetical protein
11150	82542618_10	Escherichia_coli_IAI1_uid59377	Shigella_boydii_Sb227_uid58215	218553638	82544474	N-methyltryptophan oxidase
11150	82542618_10	Shigella_boydii_CDC_3083_94_uid58415	Shigella_boydii_Sb227_uid58215	187732739	82544459	flagellar basal body rod modification protein
11150	82542618_10	Shigella_boydii_CDC_3083_94_uid58415	Shigella_boydii_Sb227_uid58215	187731130	82544457	flagellar basal body rod protein FlgF
11150	82542618_10	Escherichia_coli_B7A_uid54297	Shigella_boydii_Sb227_uid58215	191168486	82544464	flagella synthesis protein FlgN
11150	82542618_10	Shigella_boydii_CDC_3083_94_uid58415	Shigella_boydii_Sb227_uid58215	187733850	82544486	glucans biosynthesis protein
11150	82542618_10	Shigella_boydii_CDC_3083_94_uid58415	Shigella_boydii_Sb227_uid58215	187734244	82544458	flagellar hook protein FlgE
11150	82542618_10	Shigella_boydii_CDC_3083_94_uid58415	Shigella_boydii_Sb227_uid58215	187730861	82544428	putative lipoprotein
11150	82542618_10	Escherichia_coli_53638_uid54321	Shigella_boydii_Sb227_uid58215	188495951	82544467	hypothetical protein

phage ID	prophage ID	donor	recipient	NCBI donor pid	NCBI recipient pid	gene product
11150	82542618_10	Shigella_boydii_CDC_3083_94_uid58415	Shigella_boydii_Sb227_uid58215	187730975	82544419	LysM domain/ErfK/YbiS/YcfS/YnhG family protein
11150	82542618_10	Shigella_boydii_CDC_3083_94_uid58415	Shigella_boydii_Sb227_uid58215	187730870	82544417	putative acyltransferase
11150	82542618_10	Shigella_boydii_CDC_3083_94_uid58415	Shigella_boydii_Sb227_uid58215	187730939	82544452	flagellar hook-associated protein FlgL
11150	82542618_10	Shigella_boydii_CDC_3083_94_uid58415	Shigella_boydii_Sb227_uid58215	187732740	82544453	flagellar hook-associated protein FlgK
11150	82542618_10	Shigella_boydii_CDC_3083_94_uid58415	Shigella_boydii_Sb227_uid58215	187734148	82544426	thiamine kinase
11150	82542618_10	Escherichia_coli_O26_H11_11368_uid41021	Shigella_boydii_Sb227_uid58215	260854580	82544437	hypothetical protein
11150	82542618_10	Shigella_boydii_CDC_3083_94_uid58415	Shigella_boydii_Sb227_uid58215	187731717	82544432	ferric-rhodotorulic acid outer membrane transporte
11150	82542618_10	Shigella_boydii_CDC_3083_94_uid58415	Shigella_boydii_Sb227_uid58215	187730893	82544462	flagellar basal body P-ring biosynthesis protein F
11150	82542618_10	Shigella_boydii_CDC_3083_94_uid58415	Shigella_boydii_Sb227_uid58215	187731208	82544489	hypothetical protein
11150	82542618_10	Escherichia_coli_ED1a_uid59379	Shigella_boydii_Sb227_uid58215	218689466	82544395	putative tonB-like membrane protein from phage ori
11150	82542618_10	Escherichia_coli_O55_H7_CB9615_uid46655	Shigella_boydii_Sb227_uid58215	291282072	82544481	hypothetical protein
8390	120574166_2	Vibrio_cholerae_MO10_uid54325	Vibrio_cholerae_NCTC_8457_uid54589	254848889	153817802	hypothetical protein
8390	120574166_2	Vibrio_cholerae_MO10_uid54325	Vibrio_cholerae_NCTC_8457_uid54589	254848897	153817815	hypothetical protein
8390	120574166_2	Vibrio_cholerae_B33_uid54591	Vibrio_cholerae_NCTC_8457_uid54589	153821473	153817866	hypothetical protein
8390	120574166_2	Vibrio_cholerae_bv__albensis_VL426_uid55785	Vibrio_cholerae_NCTC_8457_uid54589	229526871	153817910	ATP/GTP-binding protein
8390	120574166_2	Vibrio_cholerae_bv__albensis_VL426_uid55785	Vibrio_cholerae_NCTC_8457_uid54589	229526870	153817902	group-specific protein, putative
8390	120574166_2	Vibrio_cholerae_TMA_21_uid55787	Vibrio_cholerae_NCTC_8457_uid54589	229513979	153817869	DNA repair protein RadA
8390	120574166_2	Vibrio_cholerae_MO10_uid54325	Vibrio_cholerae_NCTC_8457_uid54589	254849448	153817865	hypothetical protein
8390	120574166_2	Vibrio_cholerae_INDRE_91_1_uid41407	Vibrio_cholerae_NCTC_8457_uid54589	262161475	153817893	Galactose operon repressor
8390	120574166_2	Vibrio_cholerae_INDRE_91_1_uid41407	Vibrio_cholerae_NCTC_8457_uid54589	262161464	153817888	exodeoxyribonuclease V gamma chain
8390	120574166_2	Vibrio_cholerae_MJ_1236_uid59387	Vibrio_cholerae_NCTC_8457_uid54589	229607108	153817872	ribosomal-protein-S18p-alanine acetyltransferase
8390	120574166_2	Vibrio_cholerae_INDRE_91_1_uid41407	Vibrio_cholerae_NCTC_8457_uid54589	262161466	153817889	transcriptional regulator LysR family
8390	120574166_2	Vibrio_cholerae_O1_2010EL_1786_uid78933	Vibrio_cholerae_NCTC_8457_uid54589	360036186	153817862	cysteine desulfuration protein SufE
8390	120574166_2	Vibrio_cholerae_O395_uid58425	Vibrio_cholerae_NCTC_8457_uid54589	147673142	153817856	murein transglycosylase A
8390	120574166_2	Vibrio_cholerae_O1_2010EL_1786_uid78933	Vibrio_cholerae_NCTC_8457_uid54589	360036203	153817857	uncharacterized protein involved in response to NO
8390	120574166_2	Vibrio_cholerae_bv__albensis_VL426_uid55785	Vibrio_cholerae_NCTC_8457_uid54589	229524319	153817870	tellurite resistance protein
8390	120574166_2	Vibrio_cholerae_O1_2010EL_1786_uid78933	Vibrio_cholerae_NCTC_8457_uid54589	360036206	153817861	ribosomal protein S6 modification enzyme
8390	120574166_2	Vibrio_cholerae_INDRE_91_1_uid41407	Vibrio_cholerae_NCTC_8457_uid54589	262149060	153817849	aldehyde dehydrogenase
8390	120574166_2	Vibrio_cholerae_TM_11079_80_uid55813	Vibrio_cholerae_NCTC_8457_uid54589	229521158	153817882	hypothetical protein
8390	120574166_2	Vibrio_cholerae_bv__albensis_VL426_uid55785	Vibrio_cholerae_NCTC_8457_uid54589	229524303	153817871	AmpG permease
8390	120574166_2	Vibrio_cholerae_V52_uid54331	Vibrio_cholerae_NCTC_8457_uid54589	121727657	153817813	PTS system, fructose-specific IIB component

phage ID	prophage ID	donor	recipient	NCBI donor pid	NCBI recipient pid	gene product
8390	120574166_2	Vibrio_cholerae_B33_uid55791	Vibrio_cholerae_NCTC_8457_uid54589	229512209	153817859	beta-galactosidase
8390	120574166_2	Vibrio_cholerae_MJ_1236_uid59387	Vibrio_cholerae_NCTC_8457_uid54589	229607104	153817868	hypothetical protein
8390	120574166_2	Vibrio_cholerae_RC385_uid54327	Vibrio_cholerae_NCTC_8457_uid54589	297580941	153817867	peptidyl-prolyl cis-trans isomerase A
8390	120574166_2	Vibrio_cholerae_MO10_uid54325	Vibrio_cholerae_NCTC_8457_uid54589	254848905	153817816	hypothetical protein
8390	120574166_2	Vibrio_cholerae_INDRE_91_1_uid41407	Vibrio_cholerae_NCTC_8457_uid54589	262149061	153817852	sigma-54 dependent transcriptional regulator
8390	120574166_2	Vibrio_cholerae_MJ_1236_uid59387	Vibrio_cholerae_NCTC_8457_uid54589	229607645	153817784	sigma-54 dependent transcriptional regulator
8390	120574166_2	Vibrio_cholerae_MJ_1236_uid59387	Vibrio_cholerae_NCTC_8457_uid54589	229607099	153817854	hypothetical protein
8390	120574166_2	Vibrio_cholerae_V52_uid54331	Vibrio_cholerae_NCTC_8457_uid54589	121726775	153817860	bolA protein
8390	120574166_2	Vibrio_cholerae_V52_uid54331	Vibrio_cholerae_NCTC_8457_uid54589	121726750	153817858	4-methyl-5(B-hydroxyethyl)-thiazole monophosphate
8390	120574166_2	Vibrio_cholerae_MO10_uid54325	Vibrio_cholerae_NCTC_8457_uid54589	254848904	153817843	C factor cell-cell signaling protein
8390	120574166_2	Vibrio_cholerae_TM_11079_80_uid55813	Vibrio_cholerae_NCTC_8457_uid54589	229521156	153817878	hypothetical protein
8390	120574166_2	Vibrio_cholerae_2740_80_uid54567	Vibrio_cholerae_NCTC_8457_uid54589	121586204	153817877	hypothetical protein
8390	120574166_2	Vibrio_cholerae_TMA_21_uid55787	Vibrio_cholerae_NCTC_8457_uid54589	229513947	153817881	2-dehydropantoate 2-reductase
8390	120574166_2	Vibrio_cholerae_INDRE_91_1_uid41407	Vibrio_cholerae_NCTC_8457_uid54589	262147198	153817831	GTPase of unknown function subfamily, putative
8390	120574166_2	Vibrio_cholerae_O1_biovar_El_Tor_N16961_uid57623	Vibrio_cholerae_NCTC_8457_uid54589	15641812	153817803	hypothetical protein
8390	120574166_2	Vibrio_cholerae_O395_uid58425	Vibrio_cholerae_NCTC_8457_uid54589	147674352	153817804	DNA repair protein RadC
8390	120574166_2	Vibrio_cholerae_O395_uid58425	Vibrio_cholerae_NCTC_8457_uid54589	147673546	153817788	PTS system fructose-specific transporter subunit I
8390	120574166_2	Vibrio_cholerae_MJ_1236_uid59387	Vibrio_cholerae_NCTC_8457_uid54589	229607642	153817837	phosphotransferase system mannitol/fructose-specif
8390	120574166_2	Vibrio_cholerae_MZO_3_uid54569	Vibrio_cholerae_NCTC_8457_uid54589	153801706	153817914	RTX toxin activating protein
8390	120574166_2	Vibrio_ordalii_ATCC_33509_uid80489	Vibrio_cholerae_NCTC_8457_uid54589	365539589	153817807	hypothetical protein
8390	120574166_2	Vibrio_cholerae_MO10_uid54325	Vibrio_cholerae_NCTC_8457_uid54589	254848879	153817825	hypothetical protein
8390	120574166_2	Vibrio_cholerae_MO10_uid54325	Vibrio_cholerae_NCTC_8457_uid54589	254848879	153817846	hypothetical protein
8390	120574166_2	Vibrio_cholerae_MO10_uid54325	Vibrio_cholerae_NCTC_8457_uid54589	254848880	153817806	glyoxylase II family protein
8390	120574166_2	Vibrio_cholerae_IEC224_uid89389	Vibrio_cholerae_NCTC_8457_uid54589	379741623	153817840	hypothetical protein
8390	120574166_2	Vibrio_cholerae_MO10_uid54325	Vibrio_cholerae_NCTC_8457_uid54589	254850466	153817795	hypothetical protein
8390	120574166_2	Vibrio_cholerae_MO10_uid54325	Vibrio_cholerae_NCTC_8457_uid54589	254848885	153817829	hypothetical protein
8390	120574166_2	Vibrio_cholerae_MO10_uid54325	Vibrio_cholerae_NCTC_8457_uid54589	254848595	153818766	hypothetical protein
8390	120574166_2	Vibrio_cholerae_O1_biovar_El_Tor_N16961_uid57623	Vibrio_cholerae_NCTC_8457_uid54589	15641807	153817828	hypothetical protein
8390	120574166_2	Vibrio_cholerae_MAK_757_uid54571	Vibrio_cholerae_NCTC_8457_uid54589	298498447	153817908	hypothetical protein
8390	120574166_2	Vibrio_cholerae_MO10_uid54325	Vibrio_cholerae_NCTC_8457_uid54589	254848892	153817817	riboflavin biosynthesis protein ribAB
8390	120574166_2	Vibrio_cholerae_MO10_uid54325	Vibrio_cholerae_NCTC_8457_uid54589	254848881	153817834	mor transcription activator family
8390	120574166_2	Vibrio_cholerae_MO10_uid54325	Vibrio_cholerae_NCTC_8457_uid54589	254848891	153817824	hypothetical protein
8390	120574166_2	Vibrio_cholerae_MO10_uid54325	Vibrio_cholerae_NCTC_8457_uid54589	254848890	153817841	primosomal protein N

phage ID	prophage ID	donor	recipient	NCBI donor pid	NCBI recipient pid	gene product
8390	120574166_2	Vibrio_cholerae_INDRE_91_1_uid41407	Vibrio_cholerae_NCTC_8457_uid54589	262147196	153817820	hypothetical protein
8390	120574166_2	Vibrio_cholerae_MO10_uid54325	Vibrio_cholerae_NCTC_8457_uid54589	254848884	153817832	hypothetical protein
8390	120574166_2	Vibrio_cholerae_2740_80_uid54567	Vibrio_cholerae_NCTC_8457_uid54589	121585854	153817791	hypothetical protein
8390	120574166_2	Vibrio_cholerae_V52_uid54331	Vibrio_cholerae_NCTC_8457_uid54589	121728760	153817805	methyl-accepting chemotaxis protein
5739	209542188_1	Gluconacetobacter_diazotrophicus_PAl_5_uid61587	Gluconacetobacter_diazotrophicus_PAl_5_uid59075	162145886	209543806	hypothetical protein
5739	209542188_1	Gluconacetobacter_diazotrophicus_PAl_5_uid61587	Gluconacetobacter_diazotrophicus_PAl_5_uid59075	162145890	209543802	DNA uptake protein
5739	209542188_1	Gluconacetobacter_diazotrophicus_PAl_5_uid61587	Gluconacetobacter_diazotrophicus_PAl_5_uid59075	162145880	209543812	outer membrane protein
5739	209542188_1	Gluconacetobacter_diazotrophicus_PAl_5_uid61587	Gluconacetobacter_diazotrophicus_PAl_5_uid59075	162145902	209543792	virulence-associated protein
5739	209542188_1	Gluconacetobacter_diazotrophicus_PAl_5_uid61587	Gluconacetobacter_diazotrophicus_PAl_5_uid59075	162145908	209543786	1-(5-phosphoribosyl)-5-[(5-phosphoribosylamino)met
5739	209542188_1	Gluconacetobacter_diazotrophicus_PAl_5_uid61587	Gluconacetobacter_diazotrophicus_PAl_5_uid59075	162145879	209543813	LysR family transcriptional regulator
5739	209542188_1	Gluconacetobacter_diazotrophicus_PAl_5_uid61587	Gluconacetobacter_diazotrophicus_PAl_5_uid59075	162145887	209543805	dihydro-orotase
5739	209542188_1	Gluconacetobacter_diazotrophicus_PAl_5_uid61587	Gluconacetobacter_diazotrophicus_PAl_5_uid59075	162145878	209543814	hypothetical protein
5739	209542188_1	Gluconacetobacter_diazotrophicus_PAl_5_uid61587	Gluconacetobacter_diazotrophicus_PAl_5_uid59075	162145903	209543791	PilT protein domain-containing protein
5739	209542188_1	Gluconacetobacter_diazotrophicus_PAl_5_uid61587	Gluconacetobacter_diazotrophicus_PAl_5_uid59075	162145896	209543798	CRISPR-associated protein Cas2
5739	209542188_1	Gluconacetobacter_diazotrophicus_PAl_5_uid61587	Gluconacetobacter_diazotrophicus_PAl_5_uid59075	162145873	209543819	cobyric acid synthase
5739	209542188_1	Gluconacetobacter_diazotrophicus_PAl_5_uid61587	Gluconacetobacter_diazotrophicus_PAl_5_uid59075	162145900	209543794	Csd1 family CRISPR-associated protein
5739	209542188_1	Gluconacetobacter_diazotrophicus_PAl_5_uid61587	Gluconacetobacter_diazotrophicus_PAl_5_uid59075	162145889	209543803	glutamyl-tRNA synthetase
5739	209542188_1	Zymomonas_mobilis_NCIMB_11163_uid41019	Gluconacetobacter_diazotrophicus_PAl_5_uid59075	260753522	209543768	hypothetical protein
5739	209542188_1	Gluconacetobacter_diazotrophicus_PAl_5_uid61587	Gluconacetobacter_diazotrophicus_PAl_5_uid59075	162146899	209543824	hypothetical protein
5739	209542188_1	Gluconacetobacter_diazotrophicus_PAl_5_uid61587	Gluconacetobacter_diazotrophicus_PAl_5_uid59075	162145877	209543815	fusaric acid resistance protein
5739	209542188_1	Gluconacetobacter_diazotrophicus_PAl_5_uid61587	Gluconacetobacter_diazotrophicus_PAl_5_uid59075	162146230	209543764	hypothetical protein
5739	209542188_1	Gluconacetobacter_diazotrophicus_PAl_5_uid61587	Gluconacetobacter_diazotrophicus_PAl_5_uid59075	162145905	209543789	histidine triad (HIT) protein
5739	209542188_1	Gluconacetobacter_diazotrophicus_PAl_5_uid61587	Gluconacetobacter_diazotrophicus_PAl_5_uid59075	162145901	209543793	Cas5 family CRISPR-associated protein
5739	209542188_1	Gluconacetobacter_diazotrophicus_PAl_5_uid61587	Gluconacetobacter_diazotrophicus_PAl_5_uid59075	162145910	209543784	hypothetical protein
5739	209542188_1	Gluconacetobacter_diazotrophicus_PAl_5_uid61587	Gluconacetobacter_diazotrophicus_PAl_5_uid59075	162145885	209543807	DNA processing chain A
5739	209542188_1	Gluconacetobacter_diazotrophicus_PAl_5_uid61587	Gluconacetobacter_diazotrophicus_PAl_5_uid59075	162145898	209543796	CRISPR-associated protein Cas4
5739	209542188_1	Gluconacetobacter_diazotrophicus_PAl_5_uid61587	Gluconacetobacter_diazotrophicus_PAl_5_uid59075	162145875	209543817	phosphoglycerate mutase
5739	209542188_1	Gluconacetobacter_diazotrophicus_PAl_5_uid61587	Gluconacetobacter_diazotrophicus_PAl_5_uid59075	162145909	209543785	imidazole glycerol phosphate synthase subunit HisH
5739	162145846_1	Gluconacetobacter_SXCC_1_uid66379	Gluconacetobacter_diazotrophicus_PAl_5_uid61587	330990233	162146238	transcriptional regulator
5739	209542188_1	Gluconacetobacter_diazotrophicus_PAl_5_uid61587	Gluconacetobacter_diazotrophicus_PAl_5_uid59075	162145891	209543801	endonuclease III
5739	209542188_1	Gluconacetobacter_diazotrophicus_PAl_5_uid61587	Gluconacetobacter_diazotrophicus_PAl_5_uid59075	162145874	209543818	threonine-phosphate decarboxylase
5739	209542188_1	Gluconacetobacter_diazotrophicus_PAl_5_uid61587	Gluconacetobacter_diazotrophicus_PAl_5_uid59075	162145899	209543795	protein Csd2 family protein
5739	209542188_1	Gluconacetobacter_diazotrophicus_PAl_5_uid61587	Gluconacetobacter_diazotrophicus_PAl_5_uid59075	162145913	209543781	hypothetical protein

phage ID	prophage ID	donor	recipient	NCBI donor pid	NCBI recipient pid	gene product
5739	209542188_1	Gluconacetobacter_diazotrophicus_PAl_5_uid61587	Gluconacetobacter_diazotrophicus_PAl_5_uid59075	162145912	209543782	nitroreductase
5739	209542188_1	Gluconacetobacter_diazotrophicus_PAl_5_uid61587	Gluconacetobacter_diazotrophicus_PAl_5_uid59075	162145906	209543788	phosphoribosyl-ATP pyrophosphatase
5739	209542188_1	Gluconacetobacter_diazotrophicus_PAl_5_uid61587	Gluconacetobacter_diazotrophicus_PAl_5_uid59075	162145897	209543797	CRISPR-associated protein Cas1
5739	209542188_1	Gluconacetobacter_diazotrophicus_PAl_5_uid61587	Gluconacetobacter_diazotrophicus_PAl_5_uid59075	162146231	209543763	hypothetical protein
5739	209542188_1	Zymomonas_mobilis_NCIMB_11163_uid41019	Gluconacetobacter_diazotrophicus_PAl_5_uid59075	260753519	209543771	replication protein A
5739	209542188_1	Gluconacetobacter_diazotrophicus_PAl_5_uid61587	Gluconacetobacter_diazotrophicus_PAl_5_uid59075	162145911	209543783	imidazoleglycerol-phosphate dehydratase
5739	209542188_1	Gluconacetobacter_diazotrophicus_PAl_5_uid61587	Gluconacetobacter_diazotrophicus_PAl_5_uid59075	162145904	209543790	helicase
5739	209542188_1	Gluconacetobacter_diazotrophicus_PAl_5_uid61587	Gluconacetobacter_diazotrophicus_PAl_5_uid59075	162145881	209543811	hypothetical protein
5739	209542188_1	Gluconacetobacter_diazotrophicus_PAl_5_uid61587	Gluconacetobacter_diazotrophicus_PAl_5_uid59075	162145907	209543787	imidazole glycerol phosphate synthase subunit HisF
5739	209542188_1	Gluconacetobacter_diazotrophicus_PAl_5_uid61587	Gluconacetobacter_diazotrophicus_PAl_5_uid59075	162146229	209543765	hypothetical protein
10223	296493068_5	Escherichia_coli_UTI89_uid58541	Escherichia_coli_MS_198_1_uid50625	91206298	300897145	hypothetical protein
10223	296493068_5	Escherichia_coli_MS_21_1_uid50631	Escherichia_coli_MS_198_1_uid50625	300936966	300897120	hypothetical protein
10223	296493068_5	Escherichia_coli_OP50_uid49051	Escherichia_coli_MS_198_1_uid50625	297517181	300897097	putative hydrogenase 2 b cytochrome subunit
10223	296493068_5	Salmonella_enterica_serovar_Typhi_M223_uid55029	Escherichia_coli_MS_198_1_uid50625	213863268	300897102	hydrogenase 2 accessory protein HypG
10223	296493068_5	Escherichia_coli_OP50_uid49051	Escherichia_coli_MS_198_1_uid50625	297520571	300897098	hydrogenase 2 large subunit
10223	296493068_5	Escherichia_coli_S88_uid62979	Escherichia_coli_MS_198_1_uid50625	218560192	300897107	transporter
10223	296493068_5	Escherichia_coli_FVEC1412_uid46997	Escherichia_coli_MS_198_1_uid50625	293406617	300897091	yghZ protein
10223	296493068_5	Shigella_dysenteriae_1012_uid54365	Escherichia_coli_MS_198_1_uid50625	194431713	300897100	hydrogenase-2 operon protein HybE
10223	296493068_5	Escherichia_coli_H299_uid52527	Escherichia_coli_MS_198_1_uid50625	331684644	300897093	putative cytoplasmic protein
10223	296493068_5	Escherichia_coli_UMN026_uid62981	Escherichia_coli_MS_198_1_uid50625	218706618	300897101	hydrogenase nickel incorporation protein HybF
10223	296493068_5	Escherichia_coli_FVEC1412_uid46997	Escherichia_coli_MS_198_1_uid50625	293406720	300897110	pirin-like protein yhaK
10223	296493068_5	Escherichia_4_1_40B_uid55603	Escherichia_coli_MS_198_1_uid50625	386283159	300897137	toxin-antitoxin system, toxin component, MazF family
10223	296493068_5	Escherichia_1_1_43_uid55599	Escherichia_coli_MS_198_1_uid50625	404376324	300899251	hypothetical protein
10223	296493068_5	Escherichia_coli_MS_45_1_uid50643	Escherichia_coli_MS_198_1_uid50625	300973429	300897096	4Fe-4S binding domain protein
10223	296493068_5	Escherichia_coli_K_12_substr__MG1655_uid57779	Escherichia_coli_MS_198_1_uid50625	90111719	300897116	predicted oxidoreductase
10223	296493068_5	Escherichia_coli_IAI1_uid59377	Escherichia_coli_MS_198_1_uid50625	218555572	300897094	hypothetical protein
10223	296493068_5	Escherichia_coli_F11_uid54299	Escherichia_coli_MS_198_1_uid50625	191174392	300897125	hypothetical protein
10223	296493068_5	Escherichia_coli_UMN026_uid62981	Escherichia_coli_MS_198_1_uid50625	218692921	300897123	hypothetical protein
10223	296493068_5	Escherichia_coli_O7_K1_CE10_uid162115	Escherichia_coli_MS_198_1_uid50625	386627481	300897148	hypothetical protein
10223	296493068_5	Escherichia_coli_F11_uid54299	Escherichia_coli_MS_198_1_uid50625	191174388	300897133	plasmid segregation protein ParM
10223	296493068_5	Escherichia_coli_UMN026_uid62981	Escherichia_coli_MS_198_1_uid50625	218692931	300897135	hypothetical protein
10223	296493068_5	Escherichia_coli_MS_185_1_uid50657	Escherichia_coli_MS_198_1_uid50625	301046649	300897153	hypothetical protein

phage ID	prophage ID	donor	recipient	NCBI donor pid	NCBI recipient pid	gene product
10223	296493068_5	Escherichia_coli_SE15_uid161939	Escherichia_coli_MS_198_1_uid50625	281427932	300897144	hypothetical protein
10223	296493068_5	Escherichia_coli_FVEC1302_uid49707	Escherichia_coli_MS_198_1_uid50625	298378879	300897152	hypothetical protein
10223	296493068_5	Escherichia_coli_F11_uid54299	Escherichia_coli_MS_198_1_uid50625	191174391	300897140	hypothetical protein
10223	296493068_5	Escherichia_3_2_53FAA_uid55601	Escherichia_coli_MS_198_1_uid50625	237702611	300897154	adhesin biosynthesis transcription regulatory prot
10223	296493068_5	Escherichia_coli_SE15_uid161939	Escherichia_coli_MS_198_1_uid50625	281427933	300897139	hypothetical protein
10223	296493068_5	Escherichia_coli_SE15_uid161939	Escherichia_coli_MS_198_1_uid50625	281427941	300897131	truncated ImpB protein
10223	296493068_5	Escherichia_coli_O7_K1_CE10_uid162115	Escherichia_coli_MS_198_1_uid50625	386627482	300897147	hypothetical protein
5805	222441573_1	Bifidobacterium_pseudocatenulatum_DSM_20438_uid55303	Bifidobacterium_pseudocatenulatum_DSM_20438_uid55303	225352510	225352879	hypothetical protein
5805	157073726_1	Bifidobacterium_pseudocatenulatum_DSM_20438_uid55303	Parvimonas_micra_ATCC_33270_uid54527	225352882	160946082	hypothetical protein
6464	334145811_1	Prevotella_oris_F0302_uid55989	Porphyromonas_gingivalis_TDC60_uid67407	281426317	334147736	DNA binding domain, excisionase family protein
6464	255743520_1	Prevotella_veroralis_F0319_uid55991	Prevotella_veroralis_F0319_uid55991	260590890	260592901	DNA binding domain, excisionase family protein
6464	255743520_1	Prevotella_veroralis_F0319_uid55991	Prevotella_veroralis_F0319_uid55991	260590891	260592900	hypothetical protein
6464	334145811_1	Prevotella_oral_taxon_472_F0295_uid41061	Porphyromonas_gingivalis_TDC60_uid67407	260910276	334147747	hypothetical protein
5273	384177910_4	Bacillus_cereus_F837_76_uid83611	Bacillus_thuringiensis_serovar_finitimus_YBT_020_uid158875	376265158	384179262	2H phosphoesterase superfamily protein
5273	42779081_2	Bacillus_thuringiensis_MC28_uid176369	Bacillus_cereus_ATCC_10987_uid57673	407703678	42780413	Transporter, drug/metabolite exporter
5273	384177910_4	Bacillus_cereus_AH187_uid58753	Bacillus_thuringiensis_serovar_finitimus_YBT_020_uid158875	217958809	384179263	hypothetical protein
5273	222093774_1	Bacillus_cereus_H3081_97_uid54833	Bacillus_cereus_Q1_uid58529	206977683	222094951	membrane spanning protein
5273	222093774_1	Bacillus_cereus_G9241_uid54129	Bacillus_cereus_Q1_uid58529	47568420	222094941	streptomycin biosynthesis strf domain protein
5273	42779081_2	Bacillus_cereus_G9241_uid54129	Bacillus_cereus_ATCC_10987_uid57673	47568427	42780415	spore coat protein Y
5273	42779081_2	Bacillus_cereus_G9241_uid54129	Bacillus_cereus_ATCC_10987_uid57673	47568411	42780399	cell division protein ftsW
6299	323093298_5	Lactobacillus_ruminis_SPM0211_uid67955	Lactobacillus_ruminis_ATCC_25644_uid55509	335997655	323341546	prevent-host-death family antitoxin
6299	281406693_3	Roseburia_inulinivorans_DSM_16841_uid55375	Clostridium_M62_1_uid54557	225378529	283796742	hypothetical protein
6299	281406693_3	Clostridium_saccharolyticum_WM1_uid51419	Clostridium_M62_1_uid54557	302388005	291087260	MmcQ protein
6299	323093298_5	Lactobacillus_ruminis_ATCC_27782_uid73417	Lactobacillus_ruminis_ATCC_25644_uid55509	347524731	323341553	branched-chain amino acid transport protein
6299	281406693_3	Oribacterium_ACB1_uid79231	Clostridium_M62_1_uid54557	363899761	283796744	hypothetical protein
6299	281406693_3	Roseburia_inulinivorans_DSM_16841_uid55375	Clostridium_M62_1_uid54557	225378513	291087262	CDP-diacylglycerol--glycerol-3-phosphate 3-phospha
6299	281406693_3	Coprobacillus_8_2_54BFAA_uid82733	Clostridium_M62_1_uid54557	374625967	283796726	putative phospholipase D domain protein
6299	281406693_3	Eubacterium_ventriosum_ATCC_27560_uid54517	Clostridium_M62_1_uid54557	154484848	291087265	cardiolipin synthetase
6299	323093298_5	Lactobacillus_ruminis_SPM0211_uid67955	Lactobacillus_ruminis_ATCC_25644_uid55509	335997647	323341552	branched chain amino acid ABC superfamily ATP bind
6299	323093298_5	Lactobacillus_ruminis_SPM0211_uid67955	Lactobacillus_ruminis_ATCC_25644_uid55509	335997651	323341548	DegV family protein
6299	323093298_5	Lactobacillus_ruminis_ATCC_27782_uid73417	Lactobacillus_ruminis_ATCC_25644_uid55509	347524720	323341541	hypothetical protein

phage ID	prophage ID	donor	recipient	NCBI donor pid	NCBI recipient pid	gene product
6299	323093298_5	Lactobacillus_ruminis_SPM0211_uid67955	Lactobacillus_ruminis_ATCC_25644_uid55509	335997649	323341550	hypothetical protein
6299	281406693_3	Faecalibacterium_prausnitzii_A2_165_uid54551	Clostridium_M62_1_uid54557	257438013	291087264	putative permease
6299	323093298_5	Lactobacillus_ruminis_ATCC_27782_uid73417	Lactobacillus_ruminis_ATCC_25644_uid55509	347524727	323341549	hypothetical protein
6299	323093298_5	Lactobacillus_ruminis_SPM0211_uid67955	Lactobacillus_ruminis_ATCC_25644_uid55509	335997643	323341556	integral membrane protein
6299	281406693_3	Roseburia_inulinivorans_DSM_16841_uid55375	Clostridium_M62_1_uid54557	225378535	291087271	hypothetical protein
6299	323093298_5	Lactobacillus_ruminis_SPM0211_uid67955	Lactobacillus_ruminis_ATCC_25644_uid55509	335997648	323341551	putative H(+)-transporting two-sector ATPase
6299	323093298_5	Lactobacillus_ruminis_SPM0211_uid67955	Lactobacillus_ruminis_ATCC_25644_uid55509	335997644	323341555	hypothetical protein
6296	322417545_3	Desulfovibrio_aespoeensis_Aspo_2_uid42613	Geobacter_M18_uid55771	317153337	322420447	hypothetical protein
6296	116747452_2	Desulfarculus_baarsii_DSM_2075_uid51371	Syntrophobacter_fumaroxidans_MPOB_uid58177	302343227	116751236	hypothetical protein
6296	116747452_2	Desulfovibrio_aespoeensis_Aspo_2_uid42613	Syntrophobacter_fumaroxidans_MPOB_uid58177	317153327	116751226	hypothetical protein
6296	322417545_3	Desulfovibrio_alaskensis_G20_uid57941	Geobacter_M18_uid55771	78355972	322420457	hypothetical protein
6296	322417545_3	Dehalogenimonas_lykanthroporepellens_BL_DC_9_uid48131	Geobacter_M18_uid55771	300088748	322420456	hypothetical protein
6296	116747452_2	Desulfovibrio_aespoeensis_Aspo_2_uid42613	Syntrophobacter_fumaroxidans_MPOB_uid58177	317153332	116751231	hypothetical protein
6296	116747452_2	Desulfarculus_baarsii_DSM_2075_uid51371	Syntrophobacter_fumaroxidans_MPOB_uid58177	302343240	116751229	hypothetical protein
6296	322417545_3	Desulfovibrio_aespoeensis_Aspo_2_uid42613	Geobacter_M18_uid55771	317153330	322420454	hypothetical protein
6296	322417545_3	Desulfovibrio_alaskensis_G20_uid57941	Geobacter_M18_uid55771	78355974	322420455	hypothetical protein
6296	116747452_2	Desulfovibrio_aespoeensis_Aspo_2_uid42613	Syntrophobacter_fumaroxidans_MPOB_uid58177	317153329	116751228	hypothetical protein
6296	116747452_2	Dehalogenimonas_lykanthroporepellens_BL_DC_9_uid48131	Syntrophobacter_fumaroxidans_MPOB_uid58177	300088736	116751230	hypothetical protein
6296	322417545_3	Desulfarculus_baarsii_DSM_2075_uid51371	Geobacter_M18_uid55771	302343233	322420453	hypothetical protein
5548	108796981_2	Mycobacterium_JLS_uid58489	Mycobacterium_MCS_uid58465	126434215	108798616	aromatic-ring-hydroxylating dioxygenase subunit be
5548	108796981_2	Mycobacterium_JLS_uid58489	Mycobacterium_MCS_uid58465	126434216	108798617	sterol-binding domain-containing protein
5548	108796981_2	Mycobacterium_KMS_uid58491	Mycobacterium_MCS_uid58465	119867708	108798610	aromatic-ring-hydroxylating dioxygenase subunit be
5548	108796981_2	Mycobacterium_KMS_uid58491	Mycobacterium_MCS_uid58465	119867709	108798611	ring hydroxylating dioxygenase subunit alpha
5548	108796981_2	Mycobacterium_KMS_uid58491	Mycobacterium_MCS_uid58465	119867713	108798615	Rieske (2Fe-2S) domain-containing protein
5548	108796981_2	Mycobacterium_KMS_uid58491	Mycobacterium_MCS_uid58465	119867712	108798614	hypothetical protein
5548	108796981_2	Mycobacterium_KMS_uid58491	Mycobacterium_MCS_uid58465	119867720	108798624	virulence factor Mce family protein
5548	108796981_2	Mycobacterium_KMS_uid58491	Mycobacterium_MCS_uid58465	119867716	108798618	intradiol ring-cleavage dioxygenase
5548	120401028_2	Mycobacterium_KMS_uid58491	Mycobacterium_vanbaalenii_PYR_1_uid58463	119867718	120401521	hypothetical protein
5548	120401028_2	Mycobacterium_gilvum_PYR_GCK_uid59421	Mycobacterium_vanbaalenii_PYR_1_uid58463	145225985	120401526	hypothetical protein
5548	120401028_2	Mycobacterium_vanbaalenii_PYR_1_uid58463	Mycobacterium_vanbaalenii_PYR_1_uid58463	120406356	120401525	hypothetical protein
5548	120401028_2	Mycobacterium_vanbaalenii_PYR_1_uid58463	Mycobacterium_vanbaalenii_PYR_1_uid58463	120406360	120401529	hypothetical protein
5548	120401028_2	Mycobacterium_vanbaalenii_PYR_1_uid58463	Mycobacterium_vanbaalenii_PYR_1_uid58463	120406363	120401532	hypothetical protein
5434	330822653_3	Ralstonia_5_2_56FAA_uid73851	Alicycliphilus_denitrificans_K601_uid66307	404394087	330824604	hypothetical protein
5434	308922585_1	Ralstonia_5_2_56FAA_uid73851	Ralstonia_5_7_47FAA_uid59461	404394102	309780983	hypothetical protein
5434	330822653_3	Ralstonia_5_2_56FAA_uid73851	Alicycliphilus_denitrificans_K601_uid66307	404394101	330824618	hypothetical protein

phage ID	prophage ID	donor	recipient	NCBI donor pid	NCBI recipient pid	gene product
5434	308922585_1	Ralstonia_5_2_56FAA_uid73851	Ralstonia_5_7_47FAA_uid59461	404394092	309780994	hypothetical protein
5434	330822653_3	Ralstonia_5_2_56FAA_uid73851	Alicycliphilus_denitrificans_K601_uid66307	404394092	330824609	hypothetical protein
5434	308922585_1	Ralstonia_5_2_56FAA_uid73851	Ralstonia_5_7_47FAA_uid59461	404394097	309780989	hypothetical protein
5434	330822653_3	Ralstonia_5_2_56FAA_uid73851	Alicycliphilus_denitrificans_K601_uid66307	404394097	330824614	hypothetical protein
5434	308922585_1	Ralstonia_5_2_56FAA_uid73851	Ralstonia_5_7_47FAA_uid59461	404394081	309781005	hypothetical protein
5434	330822653_3	Ralstonia_5_2_56FAA_uid73851	Alicycliphilus_denitrificans_K601_uid66307	404394081	330824598	hypothetical protein
5434	308922585_1	Ralstonia_5_2_56FAA_uid73851	Ralstonia_5_7_47FAA_uid59461	404394090	309780996	hypothetical protein
5434	330822653_3	Ralstonia_5_2_56FAA_uid73851	Alicycliphilus_denitrificans_K601_uid66307	404394090	330824607	hypothetical protein
5434	308922585_1	Ralstonia_5_2_56FAA_uid73851	Ralstonia_5_7_47FAA_uid59461	404394091	309780995	hypothetical protein
5434	330822653_3	Ralstonia_5_2_56FAA_uid73851	Alicycliphilus_denitrificans_K601_uid66307	404394091	330824608	hypothetical protein
5434	308922585_1	Ralstonia_5_2_56FAA_uid73851	Ralstonia_5_7_47FAA_uid59461	404394078	309781008	hypothetical protein
5434	330822653_3	Ralstonia_5_2_56FAA_uid73851	Alicycliphilus_denitrificans_K601_uid66307	404394078	330824595	hypothetical protein
5434	308922585_1	Ralstonia_5_2_56FAA_uid73851	Ralstonia_5_7_47FAA_uid59461	404394080	309781006	holliday junction resolvasome, endonuclease subuni
5434	330822653_3	Ralstonia_5_2_56FAA_uid73851	Alicycliphilus_denitrificans_K601_uid66307	404394080	330824597	hypothetical protein
5434	308922585_1	Ralstonia_5_2_56FAA_uid73851	Ralstonia_5_7_47FAA_uid59461	404394086	309781000	hypothetical protein
5434	330822653_3	Ralstonia_5_2_56FAA_uid73851	Alicycliphilus_denitrificans_K601_uid66307	404394086	330824603	hypothetical protein
5434	308922585_1	Ralstonia_5_2_56FAA_uid73851	Ralstonia_5_7_47FAA_uid59461	404394082	309781004	hypothetical protein
5434	330822653_3	Ralstonia_5_2_56FAA_uid73851	Alicycliphilus_denitrificans_K601_uid66307	404394082	330824599	hypothetical protein
5434	308922585_1	Ralstonia_5_2_56FAA_uid73851	Ralstonia_5_7_47FAA_uid59461	404394099	309780987	hypothetical protein
5434	330822653_3	Ralstonia_5_2_56FAA_uid73851	Alicycliphilus_denitrificans_K601_uid66307	404394099	330824616	hypothetical protein
5434	330822653_3	Ralstonia_5_2_56FAA_uid73851	Alicycliphilus_denitrificans_K601_uid66307	404394088	330824605	hypothetical protein
5434	308922585_1	Ralstonia_5_2_56FAA_uid73851	Ralstonia_5_7_47FAA_uid59461	404394088	309780998	bacteriophage protein
5009	167533223_7	Bacillus_anthracis_Kruger_B_uid54105	Bacillus_anthracis_A0442_uid54997	254740813	167636120	hypothetical protein
5009	172084907_5	Bacillus_anthracis_Kruger_B_uid54105	Bacillus_anthracis_A0174_uid55003	254740821	177654415	dTDP-4-dehydrorhamnose 3,5-epimerase
5009	228860913_1	Bacillus_7_6_55CFAA_CT2_uid80427	Bacillus_thuringiensis_serovar_andalousiensis_BGSC_4AW1_uid55231	365162026	228932602	Membrane spanning protein
5009	170130696_6	Bacillus_anthracis_Kruger_B_uid54105	Bacillus_anthracis_A0389_uid54999	254740825	170708888	hypothetical protein
5009	30260195_2	Bacillus_anthracis_A0465_uid55001	Bacillus_anthracis_Ames_uid57909	170686744	30261321	hypothetical protein
5009	229599883_2	Bacillus_thuringiensis_serovar_huazhongensis_BGSC_4BD1_uid55235	Bacillus_anthracis_A0248_uid59385	228920033	229603926	BclA protein
5009	228860886_2	Bacillus_thuringiensis_serovar_chinensis_CT_43_uid158151	Bacillus_cereus_95_8201_uid55175	384185223	229120830	cell division protein ftsW
5009	386733873_2	Bacillus_thuringiensis_serovar_pulsiensis_BGSC_4CC1_uid55237	Bacillus_anthracis_H9401_uid162021	228913898	386735035	hypothetical protein
5008	387619774_1	Escherichia_coli_P12b_uid162061	Escherichia_coli_DH1_uid162051	386703230	387619791	Na(+)/H(+) antiporter NhaA
5008	188491671_7	Escherichia_coli_MS_196_1_uid50655	Escherichia_coli_53638_uid54321	301024697	188492225	GPR1/FUN34/YaaH family protein
5008	170018061_7	Escherichia_coli_O26_H11_11368_uid41021	Escherichia_coli_ATCC_8739_uid58783	260853222	170021630	molybdenum cofactor biosynthesis protein MogA
5008	253771435_8	Shigella_sonnei_53G_uid84383	Escherichia_coli__BL21_Gold_DE3_pLysS_AG__uid59245	383176593	253774959	molybdenum cofactor biosynthesis protein MogA

phage ID	prophage ID	donor	recipient	NCBI donor pid	NCBI recipient pid	gene product
5008	74310614_1	Shigella_sonnei_53G_uid84383	Shigella_sonnei_Ss046_uid58217	383176595	74310625	hypothetical protein
5008	387610477_1	Escherichia_1_1_43_uid55599	Escherichia_coli_ETEC_H10407_uid161993	404373328	387610489	UPF0412 protein yaal
5008	157159467_1	Escherichia_coli_E24377A_uid58395	Escherichia_coli_HS_uid58393	157157283	157159480	hypothetical protein
6260	316906148_1	Bacteroides_vulgatus_PC510_uid47771	Bacteroides_4_1_36_uid61871	294776407	317481406	hypothetical protein
6260	156709644_7	Clostridium_hathewayi_DSM_13479_uid55373	Clostridium_bolteae_ATCC_BAA_613_uid54523	288870461	160941792	hypothetical protein
6260	316906148_1	Bacteroides_vulgatus_PC510_uid47771	Bacteroides_4_1_36_uid61871	294776408	317481405	hypothetical protein
6260	156709644_7	Lachnospiraceae_bacterium_3_1_57FAA_CT1_uid68201	Clostridium_bolteae_ATCC_BAA_613_uid54523	336430315	160941795	hypothetical protein
6260	316906148_1	Bacteroides_vulgatus_PC510_uid47771	Bacteroides_4_1_36_uid61871	294776402	317481408	hypothetical protein
6260	316906148_1	Bacteroides_dorei_DSM_17855_uid54993	Bacteroides_4_1_36_uid61871	212692397	317481419	hypothetical protein
6260	156709644_7	Lachnospiraceae_bacterium_3_1_57FAA_CT1_uid68201	Clostridium_bolteae_ATCC_BAA_613_uid54523	336430314	160941797	hypothetical protein
6260	316906148_1	Bacteroides_1_1_30_uid68191	Bacteroides_4_1_36_uid61871	336407257	317481404	hypothetical protein
6260	316906148_1	Clostridium_difficile_NAP08_uid49121	Bacteroides_4_1_36_uid61871	296451591	317481416	hypothetical protein
6260	316906148_1	Bacteroides_2_1_16_uid41707	Bacteroides_4_1_36_uid61871	265763429	317481421	type I restriction endonuclease S subunit
6260	156709644_7	Clostridium_hathewayi_DSM_13479_uid55373	Clostridium_bolteae_ATCC_BAA_613_uid54523	288870462	160941790	hypothetical protein
5915	255741500_6	Ruminococcus_gnavus_ATCC_29149_uid54537	Blautia_hansenii_DSM_20583_uid55275	154504531	260590110	hypothetical protein
5915	255741500_6	Ruminococcus_gnavus_ATCC_29149_uid54537	Blautia_hansenii_DSM_20583_uid55275	154504532	260590109	racemase, Asp/Glu/Hydantoin family
5915	255741500_6	Ruminococcus_gnavus_ATCC_29149_uid54537	Blautia_hansenii_DSM_20583_uid55275	154504540	260590101	hypothetical protein
5915	146337174_3	Lachnospiraceae_bacterium_2_1_58FAA_uid68203	Ruminococcus_gnavus_ATCC_29149_uid54537	336434353	154504593	hypothetical protein
5915	255741500_6	Ruminococcus_gnavus_ATCC_29149_uid54537	Blautia_hansenii_DSM_20583_uid55275	154504541	260590100	hypothetical protein
5915	255741500_6	Ruminococcus_gnavus_ATCC_29149_uid54537	Blautia_hansenii_DSM_20583_uid55275	154504533	260590108	hypothetical protein
5915	255741500_6	Ruminococcus_gnavus_ATCC_29149_uid54537	Blautia_hansenii_DSM_20583_uid55275	154504539	260590102	toxin-antitoxin system, toxin component, PIN famil
5915	255741500_6	Ruminococcus_gnavus_ATCC_29149_uid54537	Blautia_hansenii_DSM_20583_uid55275	154504534	260590107	hypothetical protein
5915	255741500_6	Ruminococcus_gnavus_ATCC_29149_uid54537	Blautia_hansenii_DSM_20583_uid55275	154504537	260590104	hypothetical protein
5915	146337174_3	Lachnospiraceae_bacterium_2_1_58FAA_uid68203	Ruminococcus_gnavus_ATCC_29149_uid54537	336434356	154504590	hypothetical protein
5915	146337174_3	Lachnospiraceae_bacterium_2_1_58FAA_uid68203	Ruminococcus_gnavus_ATCC_29149_uid54537	336434357	154504589	GTP-sensing transcriptional pleiotropic repressor
5915	146337174_3	Clostridium_7_3_54FAA_uid76577	Ruminococcus_gnavus_ATCC_29149_uid54537	355626871	154504564	hypothetical protein
5915	255741500_6	Clostridium_7_3_54FAA_uid76577	Blautia_hansenii_DSM_20583_uid55275	355626871	260590077	hydroxymethylglutaryl-CoA synthase
5915	255741500_6	Ruminococcus_gnavus_ATCC_29149_uid54537	Blautia_hansenii_DSM_20583_uid55275	154504538	260590103	putative single-strand DNA binding protein
5915	255741500_6	Ruminococcus_gnavus_ATCC_29149_uid54537	Blautia_hansenii_DSM_20583_uid55275	154504530	260590111	integrase, catalytic domain protein
5915	255741500_6	Ruminococcus_gnavus_ATCC_29149_uid54537	Blautia_hansenii_DSM_20583_uid55275	154504535	260590106	hypothetical protein

phage ID	prophage ID	donor	recipient	NCBI donor pid	NCBI recipient pid	gene product
5915	255741500_6	Ruminococcus_gnavus_ATCC_29149_uid54537	Blautia_hansenii_DSM_20583_uid55275	154504536	260590105	putative long-chain-fatty-acid-CoA ligase

Table S5: Toxin/Antitoxin genes in the dLGT network.

donor	recipient	donor ncbi pid	recipient ncbi pid	phage id	prophage GI	product
Escherichia_coli_M718_uid52481	Escherichia_coli_K_12_substr__MG1655_uid57779	331652942	16129522	5036	300713132_1	Qin prophage; toxin of the RelE-RelB toxin-antitox
Synechococcus_elongatus_PCC_6301_uid58235	Synechococcus_elongatus_PCC_7942_uid58045	56750352	81300016	8221	384516725_1	addiction module toxin, Txe/YoeB
Lactobacillus_casei_Zhang_uid50673	Lactobacillus_casei_ATCC_334_uid57985	301065561	116493973	8352	81298811_2	holin-like toxin
Shewanella_baltica_OS185_uid58743	Shewanella_baltica_OS195_uid58261	152998496	160873056	5631	332304386_1	bifunctional antitoxin/transcriptional repressor R
Shewanella_baltica_OS223_uid58775	Shewanella_baltica_OS195_uid58261	217980314	160873057	5631	384541581_12	addiction module antitoxin
Gluconacetobacter_europaeus_LMG_18494_uid73763	Gluconacetobacter_diazotrophicus_PAl_5_uid61587	349702420	162146883	8711	384541581_12	bifunctional antitoxin/transcriptional repressor R
Gluconacetobacter_diazotrophicus_PAl_5_uid59075	Gluconacetobacter_diazotrophicus_PAl_5_uid61587	209543908	162146884	8711	219870279_1	relE protein (antitoxin)
Escherichia_coli_M718_uid52481	Escherichia_coli_ATCC_8739_uid58783	331652943	170020084	5675	386311792_1	bifunctional antitoxin/transcriptional repressor R
Escherichia_coli_M718_uid52481	Escherichia_coli_ATCC_8739_uid58783	331652942	170020085	5675	397671778_1	addiction module antitoxin
Xylella_fastidiosa_sandyi_Ann_1_uid54099	Xylella_fastidiosa_M23_uid58809	71902523	182681706	8803	384545966_5	addiction module antitoxin
Acinetobacter_NBRC_100985_uid78547	Acinetobacter_baumannii_ACICU_uid58765	359430914	184159998	8810	170018061_4	cytotoxic translational repressor of toxin-antitox
Ralstonia_eutropha_JMP134_uid58047	Cupriavidus_taiwanensis_LMG_19424_uid61615	72384150	188591803	8908	260853213_8	toxin of toxin-antitoxin stability system, PIN dom
Cupriavidus_necator_N_1_uid68689	Cupriavidus_taiwanensis_LMG_19424_uid61615	339328095	188591804	8908	295697839_1	Antitoxin of toxin-antitoxin system, phd type
Haemophilus_parasuis_29755_uid54869	Haemophilus_parasuis_SH0165_uid59273	167856641	219870823	9252	386707734_5	addiction module antitoxin
Haemophilus_parasuis_29755_uid54869	Haemophilus_parasuis_SH0165_uid59273	167856640	219870824	9252	386307442_6	RelB, addiction module antitoxin/DNA-damage-induci
Erwinia_Ejp617_uid159955	Erwinia_pyrifoliae_Ep1_96_uid40659	385786480	259909524	5930	388476123_6	Addiction module toxin
Escherichia_coli_M718_uid52481	Escherichia_coli_O26_H11_1136	331652942	260855301	5971	260853213_8	toxin of the RelE-RelB toxin-antitoxin

donor	recipient	donor ncbi pid	recipient ncbi pid	phage id	prophage GI	product
	8_uid41021					system
Escherichia_coli_M718_uid52481	Escherichia_coli_O26_H11_11368_uid41021	331652943	260855302	5971	170018061_4	bifunctional antitoxin/transcriptional repressor R
Escherichia_coli_M718_uid52481	Enterobacter_cloacae_ATCC_13047_uid48363	331652943	295697964	10197	49175990_7	bifunctional antitoxin/transcriptional repressor R
Serratia_M24T3_uid158323	Erwinia_billingiae_Eb661_uid50547	383817446	300713277	10389	384516725_1	transcriptional regulator/antitoxin
Lactobacillus_coryniformis_torquens_KCTC_3535_uid68113	Lactobacillus_buchneri_NRRL_B_30929_uid66205	336391587	330370673	10831	386307331_2	addiction module toxin, RelE/StbE family
Alteromonas_macleodii__Deep_ecotype__uid58251	Glaciecola_4H_3_7_YE_5_uid66595	332140023	332306633	10863	389867183_5	RelE/StbE family addiction module toxin
Metallosphaera_yellowstonensis_MK1_uid82737	Acidianus_hospitalis_W1_uid66875	374631339	332796099	10879	386307442_6	VapC-type toxin
Acetobacter_aceti_NBRC_14818_uid70715	Gluconacetobacter_xylinus_NBRC_3288_uid46523	340778970	347762057	6578	389867183_3	addiction module toxin Txe/YoeB
Enterococcus_faecalis_TUSoD_Ef11_uid55455	Enterococcus_faecalis_62_uid159663	300861720	384517487	6835	385850283_3	zeta toxin family protein
Enterococcus_faecalis_TX0109_uid52591	Enterococcus_faecalis_62_uid159663	307288927	384517488	6835	385852231_3	epsilon antitoxin family protein
Escherichia_coli_042_uid161985	Shigella_flexneri_2002017_uid159233	387610009	384544532	6854	385854193_1	Toxin subunit Set1B
Escherichia_coli_042_uid161985	Shigella_flexneri_2002017_uid159233	387610010	384544533	6854	384545966_5	Toxin subunit Set1A
Shigella_dysenteriae_1012_uid54365	Shigella_flexneri_2002017_uid159233	194435265	384546211	6869	330370666_1	post-segregation antitoxin
Shigella_dysenteriae_1012_uid54365	Shigella_flexneri_2002017_uid159233	194435263	384546212	6869	386083114_6	post-segregation toxin
Neisseria_meningitidis_MC58_uid57817	Neisseria_meningitidis_M04_240196_uid162081	15676809	385851429	6951	182680572_5	putative plasmid toxin protein PemI
Neisseria_meningitidis_MC58_uid57817	Neisseria_meningitidis_H44_76_uid162083	15676809	385853395	5061	332795693_1	putative plasmid toxin protein PemI
Neisseria_meningitidis_MC58_uid57817	Neisseria_meningitidis_M01_240355_uid162075	15676809	385855047	6952	259906682_4	putative plasmid toxin protein PemI
Xylella_fastidiosa_sandyi_Ann_1_uid54099	Xylella_fastidiosa_GB514_uid162023	71902523	386085196	6986	162145846_2	addiction module toxin, RelE/StbE family
Yersinia_enterocolitica_palearctica_105_5R_r__uid63663	Yersinia_enterocolitica_palearctica_Y11_uid162069	332163613	386307378	6990	162145846_2	ParE toxin protein

donor	recipient	donor ncbi pid	recipient ncbi pid	phage id	prophage GI	product
Yersinia_enterocolitica_palearctica_105_5R_r__uid63663	Yersinia_enterocolitica_palearctica_Y11_uid162069	332160516	386311458	6997	219870279_1	toxin mRNA interferase YgiU
Yersinia_enterocolitica_palearctica_105_5R_r__uid63663	Yersinia_enterocolitica_palearctica_Y11_uid162069	332160517	386311459	6997	184159988_1	antitoxin YgiT
Pseudoalteromonas_BSi20311_uid78647	Shewanella_putrefaciens_200_uid161927	359438784	386314349	6998	347762033_1	RelB/DinJ family addiction module antitoxin
Escherichia_blattae_DSM_4481_uid165043	Escherichia_coli_W_uid162101	387888747	386709416	5060	160873002_1	bifunctional antitoxin/transcriptional repressor R
Escherichia_coli_MS_21_1_uid50631	Escherichia_coli_K_12_substr__W3110_uid161931	300939058	388477639	5036	116493574_1	toxin of the RelE-RelB toxin-antitoxin system
Enterococcus_faecium_1_230_933_uid55701	Enterococcus_faecium_DO_uid55353	257878361	389868680	11095	160873002_1	prevent-host-death family antitoxin
Enterococcus_faecium_U0317_uid47349	Enterococcus_faecium_DO_uid55353	294621854	389869056	11096	188591327_1	prevent-host-death family antitoxin
Mycobacterium_tuberculosis_KZN_4207_uid83619	Mycobacterium_tuberculosis_H37Rv_uid170532	375294812	397672400	7253	188591327_1	antitoxin
Escherichia_4_1_40B_uid55603	Escherichia_coli_MS_198_1_uid50625	386283159	300897137	10223	296493068_5	cytotoxic protein CcdB

Table S6. Antibiotic resistance genes in the dLGT. Most of the genes are transferred between closely related donors and recipients, with a majority of Bacilli and Gammaproteobacteria donors and recipients. For example, we identified a phage-mediated LGT of a bleomycin resitance gene that is transferred from *Staphylococcus aureus* MRSA252 to *Staphylococcus aureus* JKD6009, both strains are methicillin-resistant clinical strains that where isolated from MRSA bactermia patients. A total of 22 (47%) antibiotic resistance gene transfers are inter-generic, including a phage that connects the two domains (PhageID: 9888).

Donor	Recipient	NCBI donor pid	NCBI recipient pid	Phage ID	Prophage ID	Gene product
Enterococcus_faecium_1_230_933_uid55701	Enterococcus_faecium_1_231_502_uid55713	257880581	257883593	9701	239912833_4	aminoglycoside N-acetyltransferase/aminoglycoside
Salmonella_enterica_serovar_Typhi_E98_0664_uid55023	Salmonella_enterica_serovar_Typhi_E98_2068_uid55025	213579731	213612546	8865	188194814_5	acriflavin resistance protein B
Bacteroides_eggerthii_DSM_20697_uid54989	Klebsiella_pneumoniae_rhinoscleromatis_ATCC_13884_uid41361	218132122	262043815	11288	259042952_10	tetracycline resistance protein
Escherichia_coli_1827_70_uid60615	Shigella_dysenteriae_1617_uid59463	312973382	309784916	11337	308928589_18	positive transcription regulator evgA
Staphylococcus_epidermidis_SK135_uid42967	Staphylococcus_epidermidis_SK135_uid42967	282875743	282874817	7305	281296747_1	putative lincosamide resistance protein
Escherichia_coli_K_12_substr__W3110_uid161931	Escherichia_coli_MS_21_1_uid50631	388479880	300935217	10270	296496862_2	DNA-binding transcriptional dual regulator
Escherichia_coli_OP50_uid49051	Escherichia_coli_FVEC1302_uid49707	297518448	298380261	6056	291162641_3	DNA-binding transcriptional regulator PhoP
Escherichia_coli_E110019_uid54303	Shigella_flexneri_2002017_uid159233	193071717	384542778	6860	384541581_5	DNA-binding response regulator PhoP
Shigella_dysenteriae_Sd197_uid58213	Shigella_dysenteriae_1617_uid59463	161950043	309785320	10552	308928589_9	outer membrane channel protein
Shigella_flexneri_2a_2457T_uid57991	Shigella_flexneri_2002017_uid159233	30062676	384542777	6860	384541581_5	sensor protein PhoQ
Shigella_boydii_CDC_3083_94_uid58415	Shigella_dysenteriae_Sd197_uid58213	187733890	82779495	7329	82775382_17	DNA-binding transcriptional activator EvgA
Erysipelotrichaceae_bacterium_3_1_53_uid59459	Enterococcus_faecium_1_230_933_uid55701	309775385	257880618	9713	239923133_4	rRNA adenine N-6-methyltransferase
Streptococcus_oralis_Uo5_uid65449	Enterococcus_faecalis_Merz96_uid55691	331267237	256959519	9702	239912944_1	ribosomal RNA adenine N-6-methyltransferaseadenine
Staphylococcus_aureus_MSSA476_uid57841	Staphylococcus_aureus_ATCC_51811_uid49431	49398113	297208855	10302	296887982_3	beta-lactamase precursor
Escherichia_coli_MS_145_7_uid59467	Escherichia_coli_MS_124_1_uid50763	309798321	301307521	11323	299856628_16	phosphotransferase enzyme family protein

Donor	Recipient	NCBI donor pid	NCBI recipient pid	Phage ID	Prophage ID	Gene product
Vibrio_cholerae_MO10_uid54325	Escherichia_coli_MS_84_1_uid50623	254850916	300905658	10234	296493501_9	phosphotransferase enzyme family protein
Aeromonas_salmonicida_A449_uid58631	Escherichia_coli_MS_107_1_uid50575	145301311	300819973	10351	299856451_8	integrase/recombinase
Staphylococcus_aureus_JKD6008_uid159855	Staphylococcus_aureus_JKD6008_uid159855	384862253	384861481	6880	384860682_2	rRNA adenine N-6-methyltransferase, ErmA
Salmonella_enterica_serovar_Choleraesuis_SC_B67_uid58017	Acinetobacter_baumannii_TCDC_AB0715_uid158679	60115532	385235783	6895	385235550_1	beta-lactamase
Salmonella_enterica_serovar_Choleraesuis_SC_B67_uid58017	Escherichia_coli_MS_124_1_uid50763	60115532	301307411	11321	299856628_14	beta-lactamase
Macrococcus_caseolyticus_JCSC5402_uid59003	Enterococcus_faecalis_TX0104_uid55351	222142617	227519922	9321	223714737_8	bifunctional aminoglycoside N-acetyltransferase an
Macrococcus_caseolyticus_JCSC5402_uid59003	Staphylococcus_epidermidis_RP62A_uid57663	222142617	57867487	8129	57865352_1	bifunctional aminoglycoside N-acetyltransferase an
Macrococcus_caseolyticus_JCSC5402_uid59003	Staphylococcus_aureus_A9765_uid42999	222142617	282929650	9974	282594749_5	bifunctional aminoglycoside N-acetyltransferase an
Acinetobacter_6013150_uid47499	Escherichia_coli_MS_124_1_uid50763	332856476	301307414	11321	299856628_14	phosphotransferase enzyme family protein
Acinetobacter_6013150_uid47499	Escherichia_coli_BW2952_uid59391	332856476	238903096	9682	238899406_8	phosphotransferase enzyme family protein
Acinetobacter_6013150_uid47499	Escherichia_coli_MS_84_1_uid50623	332856476	300905201	10233	296493501_8	phosphotransferase enzyme family protein
Staphylococcus_aureus_MRSA252_uid57839	Staphylococcus_aureus_A8819_uid48133	49482285	295407741	10187	294970273_6	bleomycin resistance protein
Staphylococcus_aureus_MRSA252_uid57839	Staphylococcus_aureus_A8796_uid49425	49482285	297246819	10308	297179164_7	bleomycin resistance protein
Staphylococcus_aureus_MRSA252_uid57839	Staphylococcus_aureus_JKD6009_uid55147	49482285	221140581	8960	194045409_2	bleomycin resistance protein
Bacillus_cereus_BGSC_6E1_uid55157	Bacillus_cereus_F837_76_uid83611	229185478	376267122	5473	376264031_1	Vancomycin response regulator VanR
Enterococcus_faecalis_S613_uid46987	Enterococcus_faecium_E1071_uid47015	293387040	293567292	10135	291590108_1	D-Ala-D-Ala dipeptidase
Enterococcus_faecium_E1679_uid47347	Enterococcus_faecium_E1071_uid47015	294617750	293567293	10135	291590108_1	vancomycin/teicoplanin A-type resistance protein V
Acinetobacter_baumannii_AYE_uid61637	Klebsiella_pneumoniae_HS11286_uid84387	169797578	378975898	6693	378975832_1	chloramphenicol and florfenicol resistance protein
Pasteurella_multocida_36950_uid86887	Salmonella_enterica_serovar_Typhimurium_T000240_uid84397	378773998	378987328	6703	378987264_1	aminoglycoside 3''-O-adenyltransferase protein
Pasteurella_multocida_36950_uid86887	Klebsiella_pneumoniae_HS11286_uid84387	378773998	378975901	6693	378975832_1	aminoglycoside 3''-O-adenyltransferase protein
Staphylococcus_aureus_CF_Marseille	Staphylococcus_aureus_A9765_uid42	253316335	282929333	9974	282594749_5	ErmC

Donor	Recipient	NCBI donor pid	NCBI recipient pid	Phage ID	Prophage ID	Gene product
_uid55083	999					
Staphylococcus_aureus_CF_Marseille_uid55083	Staphylococcus_aureus_A6300_uid55929	253316335	258445548	9830	257856245_2	ErmC
Staphylococcus_aureus_A9719_uid55937	Staphylococcus_aureus_A8796_uid49425	258421113	297246682	10306	297179164_5	macrolide 2'-phosphotransferase II
Acinetobacter_baumannii_AB059_uid50775	Acinetobacter_6014059_uid47503	301597789	332875881	10878	332739367_5	gentamicin 3'-N-acetyltransferase
Escherichia_coli_MS_84_1_uid50623	Acinetobacter_6014059_uid47503	300906161	332875875	10878	332739367_5	hypothetical protein
Escherichia_coli_MS_84_1_uid50623	Escherichia_coli_MS_107_1_uid50575	300906161	300819964	10350	299856451_7	conserved domain protein
Bacillus_cereus_Rock3_28_uid55183	Methanobrevibacter_smithii_DSM_2374_uid55123	229107540	261351063	9888	260533258_1	Tetracycline resistance protein, class A
Acinetobacter_baumannii_TYTH_1_uid176498	Acinetobacter_baumannii_TCDC_AB0715_uid158679	407933339	385235793	6895	385235550_1	chloramphenicol acetyltransferase
Escherichia_coli_H591_uid52525	Escherichia_coli_FVEC1412_uid46997	331676922	293404485	6056	291093227_4	transcriptional regulatory protein PhoP
Enterococcus_faecium_TX0133a04_uid61129	Enterococcus_faecium_TX0133A_uid61139	314939834	314953627	5158	306477232_6	chloramphenicol O-acetyltransferase
Salmonella_enterica_serovar_Choleraesuis_SC_B67_uid58017	Escherichia_coli_SMS_3_5_uid58919	60115525	170650787	8775	170650760_1	chloramphenicol acetyl transferase II
Escherichia_coli_MS_78_1_uid50771	Bacteroides_capillosus_ATCC_29799_uid54531	301329505	154500894	8507	146337129_3	chloramphenicol O-acetyltransferase

Table S7. Detailed information of the phages that are connected to different bacterial phyla in the D-dLGT network.

Donor genome	Donor phylum	Phage ID	Recipient genome	Recipient phylum	Recipient pid	Gene product
Thermoanaerobacter_X561_uid55835	Firmicutes	5388	Thermoanaerobacter_X514_uid58589	Firmicutes	82544410	lipoprotein signal peptidase
Fervidobacterium_nodosum_Rt17_B1_uid58625	Thermotogae	5388	Thermoanaerobacter_X514_uid58589	Firmicutes	170770015	recombinase
Oxalobacteraceae_bacterium_IMCC9480_uid66143	Proteobacteria	5606	Janthinobacterium_Marseille_uid58603	Proteobacteria	152982840	hypothetical protein
Candidatus_Nitrospira_defluvii_uid51175	Nitrospirae	5606	Janthinobacterium_Marseille_uid58603	Proteobacteria	300898681	hypothetical protein
Fusobacterium_necrophorum_funduliforme_1_1_36S_uid81603	Fusobacteria	5812	Fusobacterium_3_1_5R_uid55607	Fusobacteria	317059607	conserved hypothetical protein
Fusobacterium_necrophorum_funduliforme_1_1_36S_uid81603	Fusobacteria	5812	Fusobacterium_3_1_5R_uid55607	Fusobacteria	317059619	predicted protein
Fusobacterium_D12_uid55613	Fusobacteria	5812	Fusobacterium_3_1_5R_uid55607	Fusobacteria	317058306	predicted protein
Fusobacterium_D12_uid55613	Fusobacteria	5812	Fusobacterium_3_1_5R_uid55607	Fusobacteria	317058298	predicted protein
Fusobacterium_D12_uid55613	Fusobacteria	5812	Fusobacterium_3_1_5R_uid55607	Fusobacteria	317059622	predicted protein
Oribacterium_oral_taxon_078_F0262_uid55773	Firmicutes	5812	Fusobacterium_3_1_5R_uid55607	Fusobacteria	170681061	predicted protein
Clostridium_nexile_DSM_1787_uid55077	Firmicutes	6188	Mobiluncus_mulieris_ATCC_35243_uid55889	Actinobacteria	170769264	cpp2 protein
Mobiluncus_curtisii_ATCC_35241_uid51715	Actinobacteria	6188	Mobiluncus_mulieris_ATCC_35243_uid55889	Actinobacteria	82775887	hypothetical protein
Mobiluncus_curtisii_ATCC_35241_uid51715	Actinobacteria	6188	Mobiluncus_mulieris_ATCC_35239_uid52345	Actinobacteria	300937022	hypothetical protein
Mobiluncus_curtisii_ATCC_35241_uid51715	Actinobacteria	6188	Mobiluncus_mulieris_ATCC_35243_uid55889	Actinobacteria	261258494	hypothetical protein
Mobiluncus_curtisii_ATCC_35241_uid51715	Actinobacteria	6188	Mobiluncus_mulieris_ATCC_35239_uid52345	Actinobacteria	261258494	hypothetical protein
Bacteroides_vulgatus_PC510_uid47771	Bacteroidetes	6260	Bacteroides_4_1_36_uid61871	Bacteroidetes	317481406	hypothetical protein
Clostridium_hathewayi_DSM_13479_uid55373	Firmicutes	6260	Clostridium_bolteae_ATCC_BAA_613_uid54523	Firmicutes	160941792	hypothetical protein
Bacteroides_vulgatus_PC510_uid47771	Bacteroidetes	6260	Bacteroides_4_1_36_uid61871	Bacteroidetes	317481405	hypothetical protein
Lachnospiraceae_bacterium_3_1_57FAA_CT1_uid68201	Firmicutes	6260	Clostridium_bolteae_ATCC_BAA_613_uid54523	Firmicutes	213620859	hypothetical protein
Bacteroides_vulgatus_PC510_uid47771	Bacteroidetes	6260	Bacteroides_4_1_36_uid61871	Bacteroidetes	386618360	hypothetical protein
Bacteroides_dorei_DSM_17855_uid54993	Bacteroidetes	6260	Bacteroides_4_1_36_uid61871	Bacteroidetes	208822242	hypothetical protein
Lachnospiraceae_bacterium_3_1_57FAA_CT1_uid68201	Firmicutes	6260	Clostridium_bolteae_ATCC_BAA_613_uid54523	Firmicutes	208822242	hypothetical protein
Bacteroides_1_1_30_uid68191	Bacteroidetes	6260	Bacteroides_4_1_36_uid61871	Bacteroidetes	56479642	hypothetical protein
Clostridium_difficile_NAP08_uid49121	Firmicutes	6260	Bacteroides_4_1_36_uid61871	Bacteroidetes	82776518	hypothetical protein
Bacteroides_2_1_16_uid41707	Bacteroidetes	6260	Bacteroides_4_1_36_uid61871	Bacteroidetes	331648362	type I restriction endonuclease S subunit
Clostridium_hathewayi_DSM_13479_uid55373	Firmicutes	6260	Clostridium_bolteae_ATCC_BAA_613_uid54523	Firmicutes	383179743	hypothetical protein
Desulfovibrio_aespoeens	Proteobacteria	6296	Geobacter_M18_uid	Proteobacteria	384541869	hypothetical protein

Donor genome	Donor phylum	Phage ID	Recipient genome	Recipient phylum	Recipient pid	Gene product
is_Aspo_2_uid42613			55771			
Desulfarculus_baarsii_DSM_2075_uid51371	Proteobacteria	6296	Syntrophobacter_fumaroxidans_MPOB_uid58177	Proteobacteria	384541869	hypothetical protein
Desulfovibrio_aespoeensis_Aspo_2_uid42613	Proteobacteria	6296	Syntrophobacter_fumaroxidans_MPOB_uid58177	Proteobacteria	384541869	hypothetical protein
Desulfovibrio_alaskensis_G20_uid57941	Proteobacteria	6296	Geobacter_M18_uid55771	Proteobacteria	384541869	hypothetical protein
Dehalogenimonas_lykanthroporepellens_BL_DC_9_uid48131	Chloroflexi	6296	Geobacter_M18_uid55771	Proteobacteria	56479642	hypothetical protein
Desulfovibrio_aespoeensis_Aspo_2_uid42613	Proteobacteria	6296	Syntrophobacter_fumaroxidans_MPOB_uid58177	Proteobacteria	56479642	hypothetical protein
Desulfarculus_baarsii_DSM_2075_uid51371	Proteobacteria	6296	Syntrophobacter_fumaroxidans_MPOB_uid58177	Proteobacteria	309785952	hypothetical protein
Desulfovibrio_aespoeensis_Aspo_2_uid42613	Proteobacteria	6296	Geobacter_M18_uid55771	Proteobacteria	309785952	hypothetical protein
Desulfovibrio_alaskensis_G20_uid57941	Proteobacteria	6296	Geobacter_M18_uid55771	Proteobacteria	309785952	hypothetical protein
Desulfovibrio_aespoeensis_Aspo_2_uid42613	Proteobacteria	6296	Syntrophobacter_fumaroxidans_MPOB_uid58177	Proteobacteria	82775887	hypothetical protein
Dehalogenimonas_lykanthroporepellens_BL_DC_9_uid48131	Chloroflexi	6296	Syntrophobacter_fumaroxidans_MPOB_uid58177	Proteobacteria	170770015	hypothetical protein
Desulfarculus_baarsii_DSM_2075_uid51371	Proteobacteria	6296	Geobacter_M18_uid55771	Proteobacteria	170770015	hypothetical protein
Fusobacterium_necrophorum_funduliforme_1_1_36S_uid81603	Fusobacteria	6441	Streptococcus_anginosus_SK52_uid67811	Firmicutes	335031492	hypothetical protein
Streptococcus_anginosus_1_2_62CV_uid62163	Firmicutes	6441	Streptococcus_anginosus_F0211_uid61277	Firmicutes	208808103	CDP-diacylglycerol-glycerol-3-phosphate 3-phosphat
Streptococcus_anginosus_SK52_uid67811	Firmicutes	6441	Streptococcus_anginosus_F0211_uid61277	Firmicutes	209397358	tryptophan--tRNA ligase
Oribacterium_oral_taxon_108_F0425_uid67819	Firmicutes	6441	Streptococcus_anginosus_SK52_uid67811	Firmicutes	56479642	conserved domain protein
Streptococcus_anginosus_SK52_uid67811	Firmicutes	6441	Streptococcus_anginosus_F0211_uid61277	Firmicutes	331662814	flavodoxin-like protein
Streptococcus_anginosus_1_2_62CV_uid62163	Firmicutes	6441	Streptococcus_anginosus_SK52_uid67811	Firmicutes	218688891	transcription regulator yrfE
Eubacteriaceae_bacterium_ACC19a_uid79227	Firmicutes	6441	Streptococcus_anginosus_SK52_uid67811	Firmicutes	213612547	MobA/MobL family protein
Streptococcus_anginosus_1_2_62CV_uid62163	Firmicutes	6441	Streptococcus_anginosus_F0211_uid61277	Firmicutes	213586943	ABC transporter
Streptococcus_anginosus_1_2_62CV_uid62163	Firmicutes	6441	Streptococcus_anginosus_F0211_uid61277	Firmicutes	170681061	triose-phosphate isomerase
Streptococcus_dysgalactiae_equisimilis_ATCC_12394_uid161979	Firmicutes	6441	Streptococcus_anginosus_SK52_uid67811	Firmicutes	300825178	hypothetical protein
Escherichia_coli_H736_uid52485	Proteobacteria	7081	Escherichia_coli_P12b_uid162061	Proteobacteria	386704706	putative cold-shock protein
Escherichia_coli_FVEC1302_uid49707	Proteobacteria	7081	Escherichia_coli_P12b_uid162061	Proteobacteria	167995215	cold shock-like protein cspF
Escherichia_coli_H736_uid52485	Proteobacteria	7081	Escherichia_coli_P12b_uid162061	Proteobacteria	195938768	protein FlxA
Escherichia_coli_101_1_uid54363	Proteobacteria	7081	Escherichia_coli_P12b_uid162061	Proteobacteria	309785876	Y4bF
Enterococcus_faecalis_ARO1_DG_uid55687	Firmicutes	7081	Escherichia_coli_P12b_uid162061	Proteobacteria	387882004	Prophage Qin DNA packaging protein NU1-

Donor genome	Donor phylum	Phage ID	Recipient genome	Recipient phylum	Recipient pid	Gene product
						like protei
Bacteroides_capillosus_ATCC_29799_uid54531	Firmicutes	8507	Bacteroides_capillosus_ATCC_29799_uid54531	Firmicutes	154500899	hypothetical protein
Clostridium_clostridioforme_2_1_49FAA_uid76955	Firmicutes	8507	Bacteroides_capillosus_ATCC_29799_uid54531	Firmicutes	154500901	hypothetical protein
Bacteroides_capillosus_ATCC_29799_uid54531	Firmicutes	8507	Bacteroides_capillosus_ATCC_29799_uid54531	Firmicutes	154500908	hypothetical protein
Ruminococcaceae_bacterium_D16_uid52825	Firmicutes	8507	Bacteroides_capillosus_ATCC_29799_uid54531	Firmicutes	154500888	carboxymuconolactone decarboxylase
Bacteroides_capillosus_ATCC_29799_uid54531	Firmicutes	8507	Bacteroides_capillosus_ATCC_29799_uid54531	Firmicutes	154500911	hypothetical protein
Ruminococcaceae_bacterium_D16_uid52825	Firmicutes	8507	Bacteroides_capillosus_ATCC_29799_uid54531	Firmicutes	154500867	hypothetical protein
Faecalibacterium_prausnitzii_A2_165_uid54551	Firmicutes	8507	Bacteroides_capillosus_ATCC_29799_uid54531	Firmicutes	213620859	putative nickase
Anaerotruncus_colihominis_DSM_17241_uid54807	Firmicutes	8507	Bacteroides_capillosus_ATCC_29799_uid54531	Firmicutes	213620859	hypothetical protein
Anaerotruncus_colihominis_DSM_17241_uid54807	Firmicutes	8507	Bacteroides_capillosus_ATCC_29799_uid54531	Firmicutes	309785093	hypothetical protein
Enterococcus_faecium_1_230_933_uid55701	Firmicutes	8507	Bacteroides_capillosus_ATCC_29799_uid54531	Firmicutes	213612547	hypothetical protein
Flavonifractor_plautii_ATCC_29863_uid80691	Firmicutes	8507	Bacteroides_capillosus_ATCC_29799_uid54531	Firmicutes	74311440	hypothetical protein
Flavonifractor_plautii_ATCC_29863_uid80691	Firmicutes	8507	Bacteroides_capillosus_ATCC_29799_uid54531	Firmicutes	74311440	hypothetical protein
Flavonifractor_plautii_ATCC_29863_uid80691	Firmicutes	8507	Bacteroides_capillosus_ATCC_29799_uid54531	Firmicutes	188493236	hypothetical protein
Ruminococcaceae_bacterium_D16_uid52825	Firmicutes	8507	Bacteroides_capillosus_ATCC_29799_uid54531	Firmicutes	237707137	flavoredoxin
Bacteroides_capillosus_ATCC_29799_uid54531	Firmicutes	8507	Bacteroides_capillosus_ATCC_29799_uid54531	Firmicutes	386701455	hypothetical protein
Escherichia_coli_MS_78_1_uid50771	Proteobacteria	8507	Bacteroides_capillosus_ATCC_29799_uid54531	Firmicutes	383179743	chloramphenicol O-acetyltransferase
Anaerotruncus_colihominis_DSM_17241_uid54807	Firmicutes	8704	Anaerotruncus_colihominis_DSM_17241_uid54807	Firmicutes	167771746	hypothetical protein
Clostridium_M62_1_uid54557	Firmicutes	8704	Anaerotruncus_colihominis_DSM_17241_uid54807	Firmicutes	213620859	topoisomerase IV subuni A
Treponema_succinifaciens_DSM_2489_uid65781	Spirochaetes	8704	Anaerotruncus_colihominis_DSM_17241_uid54807	Firmicutes	298380263	hypothetical protein
Clostridium_M62_1_uid54557	Firmicutes	9283	Bacteroides_9_1_42FAA_uid55587	Bacteroidetes	300940019	rop protein
Cardiobacterium_hominis_ATCC_15826_uid55949	Proteobacteria	9283	Bacteroides_9_1_42FAA_uid55587	Bacteroidetes	386618360	type IV secretory pathway, VirB4 component
Bacteroides_3_1_33FAA_uid41705	Bacteroidetes	9283	Bacteroides_9_1_42FAA_uid55587	Bacteroidetes	15830754	conserved hypothetical protein
Bacteroides_3_1_33FAA_uid41705	Bacteroidetes	9283	Bacteroides_9_1_42FAA_uid55587	Bacteroidetes	312964989	conserved hypothetical protein
Anaerococcus_tetradius_ATCC_35098_uid55461	Firmicutes	9295	Anaerococcus_tetradius_ATCC_35098_uid55461	Firmicutes	227501387	hypothetical protein

Donor genome	Donor phylum	Phage ID	Recipient genome	Recipient phylum	Recipient pid	Gene product
Gardnerella_vaginalis_409_05_uid43211	Actinobacteria	9295	Anaerococcus_tetradius_ATCC_35098_uid55461	Firmicutes	227500834	hypothetical protein
Corynebacterium_accolens_ATCC_49726_uid52361	Actinobacteria	9296	Corynebacterium_accolens_ATCC_49725_uid55467	Actinobacteria	227502969	LysR-family transcriptional regulator
Clostridiales_bacterium_1_7_47FAA_uid55287	Firmicutes	9296	Corynebacterium_accolens_ATCC_49725_uid55467	Actinobacteria	227502955	hypothetical protein
Fusobacterium_3_1_27_uid47791	Fusobacteria	9380	Staphylococcus_epidermidis_W23144_uid55899	Firmicutes	242243091	hypothetical protein
Staphylococcus_epidermidis_ATCC_12228_uid57861	Firmicutes	9380	Staphylococcus_epidermidis_W23144_uid55899	Firmicutes	386598005	glutamine-binding periplasmic protein
Enhydrobacter_aerosaccus_SK60_uid55439	Proteobacteria	9454	Streptococcus_salivarius_SK126_uid55863	Firmicutes	228476675	hypothetical protein
Enhydrobacter_aerosaccus_SK60_uid55439	Proteobacteria	9454	Streptococcus_salivarius_SK126_uid55863	Firmicutes	228476674	hypothetical protein
Streptococcus_salivarius_JIM8777_uid162145	Firmicutes	9454	Streptococcus_salivarius_SK126_uid55863	Firmicutes	218688891	rod shape-determining protein MreD
Streptococcus_salivarius_JIM8777_uid162145	Firmicutes	9454	Streptococcus_salivarius_SK126_uid55863	Firmicutes	238901779	rod shape-determining protein MreC
Proteus_penneri_ATCC_35198_uid54897	Proteobacteria	9888	Methanobrevibacter_smithii_DSM_2374_uid55123	Euryarchaeota	168778020	hypothetical protein
Bacillus_cereus_Rock3_28_uid55183	Firmicutes	9888	Methanobrevibacter_smithii_DSM_2374_uid55123	Euryarchaeota	56479642	Tetracycline resistance protein, class A
Proteus_penneri_ATCC_35198_uid54897	Proteobacteria	9976	Citrobacter_youngae_ATCC_29220_uid55081	Proteobacteria	168778020	hypothetical protein
Clostridium_M62_1_uid54557	Firmicutes	9976	Citrobacter_youngae_ATCC_29220_uid55081	Proteobacteria	300940019	rop protein
Clostridium_cellulovorans_743B_uid51503	Firmicutes	10428	Mobiluncus_curtisii_ATCC_35241_uid51715	Actinobacteria	213620859	TnpV protein
Arcanobacterium_haemolyticum_DSM_20595_uid49489	Actinobacteria	10428	Mobiluncus_curtisii_ATCC_35241_uid51715	Actinobacteria	168239590	hypothetical protein
Subdoligranulum_4_3_54A2FAA_uid80415	Firmicutes	10675	Ethanoligenens_harbinense_YUAN_3_uid46255	Firmicutes	298380263	hypothetical protein
Parascardovia_denticolens_F0305_uid47801	Actinobacteria	10675	Ethanoligenens_harbinense_YUAN_3_uid46255	Firmicutes	74311440	hypothetical protein
Ureaplasma_urealyticum_serovar_9_ATCC_33175_uid54651	Tenericutes	10769	Anaerococcus_hydrogenalis_ACS_025_V_Sch4_uid63589	Firmicutes	325849288	conserved domain protein
Finegoldia_magna_BVS033A4_uid51529	Firmicutes	10769	Anaerococcus_hydrogenalis_ACS_025_V_Sch4_uid63589	Firmicutes	325849279	hypothetical protein
Peptoniphilus_duerdenii_ATCC_BAA_1640_uid51739	Firmicutes	10769	Anaerococcus_hydrogenalis_ACS_025_V_Sch4_uid63589	Firmicutes	82776666	hypothetical protein
Pasteurella_bettyae_CCUG_2042_uid165735	Proteobacteria	10999	Streptococcus_constellatus_pharyngis_SK1060_uid72195	Firmicutes	343525250	hypothetical protein
Pasteurella_bettyae_CCUG_2042_uid165735	Proteobacteria	10999	Streptococcus_constellatus_pharyngis_SK1060_uid72195	Firmicutes	343525212	hypothetical protein
Gemella_moribillum_M424_uid61881	Firmicutes	10999	Streptococcus_constellatus_pharyngis_SK1060_uid72195	Firmicutes	309785093	tetR family bacterial regulatory protein
Bulleidia_extructa_W1219_uid43197	Firmicutes	10999	Streptococcus_constellatus_pharyngis_S	Firmicutes	298380263	TraG/TraD domain protein

Donor genome	Donor phylum	Phage ID	Recipient genome	Recipient phylum	Recipient pid	Gene product
			K1060_uid72195			
Bulleidia_extructa_W1219_uid43197	Firmicutes	10999	Streptococcus_constellatus_pharyngis_SK1060_uid72195	Firmicutes	331662814	TIGR02185 family protein
Bacteroides_eggerthii_DSM_20697_uid54989	Bacteroidetes	11288	Klebsiella_pneumoniae_rhinoscleromatis_ATCC_13884_uid41361	Proteobacteria	262043815	tetracycline resistance protein
Klebsiella_4_1_44FAA_uid80417	Proteobacteria	11288	Klebsiella_pneumoniae_rhinoscleromatis_ATCC_13884_uid41361	Proteobacteria	262043832	hypothetical protein
Cardiobacterium_hominis_ATCC_15826_uid55949	Proteobacteria	11288	Klebsiella_pneumoniae_rhinoscleromatis_ATCC_13884_uid41361	Proteobacteria	386618360	type IV secretory pathway, VirB4 component
Escherichia_coli_NA114_uid162139	Proteobacteria	11308	Escherichia_coli_MS_198_1_uid50625	Proteobacteria	300901684	hypothetical protein
Escherichia_coli_NA114_uid162139	Proteobacteria	11308	Escherichia_coli_MS_198_1_uid50625	Proteobacteria	168778020	hypothetical protein
Escherichia_coli_B171_uid54319	Proteobacteria	11308	Escherichia_coli_MS_198_1_uid50625	Proteobacteria	300820148	hypothetical protein
Escherichia_coli_NA114_uid162139	Proteobacteria	11308	Escherichia_coli_MS_198_1_uid50625	Proteobacteria	82775887	hypothetical protein
Escherichia_coli_O111_H__11128_uid41023	Proteobacteria	11308	Escherichia_coli_MS_198_1_uid50625	Proteobacteria	194429955	hypothetical protein
Lactobacillus_vaginalis_ATCC_49540_uid55515	Firmicutes	11308	Escherichia_coli_MS_198_1_uid50625	Proteobacteria	168789133	hypothetical protein
Lactobacillus_vaginalis_ATCC_49540_uid55515	Firmicutes	11308	Escherichia_coli_MS_198_1_uid50625	Proteobacteria	168789133	hypothetical protein

Table S8. Distribution of synonymous (*dS*) and non-synonymous substitution (*dN*). (A) Distribution of donor vs. recipient *dN, dS* and *dN/dS* at several levels. Upper gray box shows the amount of genes that have values of *dN* and/or *dS* equal to 0. Lower gray box is indicating the comparison of genes having at least one synonymous and non-synonymous substitution. (B) Percentiles of the observed and expected donor and recipient *dN* and *dS.*

A

		**donor**							
		***dS* = 0, *dN* = 0**	***dS* > 0, *dN* = 0**	***dS*=0,*dN*>0**	**0 < *dN/dS* <= 0.1**	**0.1 < *dN/dS* <= 0.5**	**0.5 < *dN/dS* <= 1**	***dN/dS* >1**	**total**
**recipient**	***dS* = 0, *dN* = 0**	5075	408	322	310	209	36	9	6369
	***dS* > 0, *dN* = 0**	419	190	100	128	84	7	2	930
	***dS* = 0, *dN* > 0**	237	61	65	41	44	4	1	453
	**0 < *dN/dS* <= 0.1**	336	110	69	592	167	10	1	1285
	**0.1 < *dN/dS* <= 0.5**	161	95	59	136	187	12	7	657
	**0.5 < *dN/dS* <= 1**	24	8	8	6	14	5	0	65
	***dN/dS* > 1**	10	1	3	0	1	2	2	19
	**total**	6262	873	626	1213	706	76	22	9778

B

	observed				expected			
**percentile**	***dS***		***dN***		***dS***		***dN***	
	**donor**	**recipient**	**donor**	**recipient**	**donor**	**recipient**	**donor**	**recipient**
0	0.0000	0.0000	0.0000	0.0000	0.0000	0.0000	0.0000	0.0000
10	0.0000	0.0000	0.0000	0.0000	0.0000	0.0000	0.0000	0.0000
20	0.0000	0.0000	0.0000	0.0000	0.0000	0.0000	0.0000	0.0000
30	0.0000	0.0000	0.0000	0.0000	0.0002	0.0002	0.0000	0.0000
40	0.0000	0.0000	0.0000	0.0000	0.0005	0.0005	0.0000	0.0000
50	0.0000	0.0000	0.0000	0.0000	0.0008	0.0008	0.0000	0.0000
60	0.0000	0.0000	0.0000	0.0000	0.0015	0.0016	0.0000	0.0000
70	0.0000	0.0029	0.0000	0.0000	0.9861	1.0053	0.0000	0.0000
80	0.0227	0.0246	0.0018	0.0012	2.2531	2.5088	1.0022	0.9726
**90**	0.0780	0.0898	0.0063	0.0066	8.3780	9.5768	2.0374	2.0565
**91**	0.0890	0.1051	0.0072	0.0074	9.7660	11.1560	2.0733	2.5609
**92**	0.1065	0.1231	0.0081	0.0087	11.6540	13.2100	2.6490	3.0624
**93**	0.1228	0.1480	0.0092	0.0102	13.9530	15.8400	3.0523	3.5539
**94**	0.1497	0.1810	0.0105	0.0120	17.0120	19.2560	3.3868	4.1731
**95**	0.1795	0.2208	0.0122	0.0139	20.9450	24.2520	4.1892	5.1564
**96**	0.2156	0.2742	0.0144	0.0166	27.0870	31.3960	5.1257	6.2427
**97**	0.2845	0.3429	0.0173	0.0207	35.4710	42.1160	6.3543	8.1058
**98**	0.3851	0.4487	0.0217	0.0252	53.1820	63.9460	8.4833	10.5450
**99**	0.6115	0.7311	0.0286	0.0335	94.1360	108.3300	13.5860	16.8490
**100**	144.4800	144.9800	0.0652	0.1012	14418.0000	23052.0000	84.9560	124.5700
